# Protocol for a case–control study investigating the clinical phenotypes and genetic regulation of endometriosis in Indian women: the ECGRI study

**DOI:** 10.1136/bmjopen-2021-050844

**Published:** 2021-08-09

**Authors:** Rahul K. Gajbhiye, Grant Montgomery, Murlidhar V Pai, Pranay Phukan, Shashank Shekhar, Kedar Padte, Pramathes DasMahapatra, Bimal M. John, Chaitanya Shembekar, Aishwarya V. Bhurke, Nilajkumar Bagde, Ketki Kulkarni, Nagendra Sardeshpande, Anil Humane, Swati Mahobia, Millind Shah, Uma Singh, Aarti Srivastava, Gita Mishra, Neeta Warty, Sunita Chandra, Smita D. Mahale

**Affiliations:** 1 Clinical Research Lab, ICMR-National Institute for Research in Reproductive Health, Mumbai, Maharashtra, India; 2 Institute for Molecular Bioscience, The University of Queensland, St Lucia, QLD, Australia; 3 Department of Obstetrics and Gynaecology, Kasturba Medical College, Manipal, Karnataka, India; 4 Department of Obstetrics and Gynaecology, Assam Medical College, Dibrugarh, Assam, India; 5 Department of Obstetrics and Gynaecology, All India Institute of Medical Sciences, Jodhpur, Rajasthan, India; 6 Dr Kedar’s Maternity, Infertility, Surgical Hospital, Endoscopy and IVF Center, Panji, Goa, India; 7 Spectrum Clinic and Endoscopy Research Institute, Kolkata, West Bengal, India; 8 Minimally Invasive Surgery Unit, Credence Hospital, Thiruvananthapuram, Kerala, India; 9 Omega Hospital, Nagpur, Maharashtra, India; 10 Department of Obstetrics and Gynaecology, All India Institute of Medical Sciences, Raipur, Chhattisgarh, India; 11 Nowrosjee Wadia Maternity Hospital, Mumbai, Maharashtra, India; 12 Worli Hospital for Women, Mumbai, Maharashtra, India; 13 Department of Obstetrics and Gynaecology, Government Medical College, Nagpur, Maharashtra, India; 14 Sai Baba Nursing Home, Raipur, Chhattisgarh, India; 15 Naval Maternity Endoscopy & Infertility Center, Solapur, Maharashtra, India; 16 Department of Obstetrics and Gynaecology, King George's Medical University, Lucknow, Uttar Pradesh, India; 17 School of Public Health, The University of Queensland, Herston, QLD, Australia; 18 Sanjeevani Gynaecological & Endoscopy Centre, Mumbai, Maharashtra, India; 19 Morpheus Lucknow Fertility Center, Lucknow, Uttar Pradesh, India; 20 Emeritus Scientist, ICMR-National Institute for Research in Reproductive Health, Mumbai, Maharashtra, India

**Keywords:** epidemiology, genetics, gynaecology

## Abstract

**Introduction:**

Endometriosis is one of the common, gynaecological disorders associated with chronic pelvic pain and subfertility affecting ~10% of reproductive age women. The clinical presentation, etiopathogenesis of endometriosis subtypes and associated risk factors are largely unknown. Genome-Wide Association (GWA) Studies (GWAS) provide strong evidence for the role of genetic risk factors contributing to endometriosis. However, no studies have investigated the association of the GWAS-identified single-nucleotide polymorphism (SNPs) with endometriosis risk in the Indian population; therefore, one-sixth of the world’s population is not represented in the global genome consortiums on endometriosis. The Endometriosis Clinical and Genetic Research in India (ECGRI) study aims to broaden our understanding of the clinical phenotypes and genetic risks associated with endometriosis.

**Methods and analysis:**

ECGRI is a large-scale, multisite, case–control study of 2000 endometriosis cases and 2000 hospital controls to be recruited over 4 years at 15 collaborating study sites across India covering representative Indian population from east, north-east, north, central, west and southern geographical zones of India. We will use the World Endometriosis Research Foundation Endometriosis Phenome and Biobanking Harmonisation Project (WERF-EPHect) data collection instruments for capturing information on clinical, epidemiological, lifestyle, environmental and surgical factors. WERF-EPHect standard operating procedures will be followed for the collection, processing and storage of biological samples. The principal analyses will be for main outcome measures of the incidence of endometriosis, disease subtypes and disease severity determined from the clinical data. This will be followed by GWAS within and across ethnic groups.

**Ethics and dissemination:**

The study is approved by the Institutional Ethics Committee of Indian Council of Medical Research-National Institute for Research in Reproductive Health and all participating study sites. The study is also approved by the Health Ministry Screening Committee of the Government of India. The results from this study will be actively disseminated through discussions with endometriosis patient groups, conference presentations and published manuscripts.

**Strengths and limitations of this study**

- This multicentre study will identify the clinical, epidemiological, environmental and lifestyle risk factors associated with different endometriosis subtypes in Indian women.
- World Endometriosis Research Foundation Endometriosis Phenome and Biobanking Harmonisation Project standards are followed to ensure high standards of clinical data collection, sample processing and storage.
- The study extends the discovery of genetic and environmental risk factors for endometriosis to the Indian population adding significantly to our understanding of the causes of this disease.
- The study will lead to the establishment of a biorepository for endometriosis in India with associated detailed clinical and genomic data.
- The hospital controls comprise female visitors unrelated to the endometriosis cases with no personal or family history of endometriosis; thus, the endometriosis in the control group may not be completely ruled out.

## Introduction

Endometriosis is defined as the presence of endometrium-like tissue outside the normal uterine cavity.^1^ It is estimated to affect ∼10% of women of reproductive age in any population and extrapolates to 247 million affected women worldwide and 42 million women in India.^2^ India has a population size of more than 1.3 billion and comprises different ethnic and geographically distinct subpopulations.^3^ Women with endometriosis complain of having chronic pelvic pain, dysmenorrhoea, dyspareunia, dyschezia, dysuria, fatigue and infertility.^1^ The data on the distribution and clinical presentation of endometriosis in low income and middle income countries (LMICs) as well as low-resource settings are extremely limited.^4^ The aetiology and risk factors associated with endometriosis are not known despite many years of research. Many theories have been proposed such as retrograde menstruation, coelomic metaplasia, the combination of both called the induction theory, lymphatics and vascular metastasis and the most recent theory of epigenetics,^5 6^ genetics,^7–10^ autoimmunity^11 12^ and inflammation^13^ are reported to be of significance in the aetiology of endometriosis. There is evidence suggesting in utero exposure, menstrual and reproductive factors, anthropometrics, lifestyle, dietary and environmental exposure might play a role in the aetiology of endometriosis. However, out of these factors, only earlier age at menarche and shorter menstrual cycle length consistently showed an increased risk of endometriosis. Similarly, greater parity and current oral contraceptive use showed decreased risk of endometriosis consistently.^14 15^ However, there is no clear understanding of the aetiology and pathogenesis of endometriosis and therefore appropriate treatment of the disease is still a major clinical challenge.

The presentation of endometriosis varies from superficial peritoneal lesions of different colours (SUP) to cysts in the ovaries (endometrioma (OMA)) to nodules with a depth of penetration >5 mm (deeply infiltrating endometriosis (DIE), often associated with fibrosis and adhesions) to the lesions on extra pelvic locations.^1^ It is also argued that these subtypes of endometriosis might have different aetiologies^16^ and the endometriosis lesions may vary across the life course.^1^ There is also an overlap of these endometriosis subtypes in some women and sometimes all of these subtypes are present in the same endometriosis patient. However, there is limited information on the distribution of endometriosis subtypes and associated risks factors.

The heritability of endometriosis was estimated at ~50% based on Australian twin studies.^17^ Similar studies in the Swedish Twin registry reported 47% genetic factors and 53% unique environmental effects in women with endometriosis.^18^ Genome-Wide Association (GWA) Studies (GWAS) provide strong evidence for the role of genetic risk factors contributing to endometriosis.^4 9 19–21^ The interim results of the ongoing meta-analysis involving 25 global data sets identified 27 genome-wide significant loci,^22^ accounting for approximately 2.2% of disease variance for endometriosis.^1^ Seventy-eight per cent (21 loci) had greater effect sizes in stage III/IV disease compared with stage I/II and 4% (1 locus) had greater effect size in stage I/II compared with stage III/IV and 63% (17 loci) had greater effect sizes when restricted to endometriosis with infertility as compared with overall endometriosis cases.^22^ These observations emerging from the largest meta-analysis of endometriosis suggest that different subtypes of endometriosis might have specific risk variants.

Previously, we reported a family history of endometriosis with the diagnosis of the disease in either the mother, sister or grandmother of the proband, with some women reporting multiple affected relatives mainly from West Bengal or Assam regions of India.^23^ Additionally, we also observed the increased incidence of DIE in these regions. Endometriosis cases with unusual presentation such as endometriosis-associated endometrioid type of ovarian cancer, cervical endometriosis, endometriosis-associated with didelphys uterus were also reported in Indian women.^23^ Possible explanations for differences in incidence and disease presentation in different regions of India include differences in ethnicity and/or environmental factors. Therefore, there exists an unmet need to study distributions and presentation of endometriosis in Indian women and also characterise the different subtypes of endometriosis, SUP, OMA and DIE, and the presentation of the disease in different ethnic subpopulations in India. To date, no studies have investigated the association of the GWAS-identified SNPs with endometriosis risk in the Indian population, therefore, leaving one-sixth of the world population not represented in the International Endometriosis Genome Consortium (IEGC).

To achieve these goals, we are collecting prospective high-quality, standardised clinical, epidemiological, environmental and surgical data with corresponding biological samples. Our group has been actively engaged with World Endometriosis Research Foundation’s (WERF) Endometriosis Phenome and Biobanking Harmonisation Project (EPHect) for implementing the WERF-EPHect research instruments and also translating the Endometriosis Patient Questionnaires (EPQ) in different Indian languages.

The Endometriosis Clinical and Genetic Research in India (ECGRI) study aims to conduct a large-scale, prospective, case–control study to investigate the clinical phenotypes and genetic risks associated with endometriosis in the Indian population.

### Objectives

1. To identify and characterise endometriosis subtypes [superficial peritoneal endometriosis (SUP), cystic ovarian endometriosis or OMA, and DIE] in different geographical regions of India.
2. To identify the clinical, epidemiological, environmental, lifestyle risk factors associated with different endometriosis subtypes in India.
3. To investigate the genetic risks associated with endometriosis in the Indian population.

## Methods and analysis

### Study design and study sites

**Figure 1:**
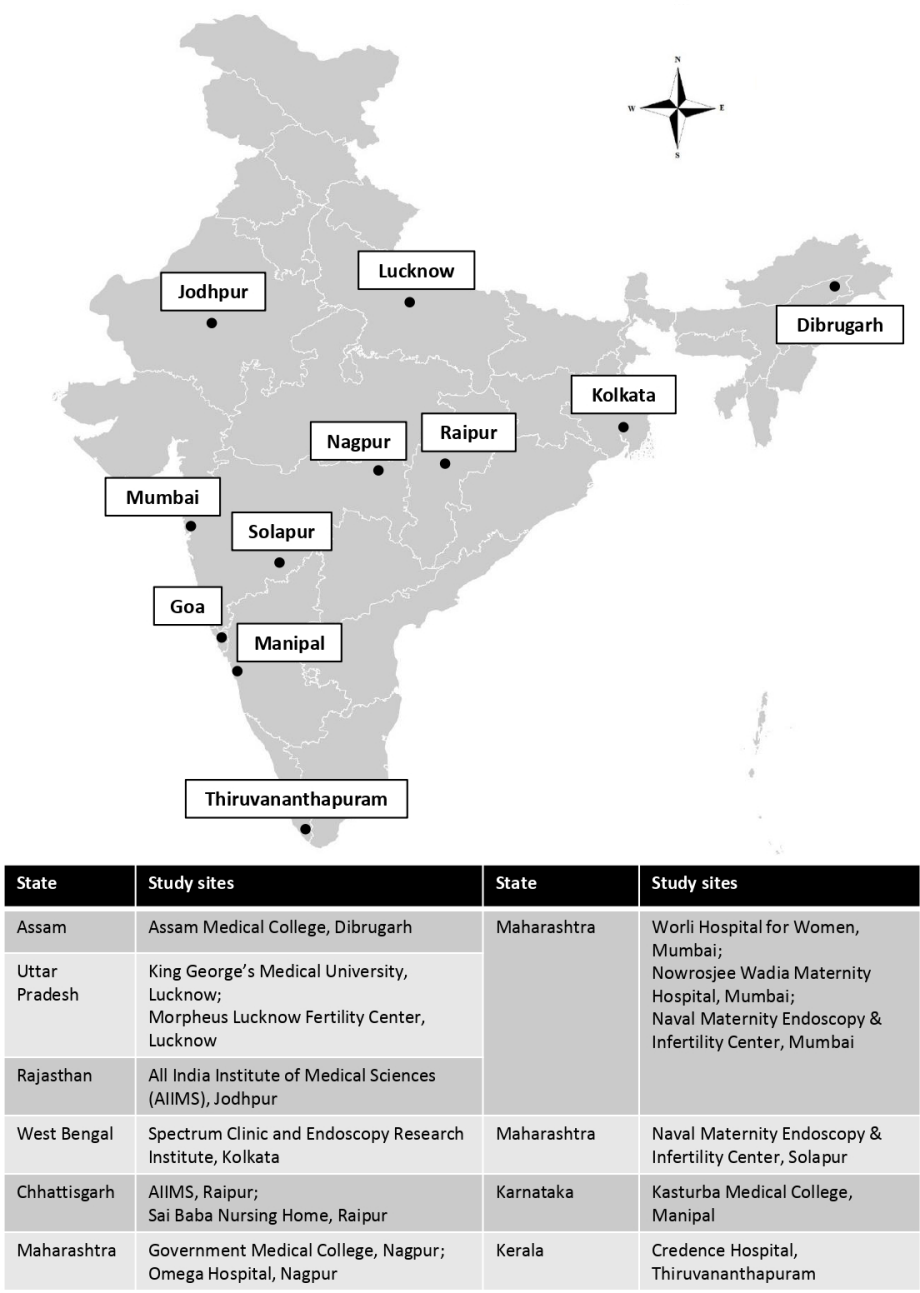
Study sites of ECGRI across different geographical regions of India.(ECGRI-Endometriosis Clinical and Genetic Research in India).

This is a multisite, prospective, case–control study conducted over 5 years beginning in October 2019 and ending in September 2024. It is being conducted at 15 collaborating study sites across India covering representative Indian populations from the north, northeast, east, central, west and south geographical zones of India (figure 1). The selection criteria of the study sites were as follows: (1) proven track record of gynaecologic research; (2) availability of required research manpower and infrastructure to be able to recruit the required study participants and (3) existing trusted relationship from previous collaborations. Each of these geographical zones where study participants will be recruited has significant genetic diversity which is likely to improve the ability of this research to answer the objectives of the study.

### Patient and public involvement

The ECGRI study was conceptualised based on our experience and feedback we received from collaborators and patients during our previous studies.^11 12^ The initial cohort of women with endometriosis provided feedback on general workflow, data collection and WERF-EPHect questionnaires.

### Samples

After obtaining written informed consent, 15 mL of peripheral blood will be collected by venepuncture by a trained phlebotomist (10 mL in EDTA tube and 5 mL in plain serum tube) from endometriosis cases and controls.

### Participants

We plan to recruit up to 2000 women with endometriosis and 2000 hospitals controls. The study participants will be recruited consecutively for 4 years at all the study sites until the desired sample size of 400 endometriosis cases and 400 controls per geographical zone is achieved.

### Inclusion criteria

#### Inclusion criteria for endometriosis cases

- Females aged 18–50 years, visiting clinics at the study sites for benign gynaecological conditions, will be initially screened and evaluated for their eligibility in the study.
- They should be willing to participate in the study, should be a resident of the geographical region of the collaborating centre for a minimum period of 1 year.
- Eligible females with a surgically confirmed diagnosis of endometriosis on laparoscopy will be recruited as endometriosis cases. Endometriosis will be further confirmed on histology wherever possible.
- The laparoscopic images of the endometriosis lesions will be provided by the clinical collaborators along with the completed WERF-EPHect Standard Surgical Form (SSF).
- Endometriosis cases will be subclassified as superficial peritoneal endometriosis (SUP), cystic ovarian endometriosis or OMA, and DIE. Endometriosis cases with any additional subtypes/phenotypic features other than SUP, OMA and DIE will also be included.
- Women with a family history of endometriosis will also be invited to participate with one proband per family included in the main study. Affected and non-affected relatives of the proband up to a minimum of three generations will be recruited wherever possible. Data of familial endometriosis cases will be analysed separately from the sporadic endometriosis cases.

#### Inclusion criteria for hospital controls

- The appropriate comparison group having the same opportunity to develop endometriosis and representative of the same geographical location as endometriosis cases will be selected from all the collaborating centres.
- These study participants will be the hospital-based controls residing in the geographical region of the collaborating centres for a minimum of 1 year.
- Female visitors aged 18–50 years, unrelated to the endometriosis cases, who are visiting the collaborating centres during the same period of recruitment of endometriosis cases will be eligible for enrolment as controls.

### Exclusion criteria

- Female visitors having personal or family history of endometriosis will be excluded from the control group.

### Participant enrolment

#### Screening of cases and controls

**Figure 2:**
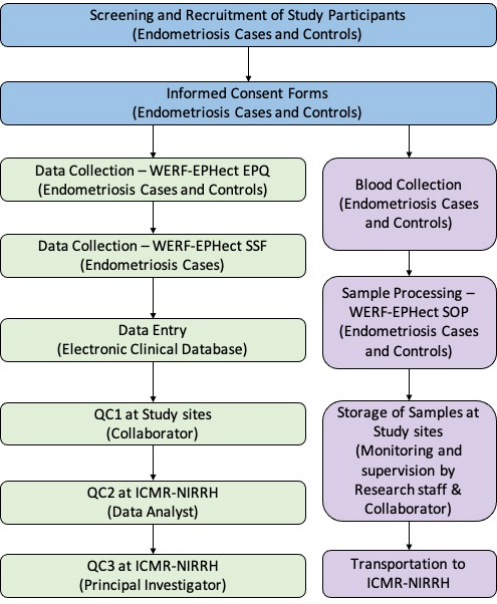
Flow chart of ECGRI study participant recruitment and study processes.ECGRI-Endometriosis Clinical and Genetic Research in India; EPQ- Endometriosis Patient Questionnaire; ICMR-NIRRH- Indian Council of Medical Research-National Institute for Research in Reproductive Health; QC- quality control; SOP- standard operating procedure; SSF-standard surgical form; WERF-EPHect- World Endometriosis Research Foundation Endometriosis Phenome and Biobanking Harmonisation Project.

The flow chart of study participant recruitment and study processes is represented in figure 2. Eligible study participants (endometriosis cases and hospital controls) will be screened and identified by the clinical collaborators at each study site. Clinical collaborators are experienced gynaecological laparoscopic surgeons having expertise in the diagnosis and management of endometriosis.

### Informed consent

Research staff who have received study-specific training will approach the study participants identified by the clinical collaborator. They will provide a participant information sheet (PIS) that includes the purpose and procedures of the study and carefully explain the same to the participants in their local language. They will explain to participants that participation in this study is entirely voluntary and participants are free to withdraw from the study at any point without affecting their medical care and services. A copy of the signed informed consent and PIS will be given to participants in the language they read and understand. Languages include Hindi, Marathi, Malayali, Assamese, Kannada, Bengali and English. The original signed consent form will be retained at the study site.

### Interventions

#### Non-clinical interventions

##### WERF-EPHect EPQ (modified)

To determine the differences in endometriosis subtypes, presentation and disease severity between geographical regions and ethnic groups in India, data will be collected with the WERF-EPHect EPQ modified version form.^24^ After obtaining the informed consent from the participants, the research staff will administer the modified WERF-EPHect EPQ under the supervision of clinical collaborators for both cases and controls. The modified EPQ is designed to capture phenotype data across the life cycle particularly relevant due to delay in diagnosis. Pain-related questionnaires on an 11-point Numerical Rating Scale (0–10) will capture four separate domains of pain (continuous, intermittent, neuropathic and affective). Information on depression, anxiety and health-related quality of life will be captured. Menstrual history including age at menarche, menstrual irregularities, last date of menstrual period, frequency, duration of flow and menstrual cycle length will be recorded. Use of hormonal treatments, length of time and type of treatment will be obtained. Information on subfertility, age at the start of each pregnancy, type of fertility treatment used for each pregnancy, and pregnancy outcome (if applicable), live births, type of delivery and pregnancy complications will be captured. A detailed history of medical and surgical treatments will also be obtained. The sociodemographic, personal and lifestyle data including age, ethnicity, education, anthropometric measurements, history of smoking, alcohol use and exercise will be captured by modified EPQ. Information on occupation and history of residence in each decade of life will also be captured (online supplemental table 1).

10.1136/bmjopen-2021-050844.supp1 Supplementary data

#### Standard surgical form

The surgical data will be collected by the clinical collaborators using WERF-EPHect SSF.^25^ EPHect SSF consists of two parts designed to capture all relevant visual information of the endometriosis lesion phenotype and the surgical treatment including type, duration of the procedure and requires approximately 1–3 min to complete the SSF. The first part of SSF includes detailed information on clinical covariates and requires detailed information related to the current menstrual cycle, current hormonal treatment, history of previous surgery for endometriosis, ultrasound or MRI findings before the procedure. The second part focuses on detailed visual information on intraoperative findings such as extent, exact location and colour of endometriotic lesions, with a specific focus on endometrioma dimensions and endometriotic nodules. It also includes information on the endometriosis fertility index. Based on the EPHect SSF data, the phenotype characterisation of endometriosis will be carried out into superficial peritoneal endometriosis (SUP), cystic ovarian endometriosis or OMA, and DIE. From the EPHect SSF data, rAFS (Revised American Fertility Society) score will be calculated and endometriosis cases will be grouped into stage I/II or stage III/IV.

#### Familial endometriosis

The clinical data of female relatives of the proband will be collected as per the WERF-EPHect and the family history of the proband will be documented through pedigree analysis. This will include both affected and non-affected relatives of the proband up to a minimum of three generations wherever possible.

#### Laparoscopic images documentation

The collaborators (gynaecological laparoscopic surgeons) at each study site will obtain laparoscopic images of the different endometriotic lesions and adhesions. The soft copies of the labelled laparoscopic images will be uploaded to the ECGRI electronic database.

### Power calculations

Over 4 years, the study team will recruit a total of 2000 endometriosis cases and 2000 hospital controls across five geographical zones of India. To detect an OR of ≥2 with a 95% CI and 90% power with 10% missing or incomplete WERF-EPHect data collection forms, the required sample size per zone=360. In each geographical zone, 400 endometriosis cases and 400 hospital controls will be recruited.

To replicate results from the European GWAS^20 22^ (p=0.05/27 or p<0.0018) and test all 27 ‘hits’ using either average effect size or effect size for the smallest effect (Risk allele with frequency 0.3 and genotype relative risk 1.22 with 80% power), the total sample size required is as follows: endometriosis cases (n=1250), hospital controls (n=1250). For conducting discovery GWAS in the Indian population (effect size to detect at p<5×10^−8^), a total sample size of 2000 endometriosis and 2000 hospital controls will be recruited. To conduct replication GWAS for novel risk loci in Indian women with endometriosis an independent set of endometriosis cases and controls will be recruited based on discovery GWAS results. The sample size of the present study is calculated taking into consideration the expected effect sizes for genetic risk factors, the possibility for discovery of population-specific risk factors, selection criteria for cases and controls, and differences in allelic frequencies among studied populations.^26^

### Electronic database

A dedicated website is developed for the project (https://ecgri.in). Electronic data collection software is developed for online data entry (http://app.ecgri.in/login). The project assistants will enter the data electronically from the paper copies of the WERF-EPHect EPQ and SSF. The online electronic data collection software (http://app.ecgri.in/login) is hosted on a secure server at the Biomedical Informatics Division of Indian Council of Medical Research (ICMR)-National Institute for Research in Reproductive Health (NIRRH), Mumbai. The central database will allow online, real-time data entry with the option of online editing. Project assistants and site investigators will have access to data entry, data editing only for their respective centres and will not have any access to the whole database.

### Research team training and quality controls

The research staff has received study-specific training including recruitment of study participants, obtaining informed consent, counselling the study participants, data collection as per WERF-EPHect EPQ, sample collection, processing, storage and transport as per WERF-EPHect standard operating procedures (SOPs). Intense training was provided to the research staff, clinical collaborators on selection and recruitment of study participants, data collection as per WERF-EPHect Questionnaire and SSF, sample collection, processing and transportation as per WERF-EPHect SOPs and online data entry. A copy of the training manual is provided to all study sites. A data analyst will coordinate with clinical collaborators and research staff for timely entry of the data and transportation of samples. The data analyst will report to RG at ICMR-NIRRH.

RD will provide training to the data analyst on data collection, quality control, data entry and online editing, data analysis, development and maintenance of databases at ICMR-NIRRH. The research staff will report to their respective clinical collaborators at participating study sites. The data analyst will supervise, conduct regular quality control checks and clean the data to identify and rectify the errors by comparing the hard and soft copies of data collection instruments. RG will coordinate with clinical collaborators for recruitment of study participants, data collection, data entry, blood sample collection processing and transport as per the WERF-EPHect research instruments. Video meetings will be organised with all collaborating centres, a research team at ICMR-NIRRH to review the progress and ensure adherence to protocol. A detailed checklist is provided to the collaborating centres to ensure adherence to the protocol. There will be weekly auditing of data collection and data entry for each site by the clinical collaborator to ensure that the protocols are followed and data entry is done promptly as soon as the data are collected. The data analyst will coordinate with the collaborating centres every week through email/video/teleconference. There will be detailed regular monitoring of a random sample of individuals collected at each site. The centralised database at NIRRH, Mumbai will be checked and updated every week to provide regular reports from each study site and across the study on the number of cases and controls collected with additional information on the completion of all parts of the protocol for each individual. RG will provide regular feedback to each and all sites on issues of compliance every month. The hard copies of the original documents (WERF-EPHect instruments-modified EPQ, SSF) will be transported to the ICMR-NIRRH for cross-checking the data entry. These data forms will be stored as per the National Ethical Guidelines for Biomedical and Health Research Involving Human Participants.^27^

### Collection, processing and storage of blood samples

The SOPs have been established and are being implemented in RG’s laboratory as per the WERF-EPHect collection protocol.^28^ The cases and controls will be recruited simultaneously during the study period. After obtaining informed consent, 15 mL peripheral blood (10 mL in EDTA tube and 5 mL in plain serum tube) will be collected in an appropriate setting. The resulting plasma/serum will be stored in aliquots (500 µL) prior to freezing in −80°C freezer with power back up. Collected samples will be separated into its three derivatives (serum, plasma and buffy coat). All the details of the collection will be recorded on the EPHect Biospecimens form.

### Sample processing, genotyping and quality control

Genomic DNA will be extracted from buffy coats using the QIAamp DNA Blood Midi kit (Qiagen) using the manufacturer’s instructions. The concentration of each DNA sample will be determined. The DNA samples that pass minimum requirements for sample quality will be sent to the commercial facility for genome-wide genotype analysis using the appropriate genotyping platform (Illumina/Afffimatrix) specific to Asian Indian populations. Standard genotype quality control procedures will be performed using the software PLINK. SNPs with a high missing rate (>5%), low minor allele frequency (<1×10^−4^) and with Hardy-Weinberg equilibrium p<10–6 will be excluded along with samples with low call rates (<95%), extreme heterozygosity, high genetic relatedness and any ancestry outliers. Standard methods for quality control and genetic associations will be adopted as reported earlier.^20^ Following quality control, genotypes will be imputed to the 1000 genomes phase 3 reference panel using the Michigan Imputation Server. Endometriosis cases with a family history either in grandmother, mother or sister will be recruited for enrichment sampling. The genotype data of familial endometriosis cases will be analysed separately. Exome sequencing will be carried out for the identification of the genes associated with familial endometriosis.

### Outcomes

#### Primary outcome

We will use the modified WERF-EPQ and Surgical Standard Form (SSF) data to data collection investigate the clinical, epidemiological, and environmental, and lifestyle risk factors in women with endometriosis. The GWAS analysis will be carried out to investigate the genetic risk factors in women with endometriosis. At the end of the study participant’s recruitment period, that is, from August 2023 onwards, the collected data and samples will be analysed and compared between women with endometriosis and hospital controls recruited at different clinics and different regions of India. The clinical and phenotypic differences in the presentation of endometriosis at different clinics and different regions of India will be determined. The replication in the Indian population of genetic risk factors for endometriosis discovered in European populations will be conducted.

#### Secondary outcomes

The correlation of clinical and genomic data together with endometriosis subtype status will allow us to discover and replicate genetic risk loci for endometriosis subtypes and help in understanding the pathogenesis of endometriosis and its subtypes. The clinical and genetic findings will be compared against the public databases and functional studies will be carried out in an appropriate model.

Secondary outcomes will be a possible discovery of population-specific common risk factors for endometriosis and if there are significant differences in the clinical presentation of endometriosis in different regions of India. We will conduct secondary analyses, making use of the genetic information to define ethnic subgroups across India, to evaluate whether these differences might result from ethnic or environmental differences between regions.

This study will also provide an opportunity to add the endometriosis phenotype–genotype data to the International Consortium. Regional data on endometriosis subphenotypes, demographic, environmental, reproductive, menstrual, lifestyle factors of endometriosis will be useful in developing the strategies and policies to address the endometriosis-associated morbidity in India and LMICs.

### Data analysis plan

We will use SPSS (version no. 26), Graph Pad Prism, R and plink software for analysis. The principal analyses will be for main outcome measures of the incidence of endometriosis, disease subtypes and disease severity determined from the clinical data. We will conduct conditional logistic regression to analyse effects of geographical region and ethnicity (defined from genetic data) adjusting for age, recruitment clinic and other confounding variables that may be identified from the initial inspection of the data. Disease presentation and endometriosis severity between regions and ethnic groups in India will be analysed. Comorbidity and environmental exposures between regions will be compared and correlated with disease severity and distribution of lesions. Genotype data will be used to define ethnicity for the phenotypic analysis. Association between previously identified endometriosis risk loci and endometriosis status in our Indian cohort will be analysed on imputed genotypes using SNPTEST V.2. An additive logistic regression model will be used to test association while controlling for population stratification by including age and the top five principle components as covariates in the model. A study-wide significance threshold of p<0.05/n will be applied where n is the number of loci tested. Subsequently, we will perform a GWA analysis using SNPTEST to estimate adjusted ORs and corresponding 95% CI between each available SNP and endometriosis case/control status. The subphenotype association analysis will be performed for rAFS stage, site, geographical regions (North, East, North East, South, West and Central India), infertility and associated comorbidities (reproductive, metabolic, inflammatory, autoimmune disorders and COVID-19, etc).

Functional studies aimed at delineating the causal genetic variants and biological mechanisms underlying the observed statistical associations with endometriosis risk will be carried based on the results of the discovery GWAS. Meta-analysis in collaboration with IEGC and other genomic groups will be explored.

### Plans for data management, curation, storage and confidentiality

Clinical and genomic datasets from Indian women with and without endometriosis will be captured in a central database (http://app.ecgri.in/login), maintained and regularly updated at ICMR-NIRRH. The database is developed on a robust platform including the ability to allow online, real-time data entry. Demographic data will be kept in separate tables and records identifying the study participants will be kept confidential. Data entry and data access will be independently controlled and identifiable data held behind secure firewalls according to the ICMR-National Guidelines for Biomedical and Health Research Involving Human Participants.^27^ Data collected from different sites will be held separately and only specific individuals within a research team will be able to access and edit data. Where possible, data will be captured using online tools, including the clinical data in the WERF-EPHect instruments. Data collected from paper questionnaires will be entered and verified independently. The quality of the clinical and genomic data will be regularly monitored. The genotype data will be stored in a separate computer with sufficient memory space and backup data will be stored in hard disks. The clinical and genomic data will be carefully linked by individual identification numbers to maintain the confidentiality of the participants and the key will be held by RG at ICMR-NIRRH.

### Ethics and dissemination

The study has ethics approval and will be monitored by the Institutional Ethics Committee (IEC) of the study sites and ICMR-NIRRH. The study protocol will also be monitored by the Health Ministry Screening Committee (HMSC) of the government of India. Periodic progress reports are required to be submitted to HMSC, IEC of ICMR-NIRRH and IECs of study sites. A periodic progress report will be submitted to the funding agency.

## Supplementary Material

Reviewer comments

Author's manuscript
