# Supplementary material for: Protocol for a case–control study investigating the clinical phenotypes and genetic regulation of endometriosis in Indian women: the ECGRI study
Source: BMJ Open. 2021 Aug 9;11(8):e050844. doi: 10.1136/bmjopen-2021-050844 (PMC8354274; doi:10.1136/bmjopen-2021-050844)
Supplement: Supplementary data [file bmjopen-2021-050844supp001.pdf]

**Supplementary Table 1:** Clinical, epidemiological, reproductive, lifestyle and environmental characteristics captured using modified Endometriosis Patient Questionnaire (EPQ)

| Characteristics | Variables |
|---|---|
| **Menstrual and Reproductive factors** | Age at menarche, menstrual cycle length, menstrual volume, dysmenorrhea, current oral contraceptive use, hormones, history of infertility |
| **Pregnancy and Fertility** | Age at each pregnancy, parity, fertility treatment, outcome of pregnancy, history of miscarriage, ectopic pregnancy, vaginal delivery, caesarean section, breast feeding, natural pregnancy/ ART, complications related to pregnancy- GDM, PIH, Preterm birth |
| **Health-related Quality of life** | Thoughts and feelings associated with pain, severity grading scale (0-10), Life time details - pelvic pain, pain during or after vaginal intercourse, treatment for pain, gastrointestinal and urinary symptoms etc. |
| **Socio-demographic, personal and lifestyle factors** | Geographic location, age, educational level, marital status, age at marriage, employment, family income, height, weight, BMI, waist -to- hip ratio, ethnicity, cigarette smoking, alcohol use, caffeine intake, regular exercise, night shift work, natural hair color, eye color, skin color, body shape |
| **Medical and surgical** | History of associated diseases- cancer, autoimmune diseases<br>endometriosis or chronic pelvic pain history- personal and family (if known), details of medical and surgical treatment |
| **Environmental factors** | Residence in each decade of life, pollution exposure, smoking environment,<br>sun exposure, occupational history, pesticide exposure, any other relevant information |

Abbreviations: ART, Assisted Reproductive Technology; GDM, Gestational Diabetes Mellitus; PIH, Pregnancy-induced hypertension; BMI, Body mass index
