# Supplementary material for: Protocol for a case–control study investigating the clinical phenotypes and genetic regulation of endometriosis in Indian women: the ECGRI study
Source: BMJ Open. 2021 Aug 9;11(8):e050844. doi: 10.1136/bmjopen-2021-050844 (PMC8354274; doi:10.1136/bmjopen-2021-050844)
Supplement: Author's manuscript [file bmjopen-2021-050844.draft_revisions.pdf]

BMJ Open is committed to open peer review. As part of this commitment we make the peer review history of every article we publish publicly available.

When an article is published we post the peer reviewers' comments and the authors' responses online. We also post the versions of the paper that were used during peer review. These are the versions that the peer review comments apply to.

The versions of the paper that follow are the versions that were submitted during the peer review process. They are not the versions of record or the final published versions. They should not be cited or distributed as the published version of this manuscript.

BMJ Open is an open access journal and the full, final, typeset and author-corrected version of record of the manuscript is available on our site with no access controls, subscription charges or pay-per-view fees (http://bmjopen.bmj.com).

If you have any questions on BMJ Open's open peer review process please email info.bmjopen@bmj.com

Protocol for a case-control study investigating the clinical phenotypes and genetic regulation of endometriosis in Indian women: the ECGRI study

Journal:	*BMJ Open*
Manuscript ID	bmjopen-2021-050844
Article Type:	Protocol
Date Submitted by the Author:	02-Mar-2021
Complete List of Authors:	Gajbhiye, Rahul; NIRRH, ;<br>Montgomery, Grant ; The University of Queensland Institute for Molecular Bioscience<br>Pai, Murlidhar; Kasturba Medical College Manipal<br>Phukan, Pranay; Assam Medical College<br>Shekhar, Shahank; All India Institute of Medical Sciences Jodphur<br>Padte , Kedar; 6 Dr Kedar's Maternity, Infertility and Surgical Hospital, Endoscopy and IVF Center, Panaji, India<br>Dasmahapatra , Pramathes; Spectrum Clinic and Endoscopy Research Institute, Kolkata, India<br>John, Bimal; Credence Hospital, Thiruvananthapuram, India<br>Shembekar, Chaitanya ; Omega Hospital, Nagpur, India<br>Bhurke , Aishwarya<br>Bagde, Nilajkumar; All India Institute of Medical Sciences - Raipur<br>Kulkarni, Ketki; Nowrosjee Wadia Maternity Hospital<br>Sardeshpande , Nagendra; Worli Hospital for Women, Mumbai, India<br>Humne, Anil; Government Medical College and Hospital Nagpur<br>Mahobia , Swati; Sai Baba Nursing Home, Raipur, India<br>Shah, Millind; Naval Maternity Endoscopy & Infertility Center, Solapur, India<br>Singh, Uma; King George Medical College<br>Shrivastav, Arti; King George Medical College<br>Mishra, Gita; University of Queensland, Faculty of Medicine, School of Public Health<br>Warty, Neeta; Sanjeevani Diagnostic Center, Mumbai, India<br>Chandra, Sunita; Morpheus Lucknow Fertility Center, Lucknow, India<br>mahale , smita
Keywords:	EPIDEMIOLOGY, GENETICS, GYNAECOLOGY

SCHOLARONE™
Manuscripts

I, the Submitting Author has the right to grant and does grant on behalf of all authors of the Work (as defined in the below author licence), an exclusive licence and/or a non-exclusive licence for contributions from authors who are: i) UK Crown employees; ii) where BMJ has agreed a CC-BY licence shall apply, and/or iii) in accordance with the terms applicable for US Federal Government officers or employees acting as part of their official duties; on a worldwide, perpetual, irrevocable, royalty-free basis to BMJ Publishing Group Ltd ("BMJ") its licensees and where the relevant Journal is co-owned by BMJ to the co-owners of the Journal, to publish the Work in this journal and any other BMJ products and to exploit all rights, as set out in our licence.

The Submitting Author accepts and understands that any supply made under these terms is made by BMJ to the Submitting Author unless you are acting as an employee on behalf of your employer or a postgraduate student of an affiliated institution which is paying any applicable article publishing charge ("APC") for Open Access articles. Where the Submitting Author wishes to make the Work available on an Open Access basis (and intends to pay the relevant APC), the terms of reuse of such Open Access shall be governed by a Creative Commons licence – details of these licences and which Creative Commons licence will apply to this Work are set out in our licence referred to above.

Other than as permitted in any relevant BMJ Author's Self Archiving Policies, I confirm this Work has not been accepted for publication elsewhere, is not being considered for publication elsewhere and does not duplicate material already published. I confirm all authors consent to publication of this Work and authorise the granting of this licence.

For peer review only

Title: Protocol for a case-control study investigating the clinical phenotypes and genetic regulation of endometriosis in Indian women: the ECGRI study

Authors

Rahul K Gajbhiye, MBBS, Ph.D.[1]* (0000-0003-3864-1818),

Grant W Montgomery, Ph.D.[2] (0000-0002-4140-8139),

Muralidhar Pai, M.D.[3] (0000-0002-7942-2383),

Pranay Phukan, M.D.[4],

Shashank Shekhar, M.D.[5] (0000-0003-4612-6533),

Kedar Padte, M.D.[6],

Pramathes DasMahapatra, M.D.[7],

Bimal John, M.D.[8] (0000-0003-4530-2611),

Chaitanya Shembekar, M.D.[9],

Aishwarya V Bhurke, M.Sc.[1] (0000-0002-2000-2076),

Nilajkumar Bagde, M.D.[10] (0000-0003-3035-9906),

Ketki Kulkarni, M.D.[11],

Nagendra Sardeshpande, D.N.B.[12],

Anil Humane, MBBS, D.N.B[13],

Swati Mahobia, M.D[14],

Milind Shah, M.D[15] (0000-0002-5277-0977),

Uma Singh, M.S.[16],

Aarti Srivastava, D.G.O. [16],

Gita D Mishra, Ph.D.[17](0000-0001-9610-5904),

Neeta Warty, M.D.[18],

Sunita Chandra, M.D.[19],

Smita D Mahale, Ph.D [20#] (0000-0002-4164-7455).

For peer review only

For peer review only - http://bmjopen.bmj.com/site/about/guidelines.xhtml

Author Affiliations

[1]Indian Council of Medical Research (ICMR)-National Institute for Research in Reproductive Health (NIRRH), Mumbai, India

[2]Institute for Molecular Biosciences, The University of Queensland, St Lucia, Queensland, Australia

[3]Department of Obstetrics and Gynecology, Kasturba Medical College, Manipal, India

[4]Department of Obstetrics and Gynecology, Assam Medical College, Dibrugarh, India

[5]Department of Obstetrics and Gynecology, All India Institute of Medical Sciences, Jodhpur, India

[6]Dr Kedar's Maternity, Infertility and Surgical Hospital, Endoscopy and IVF Center, Panaji, India

[7]Spectrum Clinic and Endoscopy Research Institute, Kolkata, India

[8]Minimally Invasive Surgery unit, Credence Hospital, Thiruvananthapuram, India

[9]Omega Hospital, Nagpur, India

[10]Department of Obstetrics and Gynecology, All India Institute of Medical Sciences, Raipur, India

[11]Nowrosjee Wadia Maternity Hospital, Mumbai, India

[12]Worli Hospital for Women, Mumbai, India

[13] Government Medical College, Nagpur, India

[14]Sai Baba Nursing Home, Raipur, India

[15]Naval Maternity Endoscopy & Infertility Center, Solapur, India

[16]King George's Medical University, Lucknow, India

[17]Epidemiology and Biostatistics Division, School of Public Health, The University of Queensland, Herston, Queensland, Australia

[18]Sanjeevani Diagnostic Center, Mumbai, India

For peer review only

[19] Morpheus Lucknow Fertility Center, Lucknow, India

[20] Structural Biology and Biomedical Informatics Division, ICMR-NIRRH, Mumbai, India

[#] *Superannuated*

[*] **Corresponding author:** Rahul Gajbhiye

Scientist D & DBT Wellcome India Alliance Clinical & Public Health Intermediate Fellow

ICMR-National Institute for Research in Reproductive Health,

J. M. Street, Parel, Mumbai, 400012, India.

Tel: +91-22-24192036

Email: gajbhiyer@nirrh.res.in

Word count: 4333

For peer review only - http://bmjopen.bmj.com/site/about/guidelines.xhtml

Abstract

Introduction

Endometriosis is one of the common, gynaecological disorders associated with chronic pelvic pain and subfertility leading to significant morbidity affecting ~10% of reproductive age women in any population. The presentation and severity of endometriosis subtypes is unknown in Indian women. The etio-pathogenesis of endometriosis subtypes and associated risk factors are also largely unknown. Genome-wide association (GWA) studies provide strong evidence for the role of genetic risk factors contributing to endometriosis. However, no studies have investigated the association of the GWAS-identified SNPs with endometriosis risk in the Indian population; therefore, one sixth of the world's population is not represented in the global genome consortiums on endometriosis. The Endometriosis Clinical and Genetic Research in India (ECGRI) study aims to broaden our understanding of the clinical phenotypes and genetic risks associated with endometriosis in the Indian population.

Methods and analysis ECGRI is a large scale, multi-site, case-control study of 2000 endometriosis cases and 2000 hospital controls to be recruited over a 4 year period at 15 collaborating study sites across India covering representative Indian population covering east/north east, north, central, west and southern zones of India. We will utilize the World Endometriosis Research Foundation Endometriosis Phenome and Biobanking Harmonisation Project (WERF-EPHect) data collection instruments for capturing information on clinical, epidemiological, life style, environmental, and surgical factors. WERF-EPHect standard operating procedures will be followed for collection, processing and storage of biological samples. The principal analyses will be for main outcome measures of the incidence of endometriosis, disease subtypes and disease severity determined from the clinical data. This will be followed by GWAS within and across ethnic groups.

For peer review only - http://bmjopen.bmj.com/site/about/guidelines.xhtml

Ethics and dissemination: The study is approved by the Institutional Ethics Committee of ICMR-National Institute for Research in Reproductive Health (NIRRH) and all participating study sites. The study is also approved by Health Ministry Screening Committee of Government of India.

Strengths and limitations of the study

- ECGRI is the first GWAS on endometriosis from low middle income countries (LMICs) and India.
- This is the first large scale study to investigate endometriosis sub-phenotypes and associated risk factors in Indian women.
- Significant phenotypic data will be collected using the globally standardized and validated instruments of WERF-EPHect.
- The study will lead to establishment of endometriosis bio-repository of serum, plasma, DNA samples as well as establishment of clinical, genomic database of endometriosis in India.
- The hospital controls comprise female visitors aged 18-50 years, unrelated to the endometriosis cases with no personal or family history of endometriosis; thus, the endometriosis in the control group may not be completely ruled out.

For peer review only - http://bmjopen.bmj.com/site/about/guidelines.xhtml

Introduction

Endometriosis is defined as the presence of endometrium like tissue outside the normal uterine cavity [1]. It is estimated to affect 10% of women of reproductive age in any population and extrapolates to approximately 247 million affected women worldwide and 42 million women in India [2]. India has a population size of more than 1.3 billion and comprises different ethnic and geographically distinct sub-populations [3]. Women with endometriosis complain of having chronic pelvic pain, dysmenorrhea, dyspareunia, dyschezia, dysuria, fatigue and infertility [1]. The data on distribution and clinical presentation of endometriosis in LMICs as well as low-resource settings is extremely limited [4]. The etiology and risk factors associated with endometriosis are not clearly known in spite of many years of research. Many theories have been proposed such as retrograde menstruation, coelomic metaplasia, the combination of both called the induction theory, lymphatics and vascular metastasis, and the most recent theory of Epigenetics [5,6], genetics [7–10], autoimmunity [11,12], and inflammation [13] are reported to be of significance in the etiology of endometriosis. There is evidence suggesting in utero exposure, menstrual and reproductive factors, anthropometrics, lifestyle, dietary and environmental exposure might play a role in etiology of endometriosis. However, out of these factors only earlier age at menarche and shorter menstrual cycle length consistently show increased risk of endometriosis. Similarly, greater parity and current oral contraceptive use showed decreased risk of endometriosis consistently [14,15]. However, there is no clear understanding on the etiology and pathogenesis and therefore appropriate treatment of the disease is still a major clinical challenge.

The presentation of endometriosis varies from superficial peritoneal lesions of different colours (SUP) to cysts in the ovaries [endometrioma (OMA)], to nodules with depth of penetration > 5 mm [deeply infiltrating endometriosis (DIE), often associated with fibrosis

For peer review only - http://bmjopen.bmj.com/site/about/guidelines.xhtml

and adhesions] to the lesions on extra pelvic locations [16]. It is also argued that these subtypes of endometriosis might have different aetiologies [17] and the endometriosis lesions may vary across the life course [16]. There is also overlap of these endometriosis subtypes in some women and sometimes all of these subtypes are present in the same endometriosis patient. However, there is limited information on distribution of endometriosis subtypes and associated risks factors.

The heritability of endometriosis was estimated at ~50% based on Australian twin studies [18]. Similar studies in the Swedish Twin registry reported 47% genetic factors and 53% unique environmental effects in women with endometriosis [19]. Genome-wide association (GWA) studies provide strong evidence for the role of genetic risk factors contributing to endometriosis [4,9,20–22]. The interim results of the ongoing meta-analysis involving 25 global data sets identified 27 genome wide significant loci [23], accounting for approximately 2.2% of disease variance for endometriosis [16]. Seventy-eight percent (21 loci) had greater effect sizes in stage III/IV disease compared to stage I/II and 4% (1 locus) had greater effect size in stage I/II compared to stage III/IV and 63% (17 loci) had greater effect sizes when restricted to endometriosis with infertility as compared to overall endometriosis cases [23]. These observations emerging from largest meta-analysis of endometriosis suggests that different subtypes of endometriosis might have specific risk variants.

Previously, we reported a family history of endometriosis with diagnosis of the disease in either the mother, sister or grandmother of the proband, with some women reporting multiple affected relatives mainly from West Bengal or Assam regions of India [24]. Additionally, we also observed the increased incidence of deep infiltrating endometriosis in these regions. Endometriosis cases with unusual presentation such as endometriosis associated endometrioid type of ovarian cancer, cervical endometriosis, endometriosis associated with didelphys uterus were also reported in Indian women [24]. Possible explanations for

For peer review only - http://bmjopen.bmj.com/site/about/guidelines.xhtml

differences in incidence and disease presentation in different regions of India include differences in ethnicity and/or environmental factors. Therefore, there exists an unmet need to study distributions and presentation of endometriosis in Indian women and also characterize the different subtypes of endometriosis, superficial peritoneal endometriosis (SUP), ovarian endometrioma (OMA), and deeply infiltrating endometriosis (DIE), and the presentation of the disease in different ethnic sub-populations in India. To date, no studies have investigated the association of the GWAS-identified SNPs with endometriosis risk in the Indian population, therefore leaving one-sixth of the world population not represented in the International Endometriosis Genome Consortium (IEGC).

To achieve these goals, we are collecting prospective high-quality, standardised clinical, epidemiological, environmental, and surgical data with corresponding biological samples. Our group has been actively engaged with World Endometriosis Research Foundation's (WERF) Endometriosis Phenome and Biobanking Harmonisation Project (EPHect) for implementing the WERF-EPHect research instruments and also translating the endometriosis patient questionnaires in different Indian languages.

The Endometriosis Clinical and Genetic Research in India (ECGRI) study aims to conduct a large-scale, prospective, case-control study to investigate the clinical phenotypes and genetic risks associated with endometriosis in the Indian population.

Objectives

1. To identify and characterize endometriosis subtypes [superficial peritoneal endometriosis (SUP), cystic ovarian endometriosis or endometrioma (OMA), and deep infiltrating endometriosis (DIE)] in different geographical regions of India
2. To identify the clinical, epidemiological, environmental, life style factors associated with different endometriosis subtypes in India
3. To investigate the genetic risks associated with endometriosis in Indian population

For peer review only - http://bmjopen.bmj.com/site/about/guidelines.xhtml

Outcomes

1. Prospective standardized questionnaires [modified WERF-Endometriosis Patient Questionnaire (EPQ)], translated into regional languages, Surgical Standard Form (SSF) with data and samples collected according to WERF-EPHect standards. The questionnaire and SSF data will be used for investigating the clinical, epidemiological, and environmental and life style risk factors in women with endometriosis.
2. The GWAS analysis will be carried out to investigate the genetic risk factors in women with endometriosis. The collected data and samples will be analysed and compared between women with endometriosis and hospital controls recruited from east, north east, north, west, central and south zones of India.
3. The correlation of clinical and genomic data together with endometriosis subtype status will allow us to discover and replicate genetic risk loci for endometriosis subtypes and help in understanding pathogenesis of endometriosis and its subtypes. The clinical and genetic findings will be compared against the public databases and functional studies will be carried out in appropriate models.
4. The study will generate large scale, standardized, Indian population data on endometriosis which will be compared with other populations. The study will generate the information on different endometriosis phenotypes and their genotypes. This will be the first evidence from low middle income countries (LMICs) and India on endometriosis clinical phenotypes and risk factors. Additionally, this will be the first GWAS on endometriosis in LMICs and India on endometriosis.
5. This will help to add the endometriosis phenotype-genotype data to the International Consortium. Regional data on endometriosis sub-phenotypes, demographic, environmental, reproductive, menstrual, life style factors of endometriosis will be useful

For peer review only - http://bmjopen.bmj.com/site/about/guidelines.xhtml

in developing the strategies and policies to address the endometriosis associated morbidity in India and LMICs.

6. The evidence generated may be useful for risk prediction for endometriosis utilizing the disease phenotype and genotype data. The information on pathways involved in endometriosis would be useful for conducting further studies to develop improved treatments for endometriosis.
7. Results will provide important information on the causes and consequences of endometriosis in different regions of India and valuable comparisons for implementing improved treatments.

Methods and analysis

Study design and study sites

This is a multi-site, prospective, case-control study conducted over 5 years beginning in October 2019 and ending in September 2024. It is being conducted at 15 collaborating study sites across India covering representative Indian populations from north, north east, east, central, west and south geographical zones of India (Figure 1). The selection criteria of the study sites were as follows: (1) proven track record of gynaecologic research; (2) availability of required research manpower and infrastructure to be able to recruit the required study participants, (3) existing trusted relationship from previous collaborations. Each of these geographical zones where study participants will be recruited has significant genetic diversity which is likely to improve the ability of this research to answer the objectives of the study.

Patient and public involvement

The ECGRI study was conceptualized based on our experience and feedback we received from collaborators and patients during our previous studies [11,12]. The initial cohort of women with endometriosis provided feedback on general workflow, data collection, WERF-EPHect questionnaires.

For peer review only - http://bmjopen.bmj.com/site/about/guidelines.xhtml

Samples

After obtaining written informed consent, 15 ml of peripheral blood will be collected by venepuncture by a trained phlebotomist (10 ml in EDTA tube and 5 ml in plain serum tube) from endometriosis cases and controls.

Participants

We plan to recruit up to 2000 women with endometriosis and 2000 hospitals controls. The study participants will be recruited consecutively for 4 years at all the study sites until the desired sample size of 400 endometriosis cases and 400 controls per geographical zone is achieved.

Inclusion criteria

Inclusion Criteria for Endometriosis Cases:

- Females aged 18- 50 years, visiting clinics at the study sites for benign gynecological conditions will be initially screened and evaluated for their eligibility in the study.
- They should be willing to participate in the study, should be a resident of the geographic region for the collaborating center for a minimum period of one-year.
- Eligible females with surgically confirmed diagnosis of endometriosis on laparoscopy will be recruited as endometriosis cases. Endometriosis will be further confirmed on histology wherever possible.
- The laparoscopic images of the endometriosis lesions will be provided by the clinical collaborators along with the completed WERF-EPHect Standard Surgical Form.
- Endometriosis cases will be sub classified as superficial peritoneal endometriosis (SUP), cystic ovarian endometriosis or endometrioma (OMA) and deep infiltrating endometriosis (DIE). Endometriosis cases with any additional subtypes/ phenotypic features other than SUP, OMA and DIE will also be included.

For peer review only - http://bmjopen.bmj.com/site/about/guidelines.xhtml

- Women with family history of endometriosis will also be invited to participate with one proband per family included in the main study. Affected and non-affected relatives of the proband up to minimum three generations will be recruited wherever possible. Data of familial endometriosis cases will be analyzed separately from the sporadic endometriosis cases.

Inclusion Criteria for Hospital Controls:

- The appropriate comparison group having the same opportunity to develop endometriosis and representative of the same geographical location as endometriosis cases will be selected from all the collaborating centres.
- These study participants will be the hospital-based controls residing in the geographic region of the collaborating centres for minimum of one-year period.
- Female visitors aged 18-50 years, unrelated to the endometriosis cases, who are visiting the collaborating centres during the same time period of recruitment of endometriosis cases will be eligible for enrolment as controls.

Exclusion Criteria:

- Female visitors having history of endometriosis will be excluded from the control group.

Participant enrolment

Screening of cases and controls

The flow chart of study participant recruitment and study processes is represented in Figure 2. Eligible study participants (Endometriosis cases and hospital controls) will be screened and identified by the Clinical Collaborators at each study site. Clinical collaborators are experienced gynaecological laparoscopic surgeons having expertise in diagnosis and management of endometriosis.

Informed Consent

For peer review only - http://bmjopen.bmj.com/site/about/guidelines.xhtml

Research staff who have received study specific training will approach the study participants identified by the Clinical Collaborator. They will provide a Participant Information Sheet (PIS) that includes the purpose and procedures of the study and carefully explain the same to the participants in their local language. They will explain to participants that participation in this study is entirely voluntary and participants are free to withdraw from the study at any point without affecting their medical care and services. A copy of the signed informed consent and PIS will be given to participants in the language they read and understand. Languages include: Hindi, Marathi, Malayali, Assamese, Kannada, Bengali and English. The original signed consent form will be retained at the study site.

Interventions

Non- Clinical Interventions

WERF-EPHect Endometriosis Patient Questionnaire (Modified)

To determine the differences in endometriosis subtypes, presentation and disease severity between geographical regions and ethnic groups in India, data will be collected with WERF-EPHect endometriosis patient questionnaire (EPQ) modified version form [25]. After obtaining the informed consent from the participants, the Research staff will administer the modified WERF-EPHect EPQ under the supervision of Clinical Collaborators for both cases and controls. The modified EPQ is designed to capture phenotype data across the life cycle particularly relevant due to delay in diagnosis. Pain related questionnaires on 11-point numerical rating scale (NRS) (0-10) will capture four separate domains of pain (continuous, intermittent, neuropathic and affective). Information on depression, anxiety, and Health-related Quality of Life will be captured. Menstrual history including age at menarche, menstrual irregularities, last date of menstrual period, frequency, duration of flow and menstrual cycle length will be recorded. Use of hormonal treatments, length of time and type of treatment will be obtained. Information on subfertility, age at the start of

For peer review only - http://bmjopen.bmj.com/site/about/guidelines.xhtml

each pregnancy, type of fertility treatment used for each pregnancy, and pregnancy outcome (if applicable), live births, type of delivery and pregnancy complications will be captured. Detailed history of medical and surgical treatments will also be obtained. The sociodemographic, personal and life style data including age, ethnicity, education, anthropometric measurements, history of smoking, alcohol use and exercise will be captured by modified EPQ. Information on occupation and history of residence in each decade of life will also be captured (Supplementary Table 1).

Standard Surgical Form

The surgical data will be collected by the Clinical Collaborators using WERF-EPHect Standard Surgical Form [SSF] [26]. EPHect SSF consists of 2 parts designed to capture all relevant visual information of the endometriosis lesion phenotype and the surgical treatment including type, duration of the procedure and requires approximately 1-3 minutes to complete the SSF. The first part of SSF includes detailed information on clinical covariates and requires detailed information related to the current menstrual cycle, current hormonal treatment, history of previous surgery for endometriosis, ultrasound or MRI findings before the procedure. The second part focuses on detailed visual information on intraoperative findings such as extent, exact location, and color of endometriotic lesions, with a specific focus on endometrioma dimensions and endometriotic nodules. It also includes information on endometriosis fertility index. Based on the EPHect SSF data, the phenotype characterization of endometriosis will be carried out into superficial peritoneal endometriosis (SUP), cystic ovarian endometriosis or endometrioma (OMA), and deep infiltrating endometriosis (DIE). From the EPHect SSF data, rAFS (Revised American Fertility Society) score will be calculated and endometriosis cases will be grouped into Stage I/II or Stage III/IV.

Familial Endometriosis

For peer review only

For peer review only - http://bmjopen.bmj.com/site/about/guidelines.xhtml

The clinical data of female relatives of the proband will be collected as per the WERF-EPHect and family history of the proband will be documented through pedigree analysis. This will include both affected and non-affected relatives of the proband up to minimum three generations wherever possible.

Laparoscopic images documentation

The collaborators (gynaecological laparoscopic surgeons) at each study site will obtain laparoscopic images of the different endometriotic lesions and adhesions. The soft copies of the labelled laparoscopic images will be uploaded on the ECGRI electronic database. The

Power calculations

Over a period of 4 years, the study team will recruit a total of 2000 endometriosis cases and 2000 hospital controls across five geographical zones of India. To detect odds ratio of ≥ 2 with 95% confidence interval and 90% power with 10% missing or incomplete WERF-EPHect data collection forms, the required sample size per zone = 360. In each geographical zone, four hundred endometriosis cases and four hundred hospital controls will be recruited.

To replicate results from the European GWAS (P=0.05/27 or P<0.0018) and test all 27 "hits" using either average effect size or effect size for the smallest effect (Risk allele with frequency 0.3 and genotype relative risk 1.22 with 80 % power), total sample size required is as follows: Endometriosis Cases (n= 1250), Hospital Controls (n=1250). For conducting discovery GWAS in Indian population (effect size to detect at P<5x10-8), a total sample size of 2000 endometriosis and 2000 hospital controls will be recruited. To conduct replication GWAS for novel risk loci in Indian women with endometriosis an Independent set of endometriosis cases and controls will be recruited based on discovery GWAS results.

Electronic database

For peer review only - http://bmjopen.bmj.com/site/about/guidelines.xhtml

A dedicated website is developed for the project (https://ecgri.in). Electronic data collection software is developed for online data entry (http://app.ecgri.in/login). The Project assistants will enter the data electronically from the paper copies of the WERF-EPHect Endometriosis Patient Questionnaire and Standard Surgical Forms. The online electronic data collection software (http://app.ecgri.in/login) is hosted on a secure server at the Biomedical Informatics Division of ICMR- National Institute for Research in Reproductive Health (NIRRH), Mumbai. The central database will allow online, real time data entry with the option of online editing. Project assistants and Clinical Collaborators will have access for data entry, data editing only for their respective centres and will not have any access to the whole database.

Research Team Training and Quality Controls

Research staff have received study specific training including recruitment of study participants, obtaining informed consent, counselling the study participates, data collection as per WERF-EPHect EPQ, sample collection, processing, storage and transport as per WERF-EPHect Standard Operating Procedures (SOPs). Intense Training was provided to the research staff, Clinical Collaborators on selection and recruitment of study participants, data collection as per WERF-EPHect Questionnaire and Standard Surgical Forms (SSF), sample collection, processing and transportation as per WERF-EPHect SOPs and online data entry. A copy of the training manual is provided to all study sites. A Data analyst will coordinate with Clinical Collaborators and research staff for timely entry of the data and transportation of samples. The Data Analyst will report to RG at ICMR-NIRRH.

RD will provide training to the Data Analyst on data collection, quality control, data entry and online editing, data analysis, development and maintenance of databases at ICMR-NIRRH. The research staff will report to their respective Clinical Collaborators at participating study sites. The Data Analyst will supervise, conduct regular quality control checks and clean the data to identify and rectify the errors by comparing the hard and soft

For peer review only - http://bmjopen.bmj.com/site/about/guidelines.xhtml

copies of data collection instruments. RG will coordinate with Clinical Collaborators for recruitment of study participants, data collection, data entry, blood sample collection-processing and transport as per the WERF-EPHect research instruments. Video meetings will be organised with all Collaborating Centres, research team at ICMR-NIRRH to review the progress and ensure adherence to protocol. Detailed checklist is provided to the collaborating centres to ensure adherence to the protocol. There will be weekly auditing of data collection and data entry for each site by the Clinical Collaborator to ensure that the protocols are followed and data entry is done promptly as soon as the data is collected. Data Analyst will coordinate with the collaborating centres every week through email/video/teleconference. There will be a detailed regular monitoring of a random sample of individuals collected at each site. The centralised database at NIRRH, Mumbai will be checked and updated every week to provide regular reports from each study site and across the study on the number of cases and controls collected with additional information on completion of all parts of the protocol for each individual. RG will provide regular feedback to each and all sites on issues of compliances every month. The hard copies of the original documents (WERF-EPHect instruments- modified EPQ, SSF) will be transported to the ICMR-NIRRH for cross checking the data entry. These data forms will be stored as per the National ethical guidelines for biomedical and health research involving human participants [27].

Collection, processing and storage of blood samples

The standard operating procedures (SOPs) have been established and are being implemented in RG's laboratory as per the WERF-EPHect collection protocol [28]. The cases and controls will be recruited simultaneously during the study period. After obtaining informed consent, 15 ml peripheral blood (10 ml in EDTA tube and 5 ml in plain serum tube) will be collected in an appropriate setting. Resulting plasma/serum will be stored in aliquots (500µl) prior to freezing in - 80º C freezer with power back up.

For peer review only - http://bmjopen.bmj.com/site/about/guidelines.xhtml

Collected samples will be separated into its 3 derivatives (Serum, Plasma and Buffy coat). All the details of collection will be recorded on the EPHect Biospecimens form.

Sample Processing, Genotyping and Quality Control

Genomic DNA will be extracted from buffy coats using the QIAamp DNA Blood Midi kit (Qiagen) using manufacturer's instructions. The concentration of each DNA sample will be determined. The DNA samples that pass minimum requirements for sample quality will be sent to the commercial facility for genome wide genotype analysis using appropriate genotyping platform (Illumina/Afffimatrix) specific to Asian Indian populations. Standard genotype quality control procedures will be performed using the software PLINK. SNPs with a high missing rate (>5%), low minor allele frequency ($<1x10^{-4}$) and with Hardy-Weinburg Equilibrium p<10-6 will be excluded along with samples with low call rates (<95%), extreme heterozygosity, high genetic relatedness and any ancestry outliers. Standard methods for quality control and genetic associations will be adopted as reported earlier [21]. Following quality control, genotypes will be imputed to the 1000 Genomes Phase 3 reference panel using the Michigan Imputation Server. Endometriosis cases with a family history either in grandmother, mother or sister will be recruited for enrichment sampling. The genotype data of familial endometriosis cases will be analysed separately. Exome sequencing will be carried out for identification of the genes associated with familial endometriosis.

Data analysis plan

We will use SPSS, GraphPad Prism, R and plink software for analysis. The principal analyses will be for main outcome measures of the incidence of endometriosis, disease subtypes and disease severity determined from the clinical data. We will conduct conditional logistic regression to analyse effects of geographical region and ethnicity (defined from genetic data) adjusting for age, recruitment clinic, and other confounding variables that may be identified

For peer review only - http://bmjopen.bmj.com/site/about/guidelines.xhtml

from initial inspection of the data. Disease presentation and endometriosis severity between regions and ethnic groups in India will be analysed. Co-morbidity and environmental exposures between regions will be compared and correlated with disease severity and distribution of lesions. Genotype data will be used to define ethnicity for the phenotypic analysis. Association between previously identified endometriosis risk loci and endometriosis status in our Indian cohort will be analyzed on imputed genotypes using SNPTEST v2. An additive logistic regression model will be used to test association whilst controlling for population stratification by including age and the top 5 principle components as covariates in the model. A study wide significance threshold of $p<0.05/n$ will be applied where n is the number of loci tested. Subsequently, we will perform a genome wide association analysis using SNPTEST to estimate adjusted odds ratios and corresponding 95% CI between each available SNP and endometriosis case/control status. The sub-phenotype association analysis will be performed for rAFS stage, site, geographical regions (North, East, North East, South, West and Central India), infertility and associated comorbidities (reproductive, metabolic, inflammatory, autoimmune disorders, COVID-19 etc.).

Functional studies aimed at delineating the causal genetic variants and biological mechanisms underlying the observed statistical associations with endometriosis risk will be carried based on the results of the discovery GWAS. Meta-analysis in collaboration with IEGC and other Genomic groups will be explored.

Plans for data management, curation, storage and confidentiality

Clinical and genomic datasets from Indian women with and without endometriosis will be captured in a central database (http://app.ecgri.in/login), maintained and regularly updated at ICMR-NIRRH. The database is developed on a robust platform including the ability to allow online, real time data entry. Demographic data will be kept in separate tables and records

For peer review only - http://bmjopen.bmj.com/site/about/guidelines.xhtml

identifying the study participants will be kept confidential. Data entry and data access will be independently controlled and identifiable data held behind secure firewalls according to the ICMR- National Guidelines for Biomedical and Health Research Involving Human Participants [27]. Data collected from different sites will be held separately and only specific individuals within a research team will be able to access and edit data. Where possible, data will be captured using online tools, including the clinical data in the WERF-EPHect instruments. Data collected from paper questionnaires will be entered and verified independently. The quality of the clinical and genomic data will be regularly monitored. The genotype data will be stored in a separate computer with sufficient memory space and backup data will be stored in hard disks. The clinical and genomic data will be carefully linked by individual identification numbers to maintain confidentiality of the participants and the key will be held by RG at ICMR-NIRRH.

Quality assurance procedures and reporting

The study will be monitored by Institutional Ethics Committee (IEC) of the study sites and ICMR-NIRRH. The study protocol will also be monitored by Health Ministry Screening Committee (HMSC) of Government of India. Periodic progress reports are required to be submitted to HMSC, IEC of ICMR-NIRRH, study sites. Periodic progress report will be submitted to the funding agency.

Publications

Results will be presented and discussed with endometriosis patient groups in India and elsewhere through Endometriosis Society India (ESI) and World Endometriosis Society (WES). RG and GWM are currently actively interacting and communicating with World Endometriosis Society (WES) and World Endometriosis Research Foundation (WERF). The research findings from the study will be published in peer reviewed, high impact factor journals. The findings will be disseminated at national and international conferences.

For peer review only

For peer review only - http://bmjopen.bmj.com/site/about/guidelines.xhtml

Study status

The present study was conceptualized and designed in 2019. The first participant was recruited on January 2020, the study is ongoing and desired sample size will be achieved by September 2023. The baseline characteristics of endometriosis cases and hospital controls recruited till 31st January 2021 are shown in Table 1. The distributions of isolated and overlap of endometriosis sub-phenotypes in the initial cohort of 155 women with endometriosis is presented in Figure 3.

For peer review only

Discussion

To the best of our knowledge, this will be the first large scale study investigating endometriosis sub-phenotypes, risk factors and conducting a GWAS in Indian women with endometriosis. The study will lead to establishment of endometriosis bio-repository of serum, plasma, DNA samples as well as establishment of clinical, genomic database of endometriosis in India. The results of this study will improve the understanding of pathogenesis of endometriosis and its subtypes and also form the basis to join the global network of International Consortium on Endometriosis and contribute for improving patient healthcare in India and for patients everywhere.

Funding

This study is funded by the DBT Wellcome India Alliance (Grant no. IA/CPHI/18/1/503933). The sponsor was not involved in the study design, in the writing of the report and in the decision to submit the paper for publication.

Author contributions

RG conceived the study and wrote the protocol with GWM. MP, PP, SS, KP, PDM, BJ, CS, NB, KK, NS, SM AH, US, AS and MS consented participants, collected data and samples, AB conducted quality controls, data analysis. RD, GM and SDM provided inputs in revision of data collection tools and development of electronic database. Data collection was

For peer review only - http://bmjopen.bmj.com/site/about/guidelines.xhtml

facilitated by and conducted in compliance with the World Endometriosis Research Foundation Endometriosis Phenome and Biobanking Harmonisation Project (WERF-EPHect).

Competing interests

None declared.

Ethics approval:

Ethics approvals have been granted by ICMR-NIRRH (D/ICEC/Sci-87/91/2019; D/ICEC/Sci-165/170/2020) and Institutional Ethics Committees of study sites (ECR/146/Inst/KA/2013/RR-19; AMC/EC/1304; AIIMS/IEC/2019-20/884; CH/IEC/19/004; 734/IEC/-AIIMSRPR/2019; IEC-NWMH/AP/2019/033-V1; 1320/IEC-AIIMSRPR/2020; /839EC/Pharmac/GMC/NGP/; ECR/262/Inst/UP/2013/RR-19). The study has been approved by Health Ministry Screening Committee (HMSC) of Government of India. (HMSC No.: 2019-7620).

Data sharing

All data relevant to the study are included in the article or uploaded as supplementary information.

Acknowledgments

The authors thank the members of the ECGRI study group. Dr. Shailaja Patil, Dr Manisha Shembekar, Dr Parul Sharma Saoji, Jishna RP, Nimisha Bhuyan, Akhila S., Kiran Kharsodiya, Ashwini Patel, Merlin Pious, Aarti Kushwaha, Sudeshna Bakshi, Akshatha Shetty, Nadha Abdul Razack, Neha Yadav are sincerely acknowledged for assistance in data collection. Mr. Ram Barai is acknowledged for assistance in ECGRI electronic database development. Dr. Yogesh Kalkonde and Dr. Rajesh Dikshit are acknowledged for critical inputs on the study design. This work was supported by the DBT Wellcome Trust India Alliance Fellowship [Grant no. IA/CPHI/18/1/503933] awarded to RG.

For peer review only

References

1 Zondervan KT, Becker CM, Missmer SA. Endometriosis. *N Engl J Med* 2020;**382**:1244–56.

2 Population ages 15-64 (% of total population) | Data. https://data.worldbank.org/indicator/SP.POP.1564.TO.ZS (accessed 3 Feb 2021).

3 Population, total - India | Data. https://data.worldbank.org/indicator/SP.POP.TOTL?locations=IN (accessed 25 Jan 2021).

4 Rogers PAW, Adamson GD, Al-Jefout M, *et al.* Research Priorities for Endometriosis: Recommendations From a Global Consortium of Investigators in Endometriosis. *Reprod Sci* 2017;**24**:202–26.

5 Grimstad FW, Decherney A. A Review of the Epigenetic Contributions to Endometriosis: *Clin Obstet Gynecol* 2017;**60**:467–76.

6 Koninckx PR, Ussia A, Adamyan L, *et al.* Pathogenesis of endometriosis: the genetic/epigenetic theory. *Fertil Steril* 2019;**111**:327–40.

7 Rahmioglu N, Macgregor S, Drong AW, *et al.* Genome-wide enrichment analysis between endometriosis and obesity-related traits reveals novel susceptibility loci. *Hum Mol Genet* 2015;**24**:1185–99.

8 Fung JN, Mortlock S, Girling JE, *et al.* Genetic regulation of disease risk and endometrial gene expression highlights potential target genes for endometriosis and polycystic ovarian syndrome. *Sci Rep* 2018;**8**:11424–11424.

9 Gajbhiye R, Fung JN, Montgomery GW. Complex genetics of female fertility. *NPJ Genom Med* 2018;**3**:29–29.

10 Montgomery GW, Giudice LC. New Lessons about Endometriosis — Somatic Mutations and Disease Heterogeneity. *N Engl J Med* 2017;**376**:1881–2.

11 Gajbhiye R, Bendigeri T, Ghuge A, *et al.* Panel of Autoimmune Markers for Noninvasive Diagnosis of Minimal–Mild Endometriosis: A Multicenter Study. *Reprod Sci* 2017;**24**:413–20.

12 Gajbhiye R, Sonawani A, Khan S, *et al.* Identification and validation of novel serum markers for early diagnosis of endometriosis. *Hum Reprod* 2012;**27**:408–17.

For peer review only - http://bmjopen.bmj.com/site/about/guidelines.xhtml

13 Gajbhiye R, McKinnon B, Mortlock S, *et al.* Genetic Variation at Chromosome 2q13 and Its Potential Influence on Endometriosis Susceptibility Through Effects on the IL-1 Family. *Reprod Sci* 2018;:1933719118768688.

14 Farland LV, Shah DK, Kvaskoff M, *et al.* Epidemiological and Clinical Risk Factors for Endometriosis. In: D'Hooghe T, ed. *Biomarkers for Endometriosis: State of the Art*. Cham: : Springer International Publishing 2017. 95–121.

15 Shafrir AL, Farland LV, Shah DK, *et al.* Risk for and consequences of endometriosis: A critical epidemiologic review. *Best Pract Res Clin Obstet Gynaecol* 2018;**51**:1–15.

16 Zondervan KT, Becker CM, Missmer SA. Endometriosis. *N Engl J Med* 2020;**382**:1244–56.

17 Zondervan KT, Becker CM, Koga K, *et al.* Endometriosis. *Nat Rev Dis Primers* 2018;**4**:9.

18 Treloar SA, O'Connor DT, O'Connor VM, *et al.* Genetic influences on endometriosis in an Australian twin sample. sueT@qimr.edu.au. *Fertil Steril* 1999;**71**:701–10.

19 Saha R, Pettersson HJ, Svedberg P, *et al.* Heritability of endometriosis. *Fertil Steril* 2015;**104**:947–52.

20 Fung JN, Montgomery GW. Genetics of endometriosis: State of the art on genetic risk factors for endometriosis. *Best Pract Res Clin Obstet Gynaecol* 2018;**50**:61–71.

21 Sapkota Y, Steinthorsdottir V, Morris AP, *et al.* Meta-analysis identifies five novel loci associated with endometriosis highlighting key genes involved in hormone metabolism. *Nat Commun* 2017;**8**:15539.

22 Montgomery GW, Mortlock S, Giudice LC. Should Genetics Now Be Considered the Pre-eminent Etiologic Factor in Endometriosis? *JMIG* 2020;**27**:280–6.

23 Nilufer R, Karina B, Paraskevi C, *et al.* Large-scale genome-wide association meta-analysis of endometriosis reveals 13 novel loci and genetically-associated comorbidity with other pain conditions. *bioRxiv* 2018;:406967.

24 Bendigeri T, Warty N, Sawant R, *et al.* Endometriosis: Clinical Experience of 500 Patients from India. *Indian Pract* 2015;**68**:34-40–40.

25 Vitonis AF, Vincent K, Rahmioglu N, *et al.* World Endometriosis Research Foundation Endometriosis Phenome and Biobanking Harmonization Project: II. Clinical and covariate phenotype data collection in endometriosis research. *Fertil Steril* 2014;**102**:1223–32.

26 Becker CM, Laufer MR, Stratton P, *et al.* World Endometriosis Research Foundation Endometriosis Phenome and Biobanking Harmonisation Project: I. Surgical phenotype data collection in endometriosis research. *Fertility and Sterility* 2014;**102**:1213–22.

27 Mathur R, Swaminathan S. National ethical guidelines for biomedical & health research involving human participants, 2017: A commentary. *IJMR* 2018;**148**:279.

For peer review only

For peer review only - http://bmjopen.bmj.com/site/about/guidelines.xhtml

28 Rahmioglu N, Fassbender A, Vitonis AF, *et al.* World Endometriosis Research Foundation Endometriosis Phenome and Biobanking Harmonization Project: III. Fluid biospecimen collection, processing, and storage in endometriosis research. *Fertil Steril* 2014;**102**:1233–43.

Figure Legends

Figure 1: Study sites of ECGRI across different geographic regions of India

Figure 2: Flow chart of ECGRI study participant recruitment and study processes.

Abbreviations: WERF-EPHect, World Endometriosis Research Foundation – Endometriosis Phenome and Biobanking Harmonisation Project; EPQ, Endometriosis Patient Questionnaire; SSF, Standard Surgical Form; SOP, Standard Operating Processes; QC, Quality Control; ICMR-NIRRH, Indian Council of Medical Research – National Institute for Research in Reproductive Health.

Figure 3: Distribution of isolated and overlap of endometriosis subtypes in Indian women.

Abbreviations: SUP, Superficial Peritoneal Endometriosis; OMA, Ovarian Endometriosis/ Endometrioma; DIE, Deep Infiltrating Endometriosis

For peer review only

For peer review only - http://bmjopen.bmj.com/site/about/guidelines.xhtml

Table 1: Sociodemographic, clinical and surgical characteristics of Indian women with endometriosis cases and hospital controls

Characteristics		Cases	Controls	P value
**Demographics**		(n=159)	(n=148)	
Age (years), Mean ± SD		32.5 ± 7.1	30.3 ± 7.2	0.550*
BMI (kg/m^2), Mean ± SD		24.0 ± 4.4	24.5 ± 4.2	0.889*
BMI (kg/m^2) categories	Underweight (≤18.4)	10 (6.5)	5 (3.5)	0.653†
	Normal (18.5 – 22.9)	56 (36.4)	50 (35)	
	Overweight (23 – 24.9)	26 (16.9)	27 (18.9)	
	Obese (≥25)	62 (40.3)	61 (42.7)	
Education level	Up to Higher Secondary	45 (28.3)	62 (41.9)	0.016‡
	Graduate and above	114 (71.7)	86 (58.1)	
Employment	Government/ Private job	45 (28.5)	78 (52.7)	<0.001†
	Self employed	17 (10.8)	19 (12.8)	
	Unemployed	85 (53.8)	31 (20.9)	
	Student	11 (7)	20 (13.5)	
Annual family income (in Rs.)	≤100,000	65 (41.1)	65 (44.5)	0.013†
	>100,000 - 500,000	60 (38)	68 (46.6)	
	≥500,000	33 (20.9)	13 (8.9)	

For peer review only

For peer review only - http://bmjopen.bmj.com/site/about/guidelines.xhtml

Marital status**	Married	133 (83.6)	87 (58.8)	<0.001†
	Unmarried	23 (14.5)	58 (39.2)	
**Clinical**				
Age at Menarche (in years)	≤12	55 (34.6)	38 (25.7)	0.106‡
	>12	104 (65.4)	110 (74.3)	
Regularity of menses	Regular	121 (82.9)	128 (94.1)	0.005‡
	Irregular	25 (17.1)	8 (5.9)	
Menstrual Bleeding		n= 144	n= 134	
Duration (in days), Mean ± SD		4.3 ± 1.7	4.2 ± 2.5	0.435*
Menstrual Cycle Length (in days)	24-31	107 (74.3)	114 (85.1)	0.037‡
	>31	37 (25.7)	20 (14.9)	
Menstrual Blood Loss (in ml), Mean ± SD	At Heaviest (n=146)	44.8 ± 12.8	41.7 ± 10.9	<0.001*
	On average (n=136)	31.1 ± 14.8	26.9 ± 14.3	0.538*
Pain symptoms				
Dysmenorrhea	Yes	143 (89.9)	84 (56.8)	<0.001‡
	No	16 (10.1)	64 (43.2)	
Dyspareunia	Yes (n=62)	47 (36.7)	15 (15.8)	0.001‡
	No (n=161)	81 (63.3)	80 (84.2)	
**Surgical**				
Endometriosis subtypes dominant# (n=155)	SUP	33 (21.3)	-	-
	OMA	98 (63.2)	-	-
	DIE	22 (14.2)	-	-
	Scar endometriosis	2 (1.3)	-	-
Endometriosis severity	rAFS stage I	31 (20)	-	-

For peer review only - http://bmjopen.bmj.com/site/about/guidelines.xhtml

(n=152)	rAFS stage II	16 (10.3)	-	-
	rAFS stage III	33 (21.3)	-	-
	rAFS stage IV	72 (46.5)	-	-
Recurrent endometriosis (n=15)	Once	12 (80)	-	-
	Twice	2 (13.3)	-	-
	Thrice	1 (6.7)	-	-
EFI score (range 0-10) (n=154), Mean ± SD		5.6 ± 2.2	-	-

Abbreviations: BMI, Body Mass Index; rAFS, Revised American Fertility Society; EFI, Endometriosis Fertility Index

Data presented as n (%), unless specified otherwise.

*t-test

†Pearson Chi-square test

‡Fisher's Exact test

# The disease was categorized as follows: isolated sub-phenotypes (SUP or OMA or DIE) and overlap of sub-phenotypes. All the women with endometriosis irrespective of the overlap were combined and further classified as dominant SUP or OMA or DIE based on the dominance (severity of the disease and size of lesions) of the superficial endometriotic lesions, or adhesions or endometrioma or deep endometriotic lesions within these sub-phenotypes.

**3 endometriosis cases and 3 controls were either widowed or divorced. In the current cohort, 74 women were infertile, among which 61 (91%) cases and 3 (42.9%) controls had primary infertility, while 6 (9%) cases and 4 (57.1%) controls had secondary infertility. However, due to small sample size, the groups were not comparable.

For peer review only - http://bmjopen.bmj.com/site/about/guidelines.xhtml

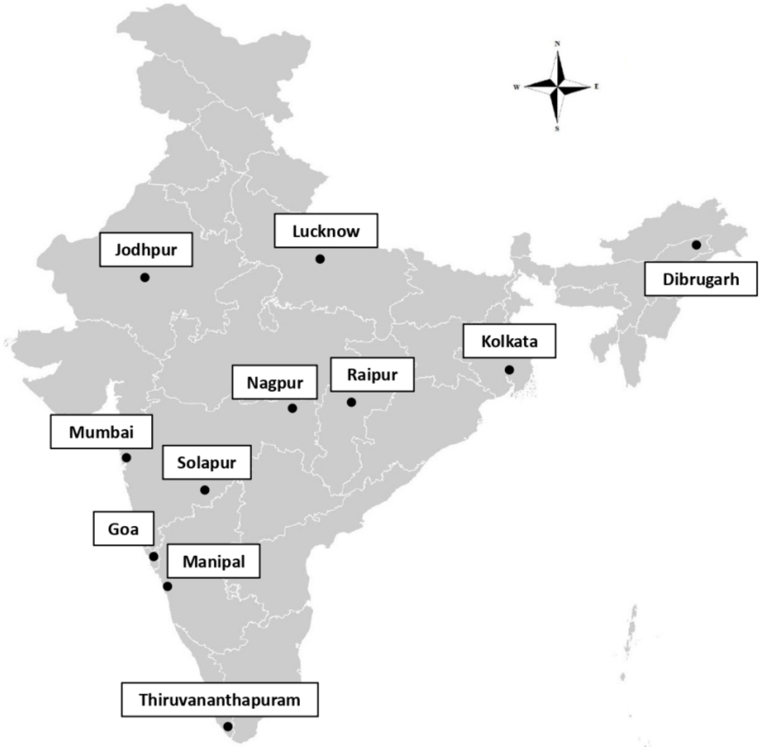

State	Study sites	State	Study sites
Assam	Assam Medical College, Dibrugarh	Maharashtra	Worli Hospital for Women, Mumbai; Nowrosjee Wadia Maternity Hospital, Mumbai; Naval Maternity Endoscopy & Infertility Center, Mumbai
Uttar Pradesh	King George's Medical University, Lucknow; Morpheus Lucknow Fertility Center, Lucknow		
Rajasthan	All India Institute of Medical Sciences (AIIMS), Jodhpur		
West Bengal	Spectrum Clinic and Endoscopy Research Institute, Kolkata	Maharashtra	Naval Maternity Endoscopy & Infertility Center, Solapur
Chhattisgarh	AIIMS, Raipur; Sai Baba Nursing Home, Raipur	Karnataka	Kasturba Medical College, Manipal
Maharashtra	Government Medical College, Nagpur; Omega Hospital, Nagpur	Kerala	Credence Hospital, Thiruvananthapuram

Study sites of ECGRI across different geographic regions of India

95x137mm (300 x 300 DPI)

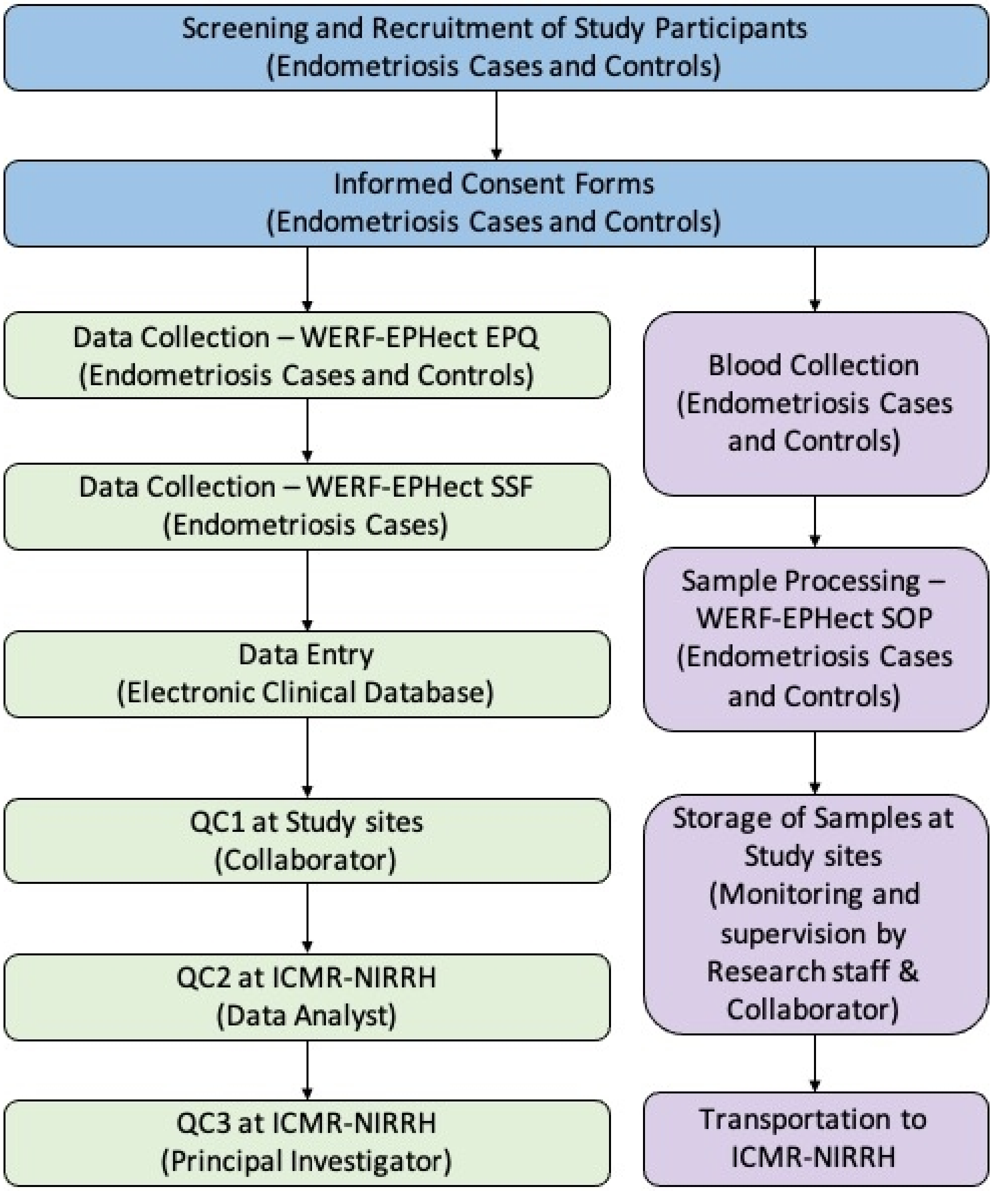

Flow chart of ECGRI study participant recruitment and study processes.
Abbreviations: WERF-EPHect, World Endometriosis Research Foundation – Endometriosis Phenome and Biobanking Harmonisation Project; EPQ, Endometriosis Patient Questionnaire; SSF, Standard Surgical Form; SOP, Standard Operating Processes; QC, Quality Control; ICMR-NIRRH, Indian Council of Medical Research – National Institute for Research in Reproductive Health.

41x50mm (300 x 300 DPI)

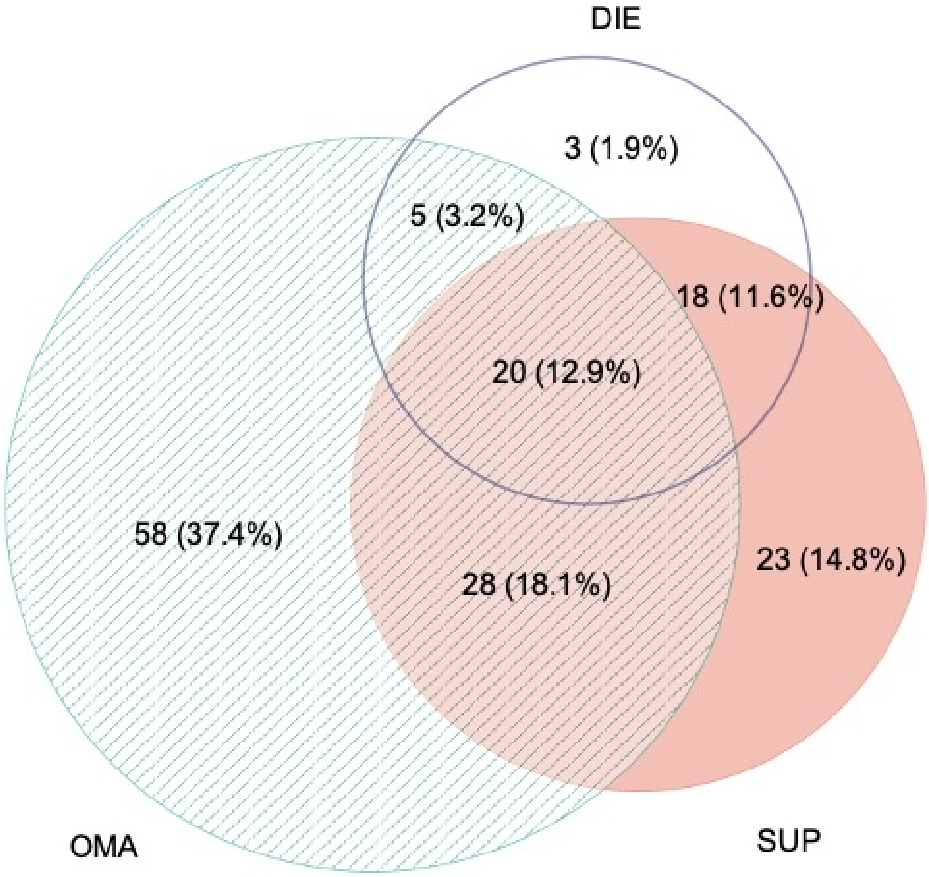

Figure 3: Distribution of isolated and overlap of endometriosis subtypes in Indian women. Abbreviations: SUP, Superficial Peritoneal Endometriosis; OMA, Ovarian Endometriosis/ Endometrioma; DIE, Deep Infiltrating Endometriosis

43x40mm (300 x 300 DPI)

Supplementary Table 1: Clinical, epidemiological, reproductive, lifestyle and environmental characteristics captured using modified Endometriosis Patient Questionnaire (EPQ)

**Characteristics**	**Variables**
**Menstrual and Reproductive factors**	Age at menarche, menstrual cycle length, menstrual volume, dysmenorrhea, current oral contraceptive use, hormones, history of infertility
**Pregnancy and Fertility**	Age at each pregnancy, parity, fertility treatment, outcome of pregnancy, history of miscarriage, ectopic pregnancy, vaginal delivery, caesarean section, breast feeding, natural pregnancy/ ART, complications related to pregnancy- GDM, PIH, Preterm birth
**Health-related Quality of life**	Thoughts and feelings associated with pain, severity grading scale (0-10), Life time details - pelvic pain, pain during or after vaginal intercourse, treatment for pain, gastrointestinal and urinary symptoms etc.
**Socio-demographic, personal and lifestyle factors**	Geographic location, age, educational level, marital status, age at marriage, employment, family income, height, weight, BMI, waist -to- hip ratio, ethnicity, cigarette smoking, alcohol use, caffeine intake, regular exercise, night shift work, natural hair color, eye color, skin color, body shape
**Medical and surgical**	History of associated diseases- cancer, autoimmune diseases<br>endometriosis or chronic pelvic pain history- personal and family (if known), details of medical and surgical treatment
**Environmental factors**	Residence in each decade of life, pollution exposure, smoking environment,<br>sun exposure, occupational history, pesticide exposure, any other relevant information

Abbreviations: ART, Assisted Reproductive Technology; GDM, Gestational Diabetes Mellitus; PIH, Pregnancy-induced hypertension; BMI, Body mass index

For peer review only

STROBE 2007 (v4) Statement—Checklist of items that should be included in reports of *case-control studies*

Section/Topic	Item #	Recommendation	Reported on page #
**Title and abstract**	1	(*a*) Indicate the study's design with a commonly used term in the title or the abstract	1,4
		(*b*) Provide in the abstract an informative and balanced summary of what was done and what was found	4,5
**Introduction**			
Background/rationale	2	Explain the scientific background and rationale for the investigation being reported	6-8
Objectives	3	State specific objectives, including any prespecified hypotheses	8
**Methods**			
Study design	4	Present key elements of study design early in the paper	10
Setting	5	Describe the setting, locations, and relevant dates, including periods of recruitment, exposure, follow-up, and data collection	10
Participants	6	(*a*) Give the eligibility criteria, and the sources and methods of case ascertainment and control selection. Give the rationale for the choice of cases and controls	11,12
		(*b*) For matched studies, give matching criteria and the number of controls per case	11, 15
Variables	7	Clearly define all outcomes, exposures, predictors, potential confounders, and effect modifiers. Give diagnostic criteria, if applicable	13-15
Data sources/ measurement	8*	For each variable of interest, give sources of data and details of methods of assessment (measurement). Describe comparability of assessment methods if there is more than one group	18
Bias	9	Describe any efforts to address potential sources of bias	18,19
Study size	10	Explain how the study size was arrived at	15
Quantitative variables	11	Explain how quantitative variables were handled in the analyses. If applicable, describe which groupings were chosen and why	28
Statistical methods	12	(*a*) Describe all statistical methods, including those used to control for confounding	18,19
		(*b*) Describe any methods used to examine subgroups and interactions	18,19
		(*c*) Explain how missing data were addressed	NA
		(*d*) If applicable, explain how matching of cases and controls was addressed	11,12
		(*e*) Describe any sensitivity analyses	Not applicable
**Results**			

For peer review only

Participants	13*	(a) Report numbers of individuals at each stage of study—eg numbers potentially eligible, examined for eligibility, confirmed eligible, included in the study, completing follow-up, and analysed	20
		(b) Give reasons for non-participation at each stage	Nil
		(c) Consider use of a flow diagram	Figure 2
Descriptive data	14*	(a) Give characteristics of study participants (eg demographic, clinical, social) and information on exposures and potential confounders	26,27,28
		(b) Indicate number of participants with missing data for each variable of interest	Nil
Outcome data	15*	Report numbers in each exposure category, or summary measures of exposure	Not applicable
Main results	16	(*a*) Give unadjusted estimates and, if applicable, confounder-adjusted estimates and their precision (eg, 95% confidence interval). Make clear which confounders were adjusted for and why they were included	Not applicable
		(*b*) Report category boundaries when continuous variables were categorized	26,27,28
		(*c*) If relevant, consider translating estimates of relative risk into absolute risk for a meaningful time period	Not applicable
Other analyses	17	Report other analyses done—eg analyses of subgroups and interactions, and sensitivity analyses	Nil
**Discussion**			
Key results	18	Summarise key results with reference to study objectives	21
Limitations	19	Discuss limitations of the study, taking into account sources of potential bias or imprecision. Discuss both direction and magnitude of any potential bias	5
Interpretation	20	Give a cautious overall interpretation of results considering objectives, limitations, multiplicity of analyses, results from similar studies, and other relevant evidence	Not applicable
Generalisability	21	Discuss the generalisability (external validity) of the study results	21
**Other information**			
Funding	22	Give the source of funding and the role of the funders for the present study and, if applicable, for the original study on which the present article is based	21

*Give information separately for cases and controls in case-control studies and, if applicable, for exposed and unexposed groups in cohort and cross-sectional studies.

Note: An Explanation and Elaboration article discusses each checklist item and gives methodological background and published examples of transparent reporting. The STROBE checklist is best used in conjunction with this article (freely available on the Web sites of PLoS Medicine at http://www.plosmedicine.org/, Annals of Internal Medicine at http://www.annals.org/, and Epidemiology at http://www.epidem.com/). Information on the STROBE Initiative is available at www.strobe-statement.org.

For peer review only

BMJ Open

Protocol for a case-control study investigating the clinical phenotypes and genetic regulation of endometriosis in Indian women: the ECGRI study

Journal:	*BMJ Open*
Manuscript ID	bmjopen-2021-050844.R1
Article Type:	Protocol
Date Submitted by the Author:	17-Jul-2021
Complete List of Authors:	Gajbhiye, Rahul; NIRRH, ;<br>Montgomery, Grant ; The University of Queensland Institute for Molecular Bioscience<br>Pai, Murlidhar; Kasturba Medical College Manipal<br>Phukan, Pranay; Assam Medical College<br>Shekhar, Shahank; All India Institute of Medical Sciences Jodphur<br>Padte , Kedar; 6 Dr Kedar's Maternity, Infertility and Surgical Hospital, Endoscopy and IVF Center, Panaji, India<br>Dasmahapatra , Pramathes; Spectrum Clinic and Endoscopy Research Institute, Kolkata, India<br>John, Bimal; Credence Hospital, Thiruvananthapuram, India<br>Shembekar, Chaitanya ; Omega Hospital, Nagpur, India<br>Bhurke , Aishwarya<br>Bagde, Nilajkumar; All India Institute of Medical Sciences - Raipur<br>Kulkarni, Ketki; Nowrosjee Wadia Maternity Hospital<br>Sardeshpande , Nagendra; Worli Hospital for Women, Mumbai, India<br>Humne, Anil; Government Medical College and Hospital Nagpur<br>Mahobia , Swati; Sai Baba Nursing Home, Raipur, India<br>Shah, Millind; Naval Maternity Endoscopy & Infertility Center, Solapur, India<br>Singh, Uma; King George Medical College<br>Shrivastav, Arti; King George Medical College<br>Mishra, Gita; University of Queensland, Faculty of Medicine, School of Public Health<br>Warty, Neeta; Sanjeevani Diagnostic Center, Mumbai, India<br>Chandra, Sunita; Morpheus Lucknow Fertility Center, Lucknow, India<br>mahale , smita
**Primary Subject Heading**:	Obstetrics and gynaecology
Secondary Subject Heading:	Epidemiology, Genetics and genomics
Keywords:	EPIDEMIOLOGY, GENETICS, GYNAECOLOGY

SCHOLARONE™
Manuscripts

I, the Submitting Author has the right to grant and does grant on behalf of all authors of the Work (as defined in the below author licence), an exclusive licence and/or a non-exclusive licence for contributions from authors who are: i) UK Crown employees; ii) where BMJ has agreed a CC-BY licence shall apply, and/or iii) in accordance with the terms applicable for US Federal Government officers or employees acting as part of their official duties; on a worldwide, perpetual, irrevocable, royalty-free basis to BMJ Publishing Group Ltd ("BMJ") its licensees and where the relevant Journal is co-owned by BMJ to the co-owners of the Journal, to publish the Work in this journal and any other BMJ products and to exploit all rights, as set out in our licence.

The Submitting Author accepts and understands that any supply made under these terms is made by BMJ to the Submitting Author unless you are acting as an employee on behalf of your employer or a postgraduate student of an affiliated institution which is paying any applicable article publishing charge ("APC") for Open Access articles. Where the Submitting Author wishes to make the Work available on an Open Access basis (and intends to pay the relevant APC), the terms of reuse of such Open Access shall be governed by a Creative Commons licence – details of these licences and which Creative Commons licence will apply to this Work are set out in our licence referred to above.

Other than as permitted in any relevant BMJ Author's Self Archiving Policies, I confirm this Work has not been accepted for publication elsewhere, is not being considered for publication elsewhere and does not duplicate material already published. I confirm all authors consent to publication of this Work and authorise the granting of this licence.

For peer review only

Title: Protocol for a case-control study investigating the clinical phenotypes and genetic regulation of endometriosis in Indian women: the ECGRI study

Authors

Rahul K Gajbhiye, MBBS, Ph.D.[1]* (0000-0003-3864-1818),

Grant W Montgomery, Ph.D.[2] (0000-0002-4140-8139),

Muralidhar Pai, M.D.[3] (0000-0002-7942-2383),

Pranay Phukan, M.D.[4],

Shashank Shekhar, M.D.[5] (0000-0003-4612-6533),

Kedar Padte, M.D.[6],

Pramathes DasMahapatra, M.D.[7],

Bimal John, M.D.[8] (0000-0003-4530-2611),

Chaitanya Shembekar, M.D.[9],

Aishwarya V Bhurke, M.Sc.[1] (0000-0002-2000-2076),

Nilajkumar Bagde, M.D.[10] (0000-0003-3035-9906),

Ketki Kulkarni, M.D.[11],

Nagendra Sardeshpande, D.N.B.[12],

Anil Humne, MBBS, D.N.B[13],

Swati Mahobia, M.D[14],

Milind Shah, M.D[15] (0000-0002-5277-0977),

Uma Singh, M.S.[16],

Arti Shrivastav, D.G.O. [16],

Gita D Mishra, Ph.D.[17](0000-0001-9610-5904),

Neeta Warty, M.D.[18],

Sunita Chandra, M.D.[19],

Smita D Mahale, Ph.D [20] (0000-0002-4164-7455).

For peer review only

For peer review only - http://bmjopen.bmj.com/site/about/guidelines.xhtml

Author Affiliations

[1]Indian Council of Medical Research (ICMR)-National Institute for Research in Reproductive Health (NIRRH), Mumbai, India

[2]Institute for Molecular Biosciences, The University of Queensland, St Lucia, Queensland, Australia

[3]Department of Obstetrics and Gynecology, Kasturba Medical College, Manipal, India

[4]Department of Obstetrics and Gynecology, Assam Medical College, Dibrugarh, India

[5]Department of Obstetrics and Gynecology, All India Institute of Medical Sciences, Jodhpur, India

[6]Dr. Kedar's Maternity, Infertility and Surgical Hospital, Endoscopy and IVF Center, Panaji, India

[7]Spectrum Clinic and Endoscopy Research Institute, Kolkata, India

[8]Minimally Invasive Surgery Unit, Credence Hospital, Thiruvananthapuram, India

[9]Omega Hospital, Nagpur, India

[10]Department of Obstetrics and Gynecology, All India Institute of Medical Sciences, Raipur, India

[11]Nowrosjee Wadia Maternity Hospital, Mumbai, India

[12]Worli Hospital for Women, Mumbai, India

[13] Government Medical College, Nagpur, India

[14]Sai Baba Nursing Home, Raipur, India

[15]Naval Maternity Endoscopy & Infertility Center, Solapur, India

[16]King George's Medical University, Lucknow, India

[17]Epidemiology and Biostatistics Division, School of Public Health, The University of Queensland, Herston, Queensland, Australia

[18]Sanjeevani Diagnostic Center, Mumbai, India

For peer review only

[19] Morpheus Lucknow Fertility Center, Lucknow, India

[20] Emeritus Scientist, ICMR-National Institute for Research in Reproductive Health, Mumbai, India

***Corresponding author:** Rahul K Gajbhiye

Scientist D & DBT Wellcome India Alliance Clinical & Public Health Intermediate Fellow

ICMR-National Institute for Research in Reproductive Health,

J. M. Street, Parel, Mumbai, 400012, India.

Tel: +91-22-24192036

Email: gajbhiyer@nirrh.res.in

Word count: 4387

For peer review only

Abstract

Introduction

Endometriosis is one of the common, gynecological disorders associated with chronic pelvic pain and subfertility leading to significant morbidity affecting ~10% of reproductive age women in any population. The presentation and severity of endometriosis subtypes are unknown in Indian women. The etiopathogenesis of endometriosis subtypes and associated risk factors are also largely unknown. Genome-wide association (GWA) studies provide strong evidence for the role of genetic risk factors contributing to endometriosis. However, no studies have investigated the association of the GWAS-identified SNPs with endometriosis risk in the Indian population; therefore, one-sixth of the world's population is not represented in the global genome consortiums on endometriosis. The Endometriosis Clinical and Genetic Research in India (ECGRI) study aims to broaden our understanding of the clinical phenotypes and genetic risks associated with endometriosis in the Indian population.

Methods and analysis

ECGRI is a large-scale, multi-site, case-control study of 2000 endometriosis cases and 2000 hospital controls to be recruited over 4 years at 15 collaborating study sites across India covering representative Indian population covering east/northeast, north, central, west, and southern zones of India. We will utilize the World Endometriosis Research Foundation Endometriosis Phenome and Biobanking Harmonisation Project (WERF-EPHect) data collection instruments for capturing information on clinical, epidemiological, lifestyle, environmental, and surgical factors. WERF-EPHect standard operating procedures will be followed for the collection, processing, and storage of biological samples. The principal analyses will be for main outcome measures of the incidence of endometriosis, disease subtypes, and disease severity determined from the clinical data. This will be followed by GWAS within and across ethnic groups.

For peer review only - http://bmjopen.bmj.com/site/about/guidelines.xhtml

Ethics and dissemination

The study is approved by the Institutional Ethics Committee of ICMR-National Institute for Research in Reproductive Health (NIRRH) and all participating study sites. The study is also approved by the Health Ministry Screening Committee of the Government of India. The results from this study will be actively disseminated through discussions with endometriosis patient groups, conference presentations, and published manuscripts.

Strengths and limitations of the study

- This multicenter study will identify the clinical, epidemiological, environmental, and lifestyle risk factors associated with different endometriosis subtypes in Indian women.
- World Endometriosis Research Foundation Endometriosis Phenome and Biobanking Harmonisation Project standards are followed to ensure high standards of clinical data collection, sample processing, and storage.
- The study extends the discovery of genetic and environmental risk factors for endometriosis to the Indian population adding significantly to our understanding of the causes of this disease.
- The study will lead to the establishment of a bio-repository for endometriosis in India with associated detailed clinical and genomic data.
- The hospital controls comprise female visitors unrelated to the endometriosis cases with no personal or family history of endometriosis; thus, the endometriosis in the control group may not be completely ruled out.

Introduction

Endometriosis is defined as the presence of endometrium-like tissue outside the normal uterine cavity [1]. It is estimated to affect 10% of women of reproductive age in any population and extrapolates to approximately 247 million affected women worldwide and 42 million women in India [2]. India has a population size of more than 1.3 billion and comprises different ethnic

For peer review only

and geographically distinct subpopulations [3]. Women with endometriosis complain of having chronic pelvic pain, dysmenorrhea, dyspareunia, dyschezia, dysuria, fatigue, and infertility [1]. The data on the distribution and clinical presentation of endometriosis in LMICs as well as low-resource settings is extremely limited [4]. The etiology and risk factors associated with endometriosis are not known despite many years of research. Many theories have been proposed such as retrograde menstruation, coelomic metaplasia, the combination of both called the induction theory, lymphatics and vascular metastasis, and the most recent theory of Epigenetics [5,6], genetics [7–10], autoimmunity [11,12], and inflammation [13] are reported to be of significance in the etiology of endometriosis. There is evidence suggesting in utero exposure, menstrual and reproductive factors, anthropometrics, lifestyle, dietary and environmental exposure might play a role in the etiology of endometriosis. However, out of these factors, only earlier age at menarche and shorter menstrual cycle length consistently show an increased risk of endometriosis. Similarly, greater parity and current oral contraceptive use showed decreased risk of endometriosis consistently [14,15]. However, there is no clear understanding of the etiology and pathogenesis and therefore appropriate treatment of the disease is still a major clinical challenge.

The presentation of endometriosis varies from superficial peritoneal lesions of different colors (SUP) to cysts in the ovaries [endometrioma (OMA)], to nodules with a depth of penetration > 5 mm [deeply infiltrating endometriosis (DIE), often associated with fibrosis and adhesions] to the lesions on extra pelvic locations [16]. It is also argued that these subtypes of endometriosis might have different aetiologies [17] and the endometriosis lesions may vary across the life course [16]. There is also an overlap of these endometriosis subtypes in some women and sometimes all of these subtypes are present in the same endometriosis patient. However, there is limited information on the distribution of endometriosis subtypes and associated risks factors.

For peer review only - http://bmjopen.bmj.com/site/about/guidelines.xhtml

The heritability of endometriosis was estimated at ~50% based on Australian twin studies [18]. Similar studies in the Swedish Twin registry reported 47% genetic factors and 53% unique environmental effects in women with endometriosis [19]. Genome-wide association (GWA) studies provide strong evidence for the role of genetic risk factors contributing to endometriosis [4,9,20–22]. The interim results of the ongoing meta-analysis involving 25 global data sets identified 27 genome-wide significant loci [23], accounting for approximately 2.2% of disease variance for endometriosis [16]. Seventy-eight percent (21 loci) had greater effect sizes in stage III/IV disease compared to stage I/II and 4% (1 locus) had greater effect size in stage I/II compared to stage III/IV and 63% (17 loci) had greater effect sizes when restricted to endometriosis with infertility as compared to overall endometriosis cases [23]. These observations emerging from the largest meta-analysis of endometriosis suggest that different subtypes of endometriosis might have specific risk variants.

Previously, we reported a family history of endometriosis with the diagnosis of the disease in either the mother, sister, or grandmother of the proband, with some women reporting multiple affected relatives mainly from West Bengal or Assam regions of India [24]. Additionally, we also observed the increased incidence of deep infiltrating endometriosis in these regions. Endometriosis cases with unusual presentation such as endometriosis-associated endometrioid type of ovarian cancer, cervical endometriosis, endometriosis-associated with didelphys uterus were also reported in Indian women [24]. Possible explanations for differences in incidence and disease presentation in different regions of India include differences in ethnicity and/or environmental factors. Therefore, there exists an unmet need to study distributions and presentation of endometriosis in Indian women and also characterize the different subtypes of endometriosis, superficial peritoneal endometriosis (SUP), ovarian endometrioma (OMA), and deeply infiltrating endometriosis (DIE), and the presentation of the disease in different ethnic sub-populations in India. To date, no studies have investigated the association of the GWAS-

For peer review only - http://bmjopen.bmj.com/site/about/guidelines.xhtml

identified SNPs with endometriosis risk in the Indian population, therefore leaving one-sixth of the world population not represented in the International Endometriosis Genome Consortium (IEGC).

To achieve these goals, we are collecting prospective high-quality, standardized clinical, epidemiological, environmental, and surgical data with corresponding biological samples. Our group has been actively engaged with World Endometriosis Research Foundation's (WERF) Endometriosis Phenome and Biobanking Harmonisation Project (EPHect) for implementing the WERF-EPHect research instruments and also translating the endometriosis patient questionnaires in different Indian languages.

The Endometriosis Clinical and Genetic Research in India (ECGRI) study aims to conduct a large-scale, prospective, case-control study to investigate the clinical phenotypes and genetic risks associated with endometriosis in the Indian population.

For peer review only

Objectives

1. To identify and characterize endometriosis subtypes [superficial peritoneal endometriosis (SUP), cystic ovarian endometriosis or endometrioma (OMA), and deep infiltrating endometriosis (DIE)] in different geographical regions of India
2. To identify the clinical, epidemiological, environmental, lifestyle factors associated with different endometriosis subtypes in India
3. To investigate the genetic risks associated with endometriosis in the Indian population

Outcomes

Primary outcome

We will use the modified WERF-Endometriosis Patient Questionnaire (EPQ), and Surgical Standard Form (SSF) data to data collection investigate the clinical, epidemiological, and environmental, and lifestyle risk factors in women with endometriosis. The GWAS analysis will be carried out to investigate the genetic risk factors in women with endometriosis. At the

end of the study participants recruitment period, that is from August 2023 onwards, the collected data and samples will be analyzed and compared between women with endometriosis and hospital controls recruited at different clinics and different regions of India. The clinical and phenotypic differences in the presentation of endometriosis at different clinics and different regions of India will be determined. The replication in the Indian population of genetic risk factors for endometriosis discovered in European populations will be conducted.

Secondary outcomes

The correlation of clinical and genomic data together with endometriosis subtype status will allow us to discover and replicate genetic risk loci for endometriosis subtypes and help in understanding the pathogenesis of endometriosis and its subtypes. The clinical and genetic findings will be compared against the public databases and functional studies will be carried out in an appropriate models.

Secondary outcomes will be a possible discovery of population-specific common risk factors for endometriosis and if there are significant differences in the clinical presentation of endometriosis in different regions of India. We will conduct secondary analyses, making use of the genetic information to define ethnic subgroups across India, to evaluate whether these differences might result from ethnic or environmental differences between regions.

This stud will also provide an opportunity to add the endometriosis phenotype-genotype data to the International Consortium. Regional data on endometriosis sub-phenotypes, demographic, environmental, reproductive, menstrual, lifestyle factors of endometriosis will be useful in developing the strategies and policies to address the endometriosis-associated morbidity in India and LMICs.

Methods and analysis

Study design and study sites

For peer review only - http://bmjopen.bmj.com/site/about/guidelines.xhtml

This is a multi-site, prospective, case-control study conducted over 5 years beginning in October 2019 and ending in September 2024. It is being conducted at 15 collaborating study sites across India covering representative Indian populations from the north, northeast, east, central, west, and south geographical zones of India (Figure 1). The selection criteria of the study sites were as follows: (1) proven track record of gynecologic research; (2) availability of required research manpower and infrastructure to be able to recruit the required study participants, (3) existing trusted relationship from previous collaborations. Each of these geographical zones where study participants will be recruited has significant genetic diversity which is likely to improve the ability of this research to answer the objectives of the study.

Patient and public involvement

The ECGRI study was conceptualized based on our experience and feedback we received from collaborators and patients during our previous studies [11,12]. The initial cohort of women with endometriosis provided feedback on general workflow, data collection, WERF-EPHect questionnaires.

Samples

After obtaining written informed consent, 15 ml of peripheral blood will be collected by venipuncture by a trained phlebotomist (10 ml in EDTA tube and 5 ml in plain serum tube) from endometriosis cases and controls.

Participants

We plan to recruit up to 2000 women with endometriosis and 2000 hospitals controls. The study participants will be recruited consecutively for 4 years at all the study sites until the desired sample size of 400 endometriosis cases and 400 controls per geographical zone is achieved.

Inclusion criteria

Inclusion Criteria for Endometriosis Cases:

For peer review only - http://bmjopen.bmj.com/site/about/guidelines.xhtml

- Females aged 18- 50 years, visiting clinics at the study sites for benign gynecological conditions will be initially screened and evaluated for their eligibility in the study.
- They should be willing to participate in the study, should be a resident of the geographic region for the collaborating center for a minimum period of one year.
- Eligible females with a surgically confirmed diagnosis of endometriosis on laparoscopy will be recruited as endometriosis cases. Endometriosis will be further confirmed on histology wherever possible.
- The laparoscopic images of the endometriosis lesions will be provided by the clinical collaborators along with the completed WERF-EPHect Standard Surgical Form.
- Endometriosis cases will be sub-classified as superficial peritoneal endometriosis (SUP), cystic ovarian endometriosis or endometrioma (OMA), and deep infiltrating endometriosis (DIE). Endometriosis cases with any additional subtypes/ phenotypic features other than SUP, OMA, and DIE will also be included.
- Women with a family history of endometriosis will also be invited to participate with one proband per family included in the main study. Affected and non-affected relatives of the proband up to a minimum of three generations will be recruited wherever possible. Data of familial endometriosis cases will be analyzed separately from the sporadic endometriosis cases.

Inclusion Criteria for Hospital Controls:

- The appropriate comparison group having the same opportunity to develop endometriosis and representative of the same geographical location as endometriosis cases will be selected from all the collaborating centers.
- These study participants will be the hospital-based controls residing in the geographic region of the collaborating centers for a minimum of one year.

For peer review only

- Female visitors aged 18-50 years, unrelated to the endometriosis cases, who are visiting the collaborating centers during the same period of recruitment of endometriosis cases will be eligible for enrolment as controls.

Exclusion Criteria:

- Female visitors having personal or family history of endometriosis will be excluded from the control group.

Participant enrolment

Screening of cases and controls

The flow chart of study participant recruitment and study processes is represented in Figure 2. Eligible study participants (Endometriosis cases and hospital controls) will be screened and identified by the Clinical Collaborators at each study site. Clinical collaborators are experienced gynecological laparoscopic surgeons having expertise in the diagnosis and management of endometriosis.

Informed Consent

Research staff who have received study-specific training will approach the study participants identified by the Clinical Collaborator. They will provide a Participant Information Sheet (PIS) that includes the purpose and procedures of the study and carefully explain the same to the participants in their local language. They will explain to participants that participation in this study is entirely voluntary and participants are free to withdraw from the study at any point without affecting their medical care and services. A copy of the signed informed consent and PIS will be given to participants in the language they read and understand. Languages include Hindi, Marathi, Malayali, Assamese, Kannada, Bengali, and English. The original signed consent form will be retained at the study site.

Interventions

Non- Clinical Interventions

For peer review only - http://bmjopen.bmj.com/site/about/guidelines.xhtml

WERF-EPHect Endometriosis Patient Questionnaire (Modified)

To determine the differences in endometriosis subtypes, presentation, and disease severity between geographical regions and ethnic groups in India, data will be collected with the WERF-EPHect endometriosis patient questionnaire (EPQ) modified version form [25]. After obtaining the informed consent from the participants, the Research staff will administer the modified WERF-EPHect EPQ under the supervision of Clinical Collaborators for both cases and controls. The modified EPQ is designed to capture phenotype data across the life cycle particularly relevant due to delay in diagnosis. Pain-related questionnaires on an 11-point numerical rating scale (NRS) (0-10) will capture four separate domains of pain (continuous, intermittent, neuropathic, and affective). Information on depression, anxiety, and Health-related Quality of Life will be captured. Menstrual history including age at menarche, menstrual irregularities, last date of menstrual period, frequency, duration of flow, and menstrual cycle length will be recorded. Use of hormonal treatments, length of time, and type of treatment will be obtained. Information on subfertility, age at the start of each pregnancy, type of fertility treatment used for each pregnancy, and pregnancy outcome (if applicable), live births, type of delivery, and pregnancy complications will be captured. A detailed history of medical and surgical treatments will also be obtained. The sociodemographic, personal, and lifestyle data including age, ethnicity, education, anthropometric measurements, history of smoking, alcohol use, and exercise will be captured by modified EPQ. Information on occupation and history of residence in each decade of life will also be captured (Supplementary Table 1).

Standard Surgical Form

The surgical data will be collected by the Clinical Collaborators using WERF-EPHect Standard Surgical Form [SSF] [26]. EPHect SSF consists of 2 parts designed to capture all relevant visual information of the endometriosis lesion phenotype and the surgical treatment

For peer review only - http://bmjopen.bmj.com/site/about/guidelines.xhtml

including type, duration of the procedure and requires approximately 1-3 minutes to complete the SSF. The first part of SSF includes detailed information on clinical covariates and requires detailed information related to the current menstrual cycle, current hormonal treatment, history of previous surgery for endometriosis, ultrasound or MRI findings before the procedure. The second part focuses on detailed visual information on intraoperative findings such as extent, exact location, and color of endometriotic lesions, with a specific focus on endometrioma dimensions and endometriotic nodules. It also includes information on the endometriosis fertility index. Based on the EPHect SSF data, the phenotype characterization of endometriosis will be carried out into superficial peritoneal endometriosis (SUP), cystic ovarian endometriosis, or endometrioma (OMA), and deep infiltrating endometriosis (DIE). From the EPHect SSF data, rAFS (Revised American Fertility Society) score will be calculated and endometriosis cases will be grouped into Stage I/II or Stage III/IV.

Familial Endometriosis

The clinical data of female relatives of the proband will be collected as per the WERF-EPHect and the family history of the proband will be documented through pedigree analysis. This will include both affected and non-affected relatives of the proband up to a minimum of three generations wherever possible.

Laparoscopic images documentation

The collaborators (gynecological laparoscopic surgeons) at each study site will obtain laparoscopic images of the different endometriotic lesions and adhesions. The soft copies of the labeled laparoscopic images will be uploaded to the ECGRI electronic database. The

Power calculations

Over 4 years, the study team will recruit a total of 2000 endometriosis cases and 2000 hospital controls across five geographical zones of India. To detect an odds ratio of ≥ 2 with

For peer review only - http://bmjopen.bmj.com/site/about/guidelines.xhtml

a 95% confidence interval and 90% power with 10% missing or incomplete WERF-EPHect data collection forms, the required sample size per zone = 360. In each geographical zone, four hundred endometriosis cases and four hundred hospital controls will be recruited.

To replicate results from the European GWAS [21,23] (P=0.05/27 or P<0.0018) and test all 27 "hits" using either average effect size or effect size for the smallest effect (Risk allele with frequency 0.3 and genotype relative risk 1.22 with 80 % power), the total sample size required is as follows: Endometriosis Cases (n=1250), Hospital Controls (n=1250). For conducting discovery GWAS in the Indian population (effect size to detect at $P<5x10^{-8}$), a total sample size of 2000 endometriosis and 2000 hospital controls will be recruited. To conduct replication GWAS for novel risk loci in Indian women with endometriosis an Independent set of endometriosis cases and controls will be recruited based on discovery GWAS results.

Electronic database

A dedicated website is developed for the project (https://ecgri.in). Electronic data collection software is developed for online data entry (http://app.ecgri.in/login). The Project assistants will enter the data electronically from the paper copies of the WERF-EPHect Endometriosis Patient Questionnaire and Standard Surgical Forms. The online electronic data collection software (http://app.ecgri.in/login) is hosted on a secure server at the Biomedical Informatics Division of ICMR- National Institute for Research in Reproductive Health (NIRRH), Mumbai. The central database will allow online, real-time data entry with the option of online editing. Project assistants and Clinical Collaborators will have access to data entry, data editing only for their respective centers and will not have any access to the whole database.

Research Team Training and Quality Controls

The research staff has received study-specific training including recruitment of study participants, obtaining informed consent, counseling the study participants, data collection as

For peer review only - http://bmjopen.bmj.com/site/about/guidelines.xhtml

per WERF-EPHect EPQ, sample collection, processing, storage, and transport as per WERF-EPHect Standard Operating Procedures (SOPs). Intense Training was provided to the research staff, Clinical Collaborators on selection and recruitment of study participants, data collection as per WERF-EPHect Questionnaire and Standard Surgical Forms (SSF), sample collection, processing, and transportation as per WERF-EPHect SOPs, and online data entry. A copy of the training manual is provided to all study sites. A Data analyst will coordinate with Clinical Collaborators and research staff for timely entry of the data and transportation of samples. The Data Analyst will report to RG at ICMR-NIRRH.

RD will provide training to the Data Analyst on data collection, quality control, data entry and online editing, data analysis, development and maintenance of databases at ICMR-NIRRH. The research staff will report to their respective Clinical Collaborators at participating study sites. The Data Analyst will supervise, conduct regular quality control checks and clean the data to identify and rectify the errors by comparing the hard and soft copies of data collection instruments. RG will coordinate with Clinical Collaborators for recruitment of study participants, data collection, data entry, blood sample collection-processing, and transport as per the WERF-EPHect research instruments. Video meetings will be organized with all Collaborating Centres, a research team at ICMR-NIRRH to review the progress and ensure adherence to protocol. A detailed checklist is provided to the collaborating centers to ensure adherence to the protocol. There will be weekly auditing of data collection and data entry for each site by the Clinical Collaborator to ensure that the protocols are followed and data entry is done promptly as soon as the data is collected. The data analyst will coordinate with the collaborating centers every week through email/video/teleconference. There will be detailed regular monitoring of a random sample of individuals collected at each site. The centralized database at NIRRH, Mumbai will be checked and updated every week to provide regular reports from each study site and across the study on the number of cases and controls collected

For peer review only - http://bmjopen.bmj.com/site/about/guidelines.xhtml

with additional information on the completion of all parts of the protocol for each individual. RG will provide regular feedback to each and all sites on issues of compliance every month. The hard copies of the original documents (WERF-EPHect instruments- modified EPQ, SSF) will be transported to the ICMR-NIRRH for cross-checking the data entry. These data forms will be stored as per the National ethical guidelines for biomedical and health research involving human participants [27].

Collection, processing, and storage of blood samples

The standard operating procedures (SOPs) have been established and are being implemented in RG's laboratory as per the WERF-EPHect collection protocol [28]. The cases and controls will be recruited simultaneously during the study period. After obtaining informed consent, 15 ml peripheral blood (10 ml in EDTA tube and 5 ml in plain serum tube) will be collected in an appropriate setting. The resulting plasma/serum will be stored in aliquots (500µl) prior to freezing in - 80º C freezer with power back up. Collected samples will be separated into its 3 derivatives (Serum, Plasma, and Buffy coat). All the details of the collection will be recorded on the EPHect Biospecimens form.

Sample Processing, Genotyping, and Quality Control

Genomic DNA will be extracted from buffy coats using the QIAamp DNA Blood Midi kit (Qiagen) using the manufacturer's instructions. The concentration of each DNA sample will be determined. The DNA samples that pass minimum requirements for sample quality will be sent to the commercial facility for genome-wide genotype analysis using the appropriate genotyping platform (Illumina/Afffimatrix) specific to Asian Indian populations. Standard genotype quality control procedures will be performed using the software PLINK. SNPs with a high missing rate (>5%), low minor allele frequency ($<1x10^{-4}$), and with Hardy-Weinburg Equilibrium p<10-6 will be excluded along with samples with low call rates (<95%), extreme heterozygosity, high genetic relatedness, and any ancestry outliers.

For peer review only - http://bmjopen.bmj.com/site/about/guidelines.xhtml

Standard methods for quality control and genetic associations will be adopted as reported earlier [21]. Following quality control, genotypes will be imputed to the 1000 Genomes Phase 3 reference panel using the Michigan Imputation Server. Endometriosis cases with a family history either in grandmother, mother, or sister will be recruited for enrichment sampling. The genotype data of familial endometriosis cases will be analyzed separately. Exome sequencing will be carried out for the identification of the genes associated with familial endometriosis.

Data analysis plan

We will use SPSS, GraphPad Prism, R, and plink software for analysis. The principal analyses will be for main outcome measures of the incidence of endometriosis, disease subtypes, and disease severity determined from the clinical data. We will conduct conditional logistic regression to analyze effects of geographical region and ethnicity (defined from genetic data) adjusting for age, recruitment clinic, and other confounding variables that may be identified from the initial inspection of the data. Disease presentation and endometriosis severity between regions and ethnic groups in India will be analyzed. Co-morbidity and environmental exposures between regions will be compared and correlated with disease severity and distribution of lesions. Genotype data will be used to define ethnicity for the phenotypic analysis. Association between previously identified endometriosis risk loci and endometriosis status in our Indian cohort will be analyzed on imputed genotypes using SNPTEST v2. An additive logistic regression model will be used to test association whilst controlling for population stratification by including age and the top 5 principle components as covariates in the model. A study-wide significance threshold of $p<0.05/n$ will be applied where n is the number of loci tested. Subsequently, we will perform a genome-wide association analysis using SNPTEST to estimate adjusted odds ratios and corresponding 95% CI between each available SNP and endometriosis case/control status. The sub-

For peer review only

For peer review only - http://bmjopen.bmj.com/site/about/guidelines.xhtml

phenotype association analysis will be performed for rAFS stage, site, geographical regions (North, East, North East, South, West, and Central India), infertility, and associated comorbidities (reproductive, metabolic, inflammatory, autoimmune disorders, COVID-19, etc.).

Functional studies aimed at delineating the causal genetic variants and biological mechanisms underlying the observed statistical associations with endometriosis risk will be carried based on the results of the discovery GWAS. Meta-analysis in collaboration with IEGC and other Genomic groups will be explored.

Ethics and dissemination

The study has ethics approval and will be monitored by the Institutional Ethics Committee (IEC) of the study sites and ICMR-NIRRH. The study protocol will also be monitored by the Health Ministry Screening Committee (HMSC) of the Government of India. Periodic progress reports are required to be submitted to HMSC, IEC of ICMR-NIRRH, and IECs of study sites. A periodic progress report will be submitted to the funding agency.

Plans for data management, curation, storage, and confidentiality

Clinical and genomic datasets from Indian women with and without endometriosis will be captured in a central database (http://app.ecgri.in/login), maintained, and regularly updated at ICMR-NIRRH. The database is developed on a robust platform including the ability to allow online, real-time data entry. Demographic data will be kept in separate tables and records identifying the study participants will be kept confidential. Data entry and data access will be independently controlled and identifiable data held behind secure firewalls according to the ICMR- National Guidelines for Biomedical and Health Research Involving Human Participants [27]. Data collected from different sites will be held separately and only specific individuals within a research team will be able to access and edit data. Where possible, data will be captured using online tools, including the clinical data in the WERF-EPHect

For peer review only - http://bmjopen.bmj.com/site/about/guidelines.xhtml

instruments. Data collected from paper questionnaires will be entered and verified independently. The quality of the clinical and genomic data will be regularly monitored. The genotype data will be stored in a separate computer with sufficient memory space and backup data will be stored in hard disks. The clinical and genomic data will be carefully linked by individual identification numbers to maintain the confidentiality of the participants and the key will be held by RG at ICMR-NIRRH.

Publications

Results will be presented and discussed with endometriosis patient groups in India and elsewhere through Endometriosis Society India (ESI) and World Endometriosis Society (WES). RG and GWM are currently actively interacting and communicating with the World Endometriosis Society (WES) and World Endometriosis Research Foundation (WERF). The research findings from the study will be published in peer-reviewed, high-impact factor journals. The findings will be disseminated at national and international conferences.

Study status

The present study was conceptualized and designed in 2019. The first participant was recruited in January 2020, the study is ongoing and desired sample size will be achieved by September 2023. The baseline characteristics of endometriosis cases and hospital controls recruited till 31st January 2021 are shown in Table 1. The distributions of isolated and overlap of endometriosis sub-phenotypes in the initial cohort of 155 women with endometriosis are presented in Figure 3.

Discussion

This large-scale study has been designed to investigate endometriosis sub-phenotypes and risk factors and conduct a GWAS in Indian women with endometriosis representative of different geographical regions. ECGRI study will follow WERF-EPHect criteria to ensure standardization and reproducibility for high-quality clinical and genomic datasets. The sample

For peer review only - http://bmjopen.bmj.com/site/about/guidelines.xhtml

size of the present study is calculated taking into consideration the expected effect sizes for genetic risk factors, the possibility for discovery of population-specific risk factors, selection criteria for cases and controls, and differences in allelic frequencies among studied populations [29].

The study will lead to the establishment of endometriosis bio-repository of serum, plasma, DNA samples as well as the establishment of a clinical, genomic database of endometriosis in India. The results of this study will improve the understanding of the pathogenesis of endometriosis and its subtypes and also form the basis to join the global network of International Consortium on Endometriosis and contribute to improving patient healthcare in India and for patients everywhere.

Funding

This work was supported by the DBT Wellcome Trust India Alliance Fellowship (Grant no. IA/CPHI/18/1/503933) awarded to Rahul K Gajbhiye. The sponsor was not involved in the study design, in the writing of the report, and in the decision to submit the paper for publication.

Author contributions

RG conceived the study, designed the final study protocol, provided the domain knowledge expertise, coordinated with all site investigators, obtained the ethics approval, drafted the manuscript, revised and is the principal investigator of the grant; GWM helped in the design of the final study protocol, provided domain knowledge expertise, contributed in revision of the manuscript draft. MP, PP, SS, KP, PDM, BJ, CS, NB, KK, NS, AH, SM, MS, US, AS, NW, SC provided clinical inputs, coordinated ethics approval, recruited study participants, obtained consents from the study participants, collected data, samples and involved in quality control and data interpretation. AB contributed to the development and piloting of electronic database, conducted quality controls, data analysis, and interpretation. GDM provided the biostatistical and epidemiological support for the study protocol and data analysis. SDM

For peer review only

contributed to the development of an electronic database and provided technical and administrative support for the study. All authors read and approved the final manuscript.

Competing interests

None declared.

Ethics approval:

Ethics approvals have been granted by ICMR-NIRRH (D/ICEC/Sci-87/91/2019; D/ICEC/Sci-165/170/2020) and Institutional Ethics Committees of study sites (ECR/146/Inst/KA/2013/RR-19; AMC/EC/1304; AIIMS/IEC/2019-20/884; CH/IEC/19/004; 734/IEC/-AIIMSRPR/2019; IEC-NWMH/AP/2019/033-V1; 1320/IEC-AIIMSRPR/2020; /839EC/Pharmac/GMC/NGP/; ECR/262/Inst/UP/2013/RR-19). The study has been approved by the Health Ministry Screening Committee (HMSC) of the Government of India. (HMSC No.: 2019-7620).

Data sharing

All data relevant to the study are included in the article or uploaded as supplementary information.

Acknowledgments

The authors thank the members of the ECGRI study group. Dr. Shailaja Patil, Dr. Manisha Shembekar, Dr. Parul Sharma Saoji, Jishna RP, Nimisha Bhuyan, Akhila S., Kiran Kharsodiya, Ashwini Patel, Merlin Pious, Aarti Kushwaha, Sudeshna Bakshi, Akshatha Shetty, Nadha Abdul Razack, Neha Yadav are sincerely acknowledged for assistance in data collection. Mr. Ram Barai is acknowledged for assistance in ECGRI electronic database development. Dr. Yogesh Kalkonde and Dr. Rajesh Dikshit are acknowledged for critical inputs on the study design. Data collection was facilitated by and conducted in compliance with the World Endometriosis Research Foundation Endometriosis Phenome and Biobanking Harmonisation Project (WERF EPHect). This work was supported by the DBT Wellcome Trust India Alliance Fellowship [Grant no. IA/CPHI/18/1/503933] awarded to Rahul K Gajbhiye.

For peer review only

References

1 Zondervan KT, Becker CM, Missmer SA. Endometriosis. *N Engl J Med* 2020;**382**:1244–56.

2 Population ages 15-64 (% of total population) | Data. https://data.worldbank.org/indicator/SP.POP.1564.TO.ZS (accessed 3 Feb 2021).

3 Population, total - India | Data. https://data.worldbank.org/indicator/SP.POP.TOTL?locations=IN (accessed 25 Jan 2021).

4 Rogers PAW, Adamson GD, Al-Jefout M, *et al.* Research Priorities for Endometriosis: Recommendations From a Global Consortium of Investigators in Endometriosis. *Reprod Sci* 2017;**24**:202–26.

5 Grimstad FW, Decherney A. A Review of the Epigenetic Contributions to Endometriosis: *Clin Obstet Gynecol* 2017;**60**:467–76.

6 Koninckx PR, Ussia A, Adamyan L, *et al.* Pathogenesis of endometriosis: the genetic/epigenetic theory. *Fertil Steril* 2019;**111**:327–40.

7 Rahmioglu N, Macgregor S, Drong AW, *et al.* Genome-wide enrichment analysis between endometriosis and obesity-related traits reveals novel susceptibility loci. *Hum Mol Genet* 2015;**24**:1185–99.

8 Fung JN, Mortlock S, Girling JE, *et al.* Genetic regulation of disease risk and endometrial gene expression highlights potential target genes for endometriosis and polycystic ovarian syndrome. *Sci Rep* 2018;**8**:11424–11424.

9 Gajbhiye R, Fung JN, Montgomery GW. Complex genetics of female fertility. *NPJ Genom Med* 2018;**3**:29–29.

10 Montgomery GW, Giudice LC. New Lessons about Endometriosis — Somatic Mutations and Disease Heterogeneity. *N Engl J Med* 2017;**376**:1881–2.

11 Gajbhiye R, Bendigeri T, Ghuge A, *et al.* Panel of Autoimmune Markers for Noninvasive Diagnosis of Minimal–Mild Endometriosis: A Multicenter Study. *Reprod Sci* 2017;**24**:413–20.

12 Gajbhiye R, Sonawani A, Khan S, *et al.* Identification and validation of novel serum markers for early diagnosis of endometriosis. *Hum Reprod* 2012;**27**:408–17.

13 Gajbhiye R, McKinnon B, Mortlock S, *et al.* Genetic Variation at Chromosome 2q13 and Its Potential Influence on Endometriosis Susceptibility Through Effects on the IL-1 Family. *Reprod Sci* 2018;:1933719118768688.

14 Farland LV, Shah DK, Kvaskoff M, *et al.* Epidemiological and Clinical Risk Factors for Endometriosis. In: D'Hooghe T, ed. *Biomarkers for Endometriosis: State of the Art*. Cham: : Springer International Publishing 2017. 95–121.

For peer review only - http://bmjopen.bmj.com/site/about/guidelines.xhtml

15 Shafrir AL, Farland LV, Shah DK, *et al.* Risk for and consequences of endometriosis: A critical epidemiologic review. *Best Pract Res Clin Obstet Gynaecol* 2018;**51**:1–15.

16 Zondervan KT, Becker CM, Missmer SA. Endometriosis. *N Engl J Med* 2020;**382**:1244–56.

17 Zondervan KT, Becker CM, Koga K, *et al.* Endometriosis. *Nat Rev Dis Primers* 2018;**4**:9.

18 Treloar SA, O'Connor DT, O'Connor VM, *et al.* Genetic influences on endometriosis in an Australian twin sample. sueT@qimr.edu.au. *Fertil Steril* 1999;**71**:701–10.

19 Saha R, Pettersson HJ, Svedberg P, *et al.* Heritability of endometriosis. *Fertil Steril* 2015;**104**:947–52.

20 Fung JN, Montgomery GW. Genetics of endometriosis: State of the art on genetic risk factors for endometriosis. *Best Pract Res Clin Obstet Gynaecol* 2018;**50**:61–71.

21 Sapkota Y, Steinthorsdottir V, Morris AP, *et al.* Meta-analysis identifies five novel loci associated with endometriosis highlighting key genes involved in hormone metabolism. *Nat Commun* 2017;**8**:15539.

22 Montgomery GW, Mortlock S, Giudice LC. Should Genetics Now Be Considered the Pre-eminent Etiologic Factor in Endometriosis? *JMIG* 2020;**27**:280–6.

23 Nilufer R, Karina B, Paraskevi C, *et al.* Large-scale genome-wide association meta-analysis of endometriosis reveals 13 novel loci and genetically-associated comorbidity with other pain conditions. *bioRxiv* 2018;:406967.

24 Bendigeri T, Warty N, Sawant R, *et al.* Endometriosis: Clinical Experience of 500 Patients from India. *Indian Pract* 2015;**68**:34-40–40.

25 Vitonis AF, Vincent K, Rahmioglu N, *et al.* World Endometriosis Research Foundation Endometriosis Phenome and Biobanking Harmonization Project: II. Clinical and covariate phenotype data collection in endometriosis research. *Fertil Steril* 2014;**102**:1223–32.

26 Becker CM, Laufer MR, Stratton P, *et al.* World Endometriosis Research Foundation Endometriosis Phenome and Biobanking Harmonisation Project: I. Surgical phenotype data collection in endometriosis research. *Fertility and Sterility* 2014;**102**:1213–22.

27 Mathur R, Swaminathan S. National ethical guidelines for biomedical & health research involving human participants, 2017: A commentary. *IJMR* 2018;**148**:279.

28 Rahmioglu N, Fassbender A, Vitonis AF, *et al.* World Endometriosis Research Foundation Endometriosis Phenome and Biobanking Harmonization Project: III. Fluid biospecimen collection, processing, and storage in endometriosis research. *Fertil Steril* 2014;**102**:1233–43.

29. Cardoso JV, Perini JA, Machado DE, et al. Systematic review of genome-wide association studies on susceptibility to endometriosis. European Journal of Obstetrics &

For peer review only - http://bmjopen.bmj.com/site/about/guidelines.xhtml

For peer review only

Gynecology and Reproductive Biology 2020;**255**:74–82. doi:10.1016/j.ejogrb.2020.10.017

Figure Legends

Figure 1: Study sites of ECGRI across different geographic regions of India

Figure 2: Flow chart of ECGRI study participant recruitment and study processes.

Abbreviations: WERF-EPHect, World Endometriosis Research Foundation – Endometriosis Phenome and Biobanking Harmonisation Project; EPQ, Endometriosis Patient Questionnaire; SSF, Standard Surgical Form; SOP, Standard Operating Processes; QC, Quality Control; ICMR-NIRRH, Indian Council of Medical Research-National Institute for Research in Reproductive Health.

Figure 3: Distribution of isolated and overlap of endometriosis subtypes in Indian women.

Abbreviations: SUP, Superficial Peritoneal Endometriosis; OMA, Ovarian Endometriosis/ Endometrioma; DIE, Deep Infiltrating Endometriosis

For peer review only

Table 1: Sociodemographic, clinical and surgical characteristics of Indian women with endometriosis cases and hospital controls

Characteristics		Cases	Controls	P value
**Demographics**		(n=159)	(n=148)	
Age (years), Mean ± SD		32.5 ± 7.1	30.3 ± 7.2	0.550*
BMI (kg/m^2), Mean ± SD		24.0 ± 4.4	24.5 ± 4.2	0.889*
BMI (kg/m^2) categories	Underweight (≤18.4)	10 (6.5)	5 (3.5)	0.653†
	Normal (18.5 – 22.9)	56 (36.4)	50 (35)	
	Overweight (23 – 24.9)	26 (16.9)	27 (18.9)	
	Obese (≥25)	62 (40.3)	61 (42.7)	
Education level	Up to Higher Secondary	45 (28.3)	62 (41.9)	0.016‡
	Graduate and above	114 (71.7)	86 (58.1)	
Employment	Government/ Private job	45 (28.5)	78 (52.7)	<0.001†
	Self employed	17 (10.8)	19 (12.8)	
	Unemployed	85 (53.8)	31 (20.9)	
	Student	11 (7)	20 (13.5)	
Annual family income (□)	≤100,000	65 (41.1)	65 (44.5)	0.013†
	>100,000 - 500,000	60 (38)	68 (46.6)	
	≥500,000	33 (20.9)	13 (8.9)	
Marital status**	Married	133 (83.6)	87 (58.8)	<0.001†
	Unmarried	23 (14.5)	58 (39.2)	
**Clinical**				
Age at Menarche (in years)	≤12	55 (34.6)	38 (25.7)	0.106‡
	>12	104 (65.4)	110 (74.3)	

For peer review only - http://bmjopen.bmj.com/site/about/guidelines.xhtml

Regularity of menses	Regular	121 (82.9)	128 (94.1)	0.005‡
	Irregular	25 (17.1)	8 (5.9)	
Menstrual Bleeding		n= 144	n= 134	
Duration (in days), Mean ± SD		4.3 ± 1.7	4.2 ± 2.5	0.435*
Menstrual Cycle Length (in days)	24-31	107 (74.3)	114 (85.1)	0.037‡
	>31	37 (25.7)	20 (14.9)	
Menstrual Blood Loss (in ml), Mean ± SD	At Heaviest (n=146)	44.8 ± 12.8	41.7 ± 10.9	<0.001*
	On average (n=136)	31.1 ± 14.8	26.9 ± 14.3	0.538*
Pain symptoms				
Dysmenorrhea	Yes	143 (89.9)	84 (56.8)	<0.001‡
	No	16 (10.1)	64 (43.2)	
Dyspareunia	Yes (n=62)	47 (36.7)	15 (15.8)	0.001‡
	No (n=161)	81 (63.3)	80 (84.2)	
**Surgical**				
Endometriosis subtypes dominant# (n=155)	SUP	33 (21.3)	-	-
	OMA	98 (63.2)	-	-
	DIE	22 (14.2)	-	-
	Scar endometriosis	2 (1.3)	-	-
Endometriosis severity (n=152)	rAFS stage I	31 (20)	-	-
	rAFS stage II	16 (10.3)	-	-
	rAFS stage III	33 (21.3)	-	-
	rAFS stage IV	72 (46.5)	-	-
Recurrent endometriosis (n=15)	Once	12 (80)	-	-
	Twice	2 (13.3)	-	-

For peer review only - http://bmjopen.bmj.com/site/about/guidelines.xhtml

Thrice	1 (6.7)	-	-
EFI score (range 0-10) (n=154), Mean ± SD	5.6 ± 2.2	-	-

Abbreviations: BMI, Body Mass Index; rAFS, Revised American Fertility Society; EFI, Endometriosis Fertility Index

Data presented as n (%), unless specified otherwise.

*t-test

†Pearson Chi-square test

‡Fisher's Exact test

# The disease was categorized as follows: isolated sub-phenotypes (SUP or OMA or DIE) and overlap of sub-phenotypes. All the women with endometriosis irrespective of the overlap were combined and further classified as dominant SUP or OMA or DIE based on the dominance (severity of the disease and size of lesions) of the superficial endometriotic lesions, or adhesions or endometrioma or deep endometriotic lesions within these sub-phenotypes.

**3 endometriosis cases and 3 controls were either widowed or divorced. In the current cohort, 74 women were infertile, among which 61 (91%) cases and 3 (42.9%) controls had primary infertility, while 6 (9%) cases and 4 (57.1%) controls had secondary infertility. However, due to the small sample size, the groups were not comparable.

For peer review only

For peer review only - http://bmjopen.bmj.com/site/about/guidelines.xhtml

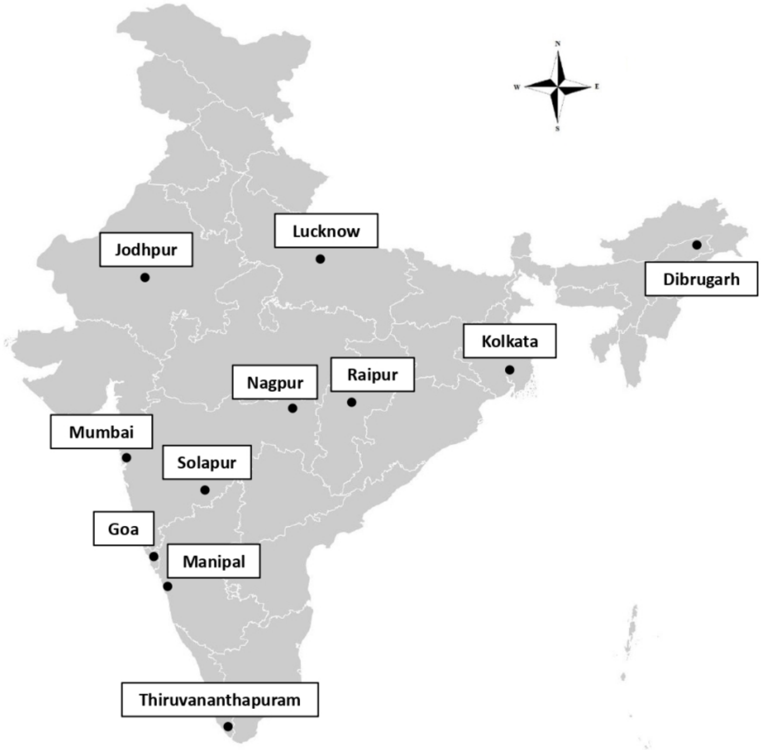

State	Study sites	State	Study sites
Assam	Assam Medical College, Dibrugarh	Maharashtra	Worli Hospital for Women, Mumbai; Nowrosjee Wadia Maternity Hospital, Mumbai; Naval Maternity Endoscopy & Infertility Center, Mumbai
Uttar Pradesh	King George's Medical University, Lucknow; Morpheus Lucknow Fertility Center, Lucknow		
Rajasthan	All India Institute of Medical Sciences (AIIMS), Jodhpur		
West Bengal	Spectrum Clinic and Endoscopy Research Institute, Kolkata	Maharashtra	Naval Maternity Endoscopy & Infertility Center, Solapur
Chhattisgarh	AIIMS, Raipur; Sai Baba Nursing Home, Raipur	Karnataka	Kasturba Medical College, Manipal
Maharashtra	Government Medical College, Nagpur; Omega Hospital, Nagpur	Kerala	Credence Hospital, Thiruvananthapuram

Study sites of ECGRI across different geographic regions of India

95x137mm (300 x 300 DPI)

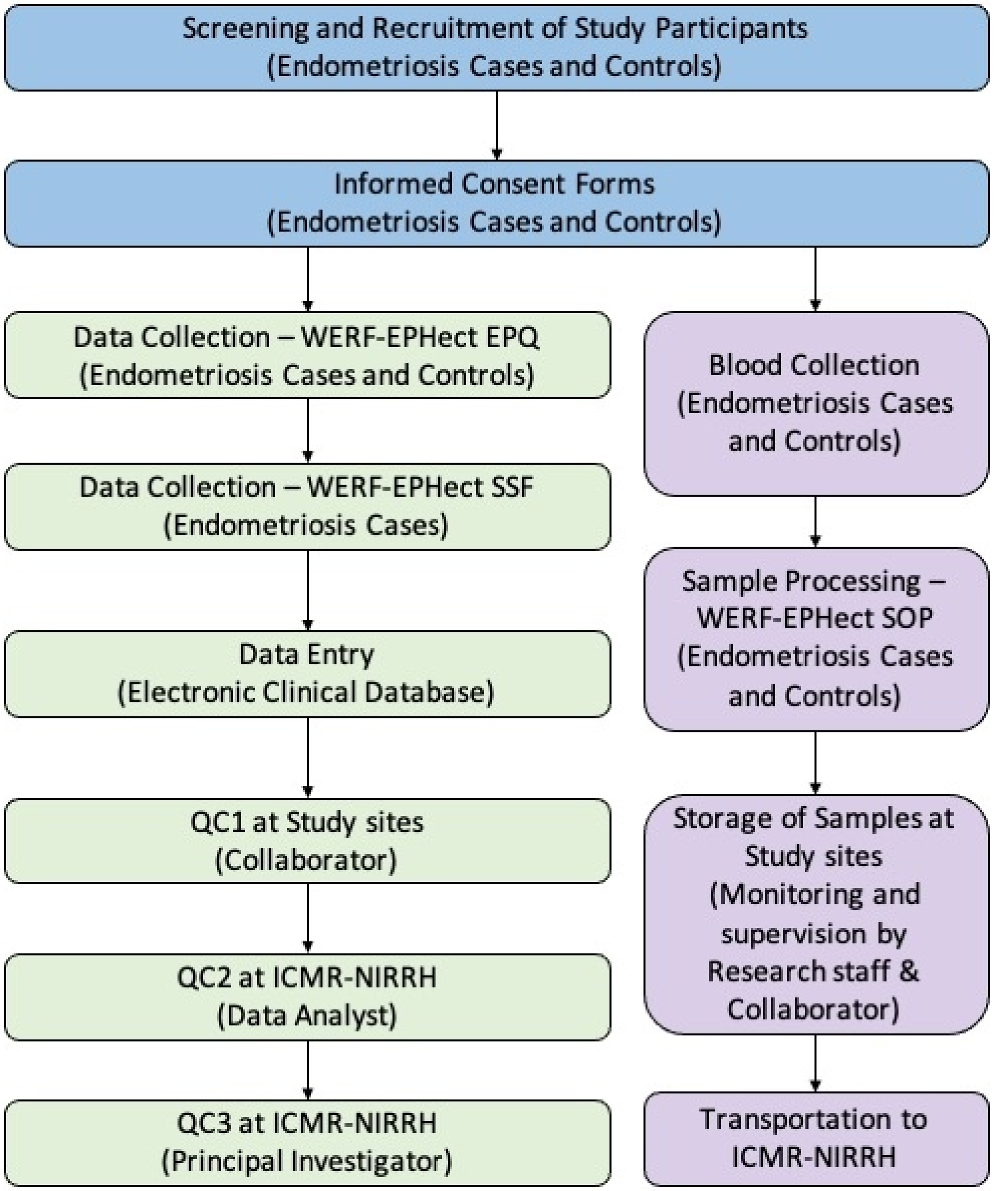

Flow chart of ECGRI study participant recruitment and study processes.
Abbreviations: WERF-EPHect, World Endometriosis Research Foundation – Endometriosis Phenome and Biobanking Harmonisation Project; EPQ, Endometriosis Patient Questionnaire; SSF, Standard Surgical Form; SOP, Standard Operating Processes; QC, Quality Control; ICMR-NIRRH, Indian Council of Medical Research – National Institute for Research in Reproductive Health.

41x50mm (300 x 300 DPI)

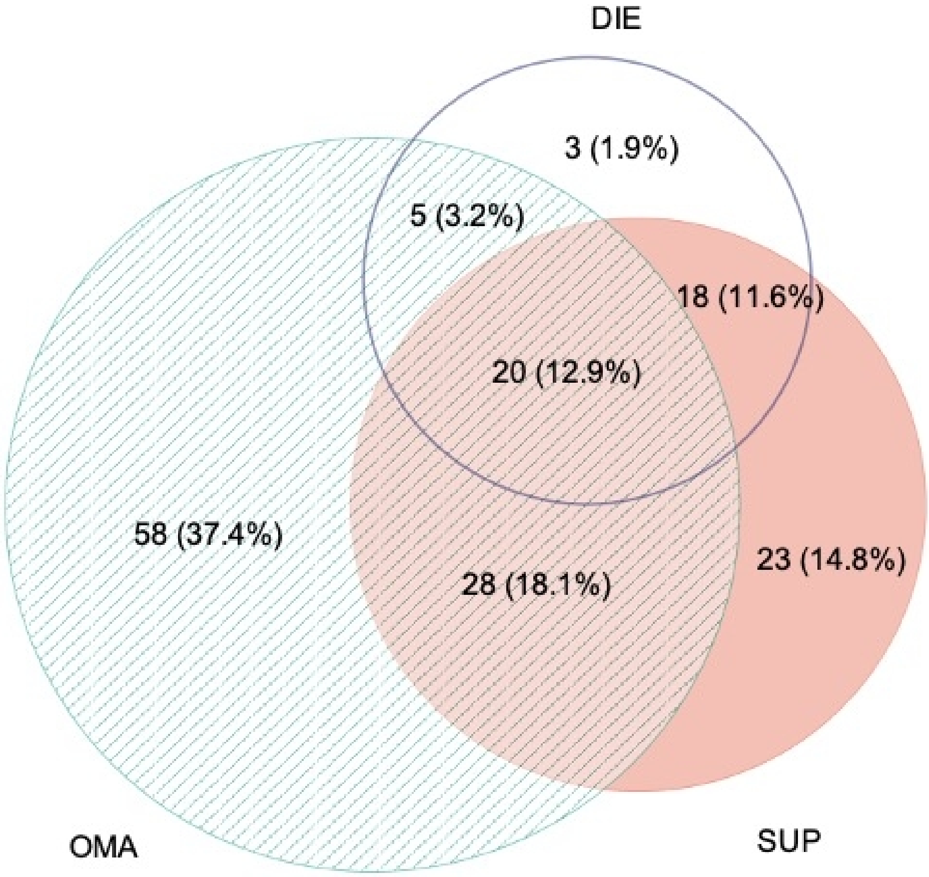

Figure 3: Distribution of isolated and overlap of endometriosis subtypes in Indian women. Abbreviations: SUP, Superficial Peritoneal Endometriosis; OMA, Ovarian Endometriosis/ Endometrioma; DIE, Deep Infiltrating Endometriosis

43x40mm (300 x 300 DPI)

Supplementary Table 1: Clinical, epidemiological, reproductive, lifestyle and environmental characteristics captured using modified Endometriosis Patient Questionnaire (EPQ)

Characteristics	Variables
**Menstrual and Reproductive factors**	Age at menarche, menstrual cycle length, menstrual volume, dysmenorrhea, current oral contraceptive use, hormones, history of infertility
**Pregnancy and Fertility**	Age at each pregnancy, parity, fertility treatment, outcome of pregnancy, history of miscarriage, ectopic pregnancy, vaginal delivery, caesarean section, breast feeding, natural pregnancy/ ART, complications related to pregnancy- GDM, PIH, Preterm birth
**Health-related Quality of life**	Thoughts and feelings associated with pain, severity grading scale (0-10), Life time details - pelvic pain, pain during or after vaginal intercourse, treatment for pain, gastrointestinal and urinary symptoms etc.
**Socio-demographic, personal and lifestyle factors**	Geographic location, age, educational level, marital status, age at marriage, employment, family income, height, weight, BMI, waist -to- hip ratio, ethnicity, cigarette smoking, alcohol use, caffeine intake, regular exercise, night shift work, natural hair color, eye color, skin color, body shape
**Medical and surgical**	History of associated diseases- cancer, autoimmune diseases<br>endometriosis or chronic pelvic pain history- personal and family (if known), details of medical and surgical treatment
**Environmental factors**	Residence in each decade of life, pollution exposure, smoking environment,<br>sun exposure, occupational history, pesticide exposure, any other relevant information

Abbreviations: ART, Assisted Reproductive Technology; GDM, Gestational Diabetes Mellitus; PIH, Pregnancy-induced hypertension; BMI, Body mass index

For peer review only

STROBE 2007 (v4) Statement—Checklist of items that should be included in reports of *case-control studies*

Section/Topic	Item #	Recommendation	Reported on page #
**Title and abstract**	1	(*a*) Indicate the study's design with a commonly used term in the title or the abstract	1,4
		(*b*) Provide in the abstract an informative and balanced summary of what was done and what was found	4,5
**Introduction**			
Background/rationale	2	Explain the scientific background and rationale for the investigation being reported	6-8
Objectives	3	State specific objectives, including any prespecified hypotheses	8
**Methods**			
Study design	4	Present key elements of study design early in the paper	10
Setting	5	Describe the setting, locations, and relevant dates, including periods of recruitment, exposure, follow-up, and data collection	10
Participants	6	(*a*) Give the eligibility criteria, and the sources and methods of case ascertainment and control selection. Give the rationale for the choice of cases and controls	11,12
		(*b*) For matched studies, give matching criteria and the number of controls per case	11, 15
Variables	7	Clearly define all outcomes, exposures, predictors, potential confounders, and effect modifiers. Give diagnostic criteria, if applicable	13-15
Data sources/ measurement	8*	For each variable of interest, give sources of data and details of methods of assessment (measurement). Describe comparability of assessment methods if there is more than one group	18
Bias	9	Describe any efforts to address potential sources of bias	18,19
Study size	10	Explain how the study size was arrived at	15
Quantitative variables	11	Explain how quantitative variables were handled in the analyses. If applicable, describe which groupings were chosen and why	28
Statistical methods	12	(*a*) Describe all statistical methods, including those used to control for confounding	18,19
		(*b*) Describe any methods used to examine subgroups and interactions	18,19
		(*c*) Explain how missing data were addressed	NA
		(*d*) If applicable, explain how matching of cases and controls was addressed	11,12
		(*e*) Describe any sensitivity analyses	Not applicable
**Results**			

For peer review only

Participants	13*	(a) Report numbers of individuals at each stage of study—eg numbers potentially eligible, examined for eligibility, confirmed eligible, included in the study, completing follow-up, and analysed	20
		(b) Give reasons for non-participation at each stage	Nil
		(c) Consider use of a flow diagram	Figure 2
Descriptive data	14*	(a) Give characteristics of study participants (eg demographic, clinical, social) and information on exposures and potential confounders	26,27,28
		(b) Indicate number of participants with missing data for each variable of interest	Nil
Outcome data	15*	Report numbers in each exposure category, or summary measures of exposure	Not applicable
Main results	16	(*a*) Give unadjusted estimates and, if applicable, confounder-adjusted estimates and their precision (eg, 95% confidence interval). Make clear which confounders were adjusted for and why they were included	Not applicable
		(*b*) Report category boundaries when continuous variables were categorized	26,27,28
		(*c*) If relevant, consider translating estimates of relative risk into absolute risk for a meaningful time period	Not applicable
Other analyses	17	Report other analyses done—eg analyses of subgroups and interactions, and sensitivity analyses	Nil
**Discussion**			
Key results	18	Summarise key results with reference to study objectives	21
Limitations	19	Discuss limitations of the study, taking into account sources of potential bias or imprecision. Discuss both direction and magnitude of any potential bias	5
Interpretation	20	Give a cautious overall interpretation of results considering objectives, limitations, multiplicity of analyses, results from similar studies, and other relevant evidence	Not applicable
Generalisability	21	Discuss the generalisability (external validity) of the study results	21
**Other information**			
Funding	22	Give the source of funding and the role of the funders for the present study and, if applicable, for the original study on which the present article is based	21

*Give information separately for cases and controls in case-control studies and, if applicable, for exposed and unexposed groups in cohort and cross-sectional studies.

Note: An Explanation and Elaboration article discusses each checklist item and gives methodological background and published examples of transparent reporting. The STROBE checklist is best used in conjunction with this article (freely available on the Web sites of PLoS Medicine at http://www.plosmedicine.org/, Annals of Internal Medicine at http://www.annals.org/, and Epidemiology at http://www.epidem.com/). Information on the STROBE Initiative is available at www.strobe-statement.org.

For peer review only

BMJ Open

Protocol for a case-control study investigating the clinical phenotypes and genetic regulation of endometriosis in Indian women: the ECGRI study

Journal:	*BMJ Open*
Manuscript ID	bmjopen-2021-050844.R2
Article Type:	Protocol
Date Submitted by the Author:	22-Jul-2021
Complete List of Authors:	Gajbhiye, Rahul; NIRRH, ;<br>Montgomery, Grant ; The University of Queensland Institute for Molecular Bioscience<br>Pai, Murlidhar; Kasturba Medical College Manipal<br>Phukan, Pranay; Assam Medical College<br>Shekhar, Shahank; All India Institute of Medical Sciences Jodphur<br>Padte , Kedar; 6 Dr Kedar’s Maternity, Infertility and Surgical Hospital, Endoscopy and IVF Center, Panaji, India<br>Dasmahapatra , Pramathes; Spectrum Clinic and Endoscopy Research Institute, Kolkata, India<br>John, Bimal; Credence Hospital, Thiruvananthapuram, India<br>Shembekar, Chaitanya ; Omega Hospital, Nagpur, India<br>Bhurke , Aishwarya<br>Bagde, Nilajkumar; All India Institute of Medical Sciences - Raipur<br>Kulkarni, Ketki; Nowrosjee Wadia Maternity Hospital<br>Sardeshpande , Nagendra; Worli Hospital for Women, Mumbai, India<br>Humne, Anil; Government Medical College and Hospital Nagpur<br>Mahobia , Swati; Sai Baba Nursing Home, Raipur, India<br>Shah, Millind; Naval Maternity Endoscopy & Infertility Center, Solapur, India<br>Singh, Uma; King George Medical College<br>Shrivastav, Arti; King George Medical College<br>Mishra, Gita; University of Queensland, Faculty of Medicine, School of Public Health<br>Warty, Neeta; Sanjeevani Diagnostic Center, Mumbai, India<br>Chandra, Sunita; Morpheus Lucknow Fertility Center, Lucknow, India<br>mahale , smita
**Primary Subject Heading**:	Obstetrics and gynaecology
Secondary Subject Heading:	Epidemiology, Genetics and genomics
Keywords:	EPIDEMIOLOGY, GENETICS, GYNAECOLOGY

SCHOLARONE™
Manuscripts

I, the Submitting Author has the right to grant and does grant on behalf of all authors of the Work (as defined in the below author licence), an exclusive licence and/or a non-exclusive licence for contributions from authors who are: i) UK Crown employees; ii) where BMJ has agreed a CC-BY licence shall apply, and/or iii) in accordance with the terms applicable for US Federal Government officers or employees acting as part of their official duties; on a worldwide, perpetual, irrevocable, royalty-free basis to BMJ Publishing Group Ltd ("BMJ") its licensees and where the relevant Journal is co-owned by BMJ to the co-owners of the Journal, to publish the Work in this journal and any other BMJ products and to exploit all rights, as set out in our licence.

The Submitting Author accepts and understands that any supply made under these terms is made by BMJ to the Submitting Author unless you are acting as an employee on behalf of your employer or a postgraduate student of an affiliated institution which is paying any applicable article publishing charge ("APC") for Open Access articles. Where the Submitting Author wishes to make the Work available on an Open Access basis (and intends to pay the relevant APC), the terms of reuse of such Open Access shall be governed by a Creative Commons licence – details of these licences and which Creative Commons licence will apply to this Work are set out in our licence referred to above.

Other than as permitted in any relevant BMJ Author's Self Archiving Policies, I confirm this Work has not been accepted for publication elsewhere, is not being considered for publication elsewhere and does not duplicate material already published. I confirm all authors consent to publication of this Work and authorise the granting of this licence.

For peer review only

Title: Protocol for a case-control study investigating the clinical phenotypes and genetic regulation of endometriosis in Indian women: the ECGRI study

Authors

Rahul K Gajbhiye, MBBS, Ph.D.[1]* (0000-0003-3864-1818),

Grant W Montgomery, Ph.D.[2] (0000-0002-4140-8139),

Muralidhar Pai, M.D.[3] (0000-0002-7942-2383),

Pranay Phukan, M.D.[4],

Shashank Shekhar, M.D.[5] (0000-0003-4612-6533),

Kedar Padte, M.D.[6],

Pramathes DasMahapatra, M.D.[7],

Bimal John, M.D.[8] (0000-0003-4530-2611),

Chaitanya Shembekar, M.D.[9],

Aishwarya V Bhurke, M.Sc.[1] (0000-0002-2000-2076),

Nilajkumar Bagde, M.D.[10] (0000-0003-3035-9906),

Ketki Kulkarni, M.D.[11],

Nagendra Sardeshpande, D.N.B.[12],

Anil Humne, MBBS, D.N. B[13],

Swati Mahobia, M. D[14],

Millind Shah, M. D[15] (0000-0002-5277-0977),

Uma Singh, M.S.[16],

Arti Shrivastav, D.G.O. [16],

Gita D Mishra, Ph.D.[17](0000-0001-9610-5904),

Neeta Warty, M.D.[18],

Sunita Chandra, M.D.[19],

Smita D Mahale, Ph.D. [20] (0000-0002-4164-7455)

For peer review only

For peer review only - http://bmjopen.bmj.com/site/about/guidelines.xhtml

Author Affiliations

[1]Indian Council of Medical Research (ICMR)-National Institute for Research in Reproductive Health (NIRRH), Mumbai, India

[2]Institute for Molecular Biosciences, The University of Queensland, St Lucia, Queensland, Australia

[3]Department of Obstetrics and Gynecology, Kasturba Medical College, Manipal, India

[4]Department of Obstetrics and Gynecology, Assam Medical College, Dibrugarh, India

[5]Department of Obstetrics and Gynecology, All India Institute of Medical Sciences, Jodhpur, India

[6]Dr. Kedar's Maternity, Infertility and Surgical Hospital, Endoscopy and IVF Center, Panaji, India

[7]Spectrum Clinic and Endoscopy Research Institute, Kolkata, India

[8]Minimally Invasive Surgery Unit, Credence Hospital, Thiruvananthapuram, India

[9]Omega Hospital, Nagpur, India

[10]Department of Obstetrics and Gynecology, All India Institute of Medical Sciences, Raipur, India

[11]Nowrosjee Wadia Maternity Hospital, Mumbai, India

[12]Worli Hospital for Women, Mumbai, India

[13] Government Medical College, Nagpur, India

[14]Sai Baba Nursing Home, Raipur, India

[15]Naval Maternity Endoscopy & Infertility Center, Solapur, India

[16]King George's Medical University, Lucknow, India

[17]Epidemiology and Biostatistics Division, School of Public Health, The University of Queensland, Herston, Queensland, Australia

[18]Sanjeevani Diagnostic Center, Mumbai, India

For peer review only

For peer review only - http://bmjopen.bmj.com/site/about/guidelines.xhtml

[19] Morpheus Lucknow Fertility Center, Lucknow, India

[20] Emeritus Scientist, ICMR-National Institute for Research in Reproductive Health, Mumbai, India

***Corresponding author:** Rahul K Gajbhiye

Scientist D & DBT Wellcome India Alliance Clinical & Public Health Intermediate Fellow

ICMR-National Institute for Research in Reproductive Health,

J. M. Street, Parel, Mumbai, 400012, India.

Tel: +91-22-24192036

Email: gajbhiyer@nirrh.res.in

For peer review only

For peer review only - http://bmjopen.bmj.com/site/about/guidelines.xhtml

Abstract

Introduction

Endometriosis is one of the common, gynaecological disorders associated with chronic pelvic pain and subfertility affecting ~10% of reproductive age women. The clinical presentation, etiopathogenesis of endometriosis subtypes and associated risk factors are largely unknown. Genome-wide association (GWA) studies provide strong evidence for the role of genetic risk factors contributing to endometriosis. However, no studies have investigated the association of the GWAS-identified SNPs with endometriosis risk in the Indian population; therefore, one-sixth of the world's population is not represented in the global genome consortiums on endometriosis. The Endometriosis Clinical and Genetic Research in India (ECGRI) study aims to broaden our understanding of the clinical phenotypes and genetic risks associated with endometriosis.

Methods and analysis

ECGRI is a large-scale, multi-site, case-control study of 2000 endometriosis cases and 2000 hospital controls to be recruited over 4 years at 15 collaborating study sites across India covering representative Indian population covering east/northeast, north, central, west, and southern zones of India. We will utilize the World Endometriosis Research Foundation Endometriosis Phenome and Biobanking Harmonisation Project (WERF-EPHect) data collection instruments for capturing information on clinical, epidemiological, lifestyle, environmental, and surgical factors. WERF-EPHect standard operating procedures will be followed for the collection, processing, and storage of biological samples. The principal analyses will be for main outcome measures of the incidence of endometriosis, disease subtypes, and disease severity determined from the clinical data. This will be followed by GWAS within and across ethnic groups.

Ethics and dissemination

For peer review only

The study is approved by the Institutional Ethics Committee of ICMR-National Institute for Research in Reproductive Health (NIRRH) and all participating study sites. The study is also approved by the Health Ministry Screening Committee of the Government of India. The results from this study will be actively disseminated through discussions with endometriosis patient groups, conference presentations, and published manuscripts.

Strengths and limitations of the study

- This multicenter study will identify the clinical, epidemiological, environmental, and lifestyle risk factors associated with different endometriosis subtypes in Indian women.
- World Endometriosis Research Foundation Endometriosis Phenome and Biobanking Harmonisation Project standards are followed to ensure high standards of clinical data collection, sample processing, and storage.
- The study extends the discovery of genetic and environmental risk factors for endometriosis to the Indian population adding significantly to our understanding of the causes of this disease.
- The study will lead to the establishment of a bio-repository for endometriosis in India with associated detailed clinical and genomic data.
- The hospital controls comprise female visitors unrelated to the endometriosis cases with no personal or family history of endometriosis; thus, the endometriosis in the control group may not be completely ruled out.

Introduction

Endometriosis is defined as the presence of endometrium-like tissue outside the normal uterine cavity [1]. It is estimated to affect 10% of women of reproductive age in any population and extrapolates to approximately 247 million affected women worldwide and 42 million women in India [2]. India has a population size of more than 1.3 billion and comprises different ethnic and geographically distinct subpopulations [3]. Women with endometriosis complain of having

For peer review only - http://bmjopen.bmj.com/site/about/guidelines.xhtml

chronic pelvic pain, dysmenorrhea, dyspareunia, dyschezia, dysuria, fatigue, and infertility [1]. The data on the distribution and clinical presentation of endometriosis in LMICs as well as low-resource settings is extremely limited [4]. The etiology and risk factors associated with endometriosis are not known despite many years of research. Many theories have been proposed such as retrograde menstruation, coelomic metaplasia, the combination of both called the induction theory, lymphatics and vascular metastasis, and the most recent theory of Epigenetics [5,6], genetics [7–10], autoimmunity [11,12], and inflammation [13] are reported to be of significance in the etiology of endometriosis. There is evidence suggesting in utero exposure, menstrual and reproductive factors, anthropometrics, lifestyle, dietary and environmental exposure might play a role in the etiology of endometriosis. However, out of these factors, only earlier age at menarche and shorter menstrual cycle length consistently showed an increased risk of endometriosis. Similarly, greater parity and current oral contraceptive use showed decreased risk of endometriosis consistently [14,15]. However, there is no clear understanding of the etiology and pathogenesis of endometriosis and therefore appropriate treatment of the disease is still a major clinical challenge.

The presentation of endometriosis varies from superficial peritoneal lesions of different colours (SUP) to cysts in the ovaries [endometrioma (OMA)], to nodules with a depth of penetration > 5 mm [deeply infiltrating endometriosis (DIE), often associated with fibrosis and adhesions] to the lesions on extra pelvic locations [1]. It is also argued that these subtypes of endometriosis might have different aetiologies [16] and the endometriosis lesions may vary across the life course [1]. There is also an overlap of these endometriosis subtypes in some women and sometimes all of these subtypes are present in the same endometriosis patient. However, there is limited information on the distribution of endometriosis subtypes and associated risks factors.

For peer review only - http://bmjopen.bmj.com/site/about/guidelines.xhtml

The heritability of endometriosis was estimated at ~50% based on Australian twin studies [17]. Similar studies in the Swedish Twin registry reported 47% genetic factors and 53% unique environmental effects in women with endometriosis [18]. Genome-wide association (GWA) studies provide strong evidence for the role of genetic risk factors contributing to endometriosis [4,9,19–21]. The interim results of the ongoing meta-analysis involving 25 global data sets identified 27 genome-wide significant loci [22], accounting for approximately 2.2% of disease variance for endometriosis [1]. Seventy-eight percent (21 loci) had greater effect sizes in stage III/IV disease compared to stage I/II and 4% (1 locus) had greater effect size in stage I/II compared to stage III/IV and 63% (17 loci) had greater effect sizes when restricted to endometriosis with infertility as compared to overall endometriosis cases [22]. These observations emerging from the largest meta-analysis of endometriosis suggest that different subtypes of endometriosis might have specific risk variants.

Previously, we reported a family history of endometriosis with the diagnosis of the disease in either the mother, sister, or grandmother of the proband, with some women reporting multiple affected relatives mainly from West Bengal or Assam regions of India [23]. Additionally, we also observed the increased incidence of deep infiltrating endometriosis in these regions. Endometriosis cases with unusual presentation such as endometriosis-associated endometrioid type of ovarian cancer, cervical endometriosis, endometriosis-associated with didelphys uterus were also reported in Indian women [23]. Possible explanations for differences in incidence and disease presentation in different regions of India include differences in ethnicity and/or environmental factors. Therefore, there exists an unmet need to study distributions and presentation of endometriosis in Indian women and also characterize the different subtypes of endometriosis, superficial peritoneal endometriosis (SUP), ovarian endometrioma (OMA), and deeply infiltrating endometriosis (DIE), and the presentation of the disease in different ethnic sub-populations in India. To date, no studies have investigated the association of the GWAS-

For peer review only

For peer review only - http://bmjopen.bmj.com/site/about/guidelines.xhtml

identified SNPs with endometriosis risk in the Indian population, therefore leaving one-sixth of the world population not represented in the International Endometriosis Genome Consortium (IEGC).

To achieve these goals, we are collecting prospective high-quality, standardized clinical, epidemiological, environmental, and surgical data with corresponding biological samples. Our group has been actively engaged with World Endometriosis Research Foundation's (WERF) Endometriosis Phenome and Biobanking Harmonisation Project (EPHect) for implementing the WERF-EPHect research instruments and also translating the endometriosis patient questionnaires in different Indian languages.

The Endometriosis Clinical and Genetic Research in India (ECGRI) study aims to conduct a large-scale, prospective, case-control study to investigate the clinical phenotypes and genetic risks associated with endometriosis in the Indian population.

Objectives

1. To identify and characterize endometriosis subtypes [superficial peritoneal endometriosis (SUP), cystic ovarian endometriosis or endometrioma (OMA), and deep infiltrating endometriosis (DIE)] in different geographical regions of India
2. To identify the clinical, epidemiological, environmental, lifestyle factors associated with different endometriosis subtypes in India
3. To investigate the genetic risks associated with endometriosis in the Indian population

Methods and analysis

Study design and study sites

This is a multi-site, prospective, case-control study conducted over 5 years beginning in October 2019 and ending in September 2024. It is being conducted at 15 collaborating study sites across India covering representative Indian populations from the north, northeast, east, central, west, and south geographical zones of India (Figure 1). The selection criteria of the

For peer review only - http://bmjopen.bmj.com/site/about/guidelines.xhtml

study sites were as follows: (1) proven track record of gynaecologic research; (2) availability of required research manpower and infrastructure to be able to recruit the required study participants, (3) existing trusted relationship from previous collaborations. Each of these geographical zones where study participants will be recruited has significant genetic diversity which is likely to improve the ability of this research to answer the objectives of the study.

Patient and public involvement

The ECGRI study was conceptualized based on our experience and feedback we received from collaborators and patients during our previous studies [11,12]. The initial cohort of women with endometriosis provided feedback on general workflow, data collection, WERF-EPHect questionnaires.

Samples

After obtaining written informed consent, 15 ml of peripheral blood will be collected by venepuncture by a trained phlebotomist (10 ml in EDTA tube and 5 ml in plain serum tube) from endometriosis cases and controls.

Participants

We plan to recruit up to 2000 women with endometriosis and 2000 hospitals controls. The study participants will be recruited consecutively for 4 years at all the study sites until the desired sample size of 400 endometriosis cases and 400 controls per geographical zone is achieved.

Inclusion criteria

Inclusion criteria for endometriosis cases:

- Females aged 18- 50 years, visiting clinics at the study sites for benign gynecological conditions will be initially screened and evaluated for their eligibility in the study.
- They should be willing to participate in the study, should be a resident of the geographic region for the collaborating center for a minimum period of one year.

For peer review only - http://bmjopen.bmj.com/site/about/guidelines.xhtml

- Eligible females with a surgically confirmed diagnosis of endometriosis on laparoscopy will be recruited as endometriosis cases. Endometriosis will be further confirmed on histology wherever possible.
- The laparoscopic images of the endometriosis lesions will be provided by the clinical collaborators along with the completed WERF-EPHect Standard Surgical Form.
- Endometriosis cases will be sub-classified as superficial peritoneal endometriosis (SUP), cystic ovarian endometriosis or endometrioma (OMA), and deep infiltrating endometriosis (DIE). Endometriosis cases with any additional subtypes/ phenotypic features other than SUP, OMA, and DIE will also be included.
- Women with a family history of endometriosis will also be invited to participate with one proband per family included in the main study. Affected and non-affected relatives of the proband up to a minimum of three generations will be recruited wherever possible. Data of familial endometriosis cases will be analyzed separately from the sporadic endometriosis cases.

Inclusion criteria for hospital controls:

- The appropriate comparison group having the same opportunity to develop endometriosis and representative of the same geographical location as endometriosis cases will be selected from all the collaborating centers.
- These study participants will be the hospital-based controls residing in the geographic region of the collaborating centers for a minimum of one year.
- Female visitors aged 18-50 years, unrelated to the endometriosis cases, who are visiting the collaborating centers during the same period of recruitment of endometriosis cases will be eligible for enrolment as controls.

Exclusion Criteria:

For peer review only - http://bmjopen.bmj.com/site/about/guidelines.xhtml

- Female visitors having personal or family history of endometriosis will be excluded from the control group.

Participant enrolment

Screening of cases and controls

The flow chart of study participant recruitment and study processes is represented in Figure 2. Eligible study participants (endometriosis cases and hospital controls) will be screened and identified by the Clinical Collaborators at each study site. Clinical collaborators are experienced gynaecological laparoscopic surgeons having expertise in the diagnosis and management of endometriosis.

Informed consent

Research staff who have received study-specific training will approach the study participants identified by the Clinical Collaborator. They will provide a Participant Information Sheet (PIS) that includes the purpose and procedures of the study and carefully explain the same to the participants in their local language. They will explain to participants that participation in this study is entirely voluntary and participants are free to withdraw from the study at any point without affecting their medical care and services. A copy of the signed informed consent and PIS will be given to participants in the language they read and understand. Languages include Hindi, Marathi, Malayali, Assamese, Kannada, Bengali, and English. The original signed consent form will be retained at the study site.

Interventions

Non- clinical interventions

WERF-EPHect Endometriosis Patient Questionnaire (Modified)

To determine the differences in endometriosis subtypes, presentation, and disease severity between geographical regions and ethnic groups in India, data will be collected with the WERF-EPHect endometriosis patient questionnaire (EPQ) modified version form [24]. After

For peer review only - http://bmjopen.bmj.com/site/about/guidelines.xhtml

obtaining the informed consent from the participants, the Research staff will administer the modified WERF-EPHect EPQ under the supervision of Clinical Collaborators for both cases and controls. The modified EPQ is designed to capture phenotype data across the life cycle particularly relevant due to delay in diagnosis. Pain-related questionnaires on an 11-point numerical rating scale (NRS) (0-10) will capture four separate domains of pain (continuous, intermittent, neuropathic, and affective). Information on depression, anxiety, and Health-related Quality of Life will be captured. Menstrual history including age at menarche, menstrual irregularities, last date of menstrual period, frequency, duration of flow, and menstrual cycle length will be recorded. Use of hormonal treatments, length of time, and type of treatment will be obtained. Information on subfertility, age at the start of each pregnancy, type of fertility treatment used for each pregnancy, and pregnancy outcome (if applicable), live births, type of delivery, and pregnancy complications will be captured. A detailed history of medical and surgical treatments will also be obtained. The sociodemographic, personal, and lifestyle data including age, ethnicity, education, anthropometric measurements, history of smoking, alcohol use, and exercise will be captured by modified EPQ. Information on occupation and history of residence in each decade of life will also be captured (Supplementary Table 1).

Standard Surgical Form

The surgical data will be collected by the Clinical Collaborators using WERF-EPHect Standard Surgical Form [SSF] [25]. EPHect SSF consists of 2 parts designed to capture all relevant visual information of the endometriosis lesion phenotype and the surgical treatment including type, duration of the procedure and requires approximately 1-3 minutes to complete the SSF. The first part of SSF includes detailed information on clinical covariates and requires detailed information related to the current menstrual cycle, current hormonal treatment, history of previous surgery for endometriosis, ultrasound or MRI findings before

For peer review only - http://bmjopen.bmj.com/site/about/guidelines.xhtml

the procedure. The second part focuses on detailed visual information on intraoperative findings such as extent, exact location, and colour of endometriotic lesions, with a specific focus on endometrioma dimensions and endometriotic nodules. It also includes information on the endometriosis fertility index. Based on the EPHect SSF data, the phenotype characterization of endometriosis will be carried out into superficial peritoneal endometriosis (SUP), cystic ovarian endometriosis, or endometrioma (OMA), and deep infiltrating endometriosis (DIE). From the EPHect SSF data, rAFS (Revised American Fertility Society) score will be calculated and endometriosis cases will be grouped into Stage I/II or Stage III/IV.

Familial endometriosis

The clinical data of female relatives of the proband will be collected as per the WERF-EPHect and the family history of the proband will be documented through pedigree analysis. This will include both affected and non-affected relatives of the proband up to a minimum of three generations wherever possible.

Laparoscopic images documentation

The collaborators (gynaecological laparoscopic surgeons) at each study site will obtain laparoscopic images of the different endometriotic lesions and adhesions. The soft copies of the labelled laparoscopic images will be uploaded to the ECGRI electronic database.

Power calculations

Over 4 years, the study team will recruit a total of 2000 endometriosis cases and 2000 hospital controls across five geographical zones of India. To detect an odds ratio of ≥ 2 with a 95% confidence interval and 90% power with 10% missing or incomplete WERF-EPHect data collection forms, the required sample size per zone =360. In each geographical zone, four hundred endometriosis cases and four hundred hospital controls will be recruited.

For peer review only - http://bmjopen.bmj.com/site/about/guidelines.xhtml

To replicate results from the European GWAS [20,22] (p = 0.05/27 or p<0.0018) and test all 27 "hits" using either average effect size or effect size for the smallest effect (Risk allele with frequency 0.3 and genotype relative risk 1.22 with 80 % power), the total sample size required is as follows: Endometriosis Cases (n=1250), Hospital Controls (n=1250). For conducting discovery GWAS in the Indian population (effect size to detect at $p < 5\times10^{-8}$), a total sample size of 2000 endometriosis and 2000 hospital controls will be recruited. To conduct replication GWAS for novel risk loci in Indian women with endometriosis an independent set of endometriosis cases and controls will be recruited based on discovery GWAS results. The sample size of the present study is calculated taking into consideration the expected effect sizes for genetic risk factors, the possibility for discovery of population-specific risk factors, selection criteria for cases and controls, and differences in allelic frequencies among studied populations [26].

Electronic database

A dedicated website is developed for the project (https://ecgri.in). Electronic data collection software is developed for online data entry (http://app.ecgri.in/login). The Project assistants will enter the data electronically from the paper copies of the WERF-EPHect Endometriosis Patient Questionnaire and Standard Surgical Forms. The online electronic data collection software (http://app.ecgri.in/login) is hosted on a secure server at the Biomedical Informatics Division of ICMR- National Institute for Research in Reproductive Health (NIRRH), Mumbai. The central database will allow online, real-time data entry with the option of online editing. Project assistants and Clinical Collaborators will have access to data entry, data editing only for their respective centers and will not have any access to the whole database.

Research team training and quality controls

The research staff has received study-specific training including recruitment of study participants, obtaining informed consent, counseling the study participants, data collection as

For peer review only - http://bmjopen.bmj.com/site/about/guidelines.xhtml

per WERF-EPHect EPQ, sample collection, processing, storage, and transport as per WERF-EPHect Standard Operating Procedures (SOPs). Intense Training was provided to the research staff, Clinical Collaborators on selection and recruitment of study participants, data collection as per WERF-EPHect Questionnaire and Standard Surgical Forms (SSF), sample collection, processing, and transportation as per WERF-EPHect SOPs, and online data entry. A copy of the training manual is provided to all study sites. A Data analyst will coordinate with Clinical Collaborators and research staff for timely entry of the data and transportation of samples. The Data Analyst will report to RG at ICMR-NIRRH.

RD will provide training to the Data Analyst on data collection, quality control, data entry and online editing, data analysis, development and maintenance of databases at ICMR-NIRRH. The research staff will report to their respective Clinical Collaborators at participating study sites. The Data Analyst will supervise, conduct regular quality control checks and clean the data to identify and rectify the errors by comparing the hard and soft copies of data collection instruments. RG will coordinate with Clinical Collaborators for recruitment of study participants, data collection, data entry, blood sample collection-processing, and transport as per the WERF-EPHect research instruments. Video meetings will be organized with all Collaborating Centres, a research team at ICMR-NIRRH to review the progress and ensure adherence to protocol. A detailed checklist is provided to the collaborating centers to ensure adherence to the protocol. There will be weekly auditing of data collection and data entry for each site by the Clinical Collaborator to ensure that the protocols are followed and data entry is done promptly as soon as the data is collected. The data analyst will coordinate with the collaborating centers every week through email/video/teleconference. There will be detailed regular monitoring of a random sample of individuals collected at each site. The centralized database at NIRRH, Mumbai will be checked and updated every week to provide regular reports from each study site and across the study on the number of cases and controls collected

For peer review only

For peer review only - http://bmjopen.bmj.com/site/about/guidelines.xhtml

with additional information on the completion of all parts of the protocol for each individual. RG will provide regular feedback to each and all sites on issues of compliance every month. The hard copies of the original documents (WERF-EPHect instruments- modified EPQ, SSF) will be transported to the ICMR-NIRRH for cross-checking the data entry. These data forms will be stored as per the National ethical guidelines for biomedical and health research involving human participants [27].

Collection, processing, and storage of blood samples

The standard operating procedures (SOPs) have been established and are being implemented in RG's laboratory as per the WERF-EPHect collection protocol [28]. The cases and controls will be recruited simultaneously during the study period. After obtaining informed consent, 15 ml peripheral blood (10 ml in EDTA tube and 5 ml in plain serum tube) will be collected in an appropriate setting. The resulting plasma/serum will be stored in aliquots (500µl) prior to freezing in - 80º C freezer with power back up. Collected samples will be separated into its 3 derivatives (Serum, Plasma, and Buffy coat). All the details of the collection will be recorded on the EPHect Biospecimens form.

Sample processing, genotyping, and quality control

Genomic DNA will be extracted from buffy coats using the QIAamp DNA Blood Midi kit (Qiagen) using the manufacturer's instructions. The concentration of each DNA sample will be determined. The DNA samples that pass minimum requirements for sample quality will be sent to the commercial facility for genome-wide genotype analysis using the appropriate genotyping platform (Illumina/Afffimatrix) specific to Asian Indian populations. Standard genotype quality control procedures will be performed using the software PLINK. SNPs with a high missing rate (>5%), low minor allele frequency (<1x10^{-4}), and with Hardy-Weinberg Equilibrium p<10-6 will be excluded along with samples with low call rates (<95%), extreme heterozygosity, high genetic relatedness, and any ancestry outliers.

For peer review only - http://bmjopen.bmj.com/site/about/guidelines.xhtml

Standard methods for quality control and genetic associations will be adopted as reported earlier [20]. Following quality control, genotypes will be imputed to the 1000 Genomes Phase 3 reference panel using the Michigan Imputation Server. Endometriosis cases with a family history either in grandmother, mother, or sister will be recruited for enrichment sampling. The genotype data of familial endometriosis cases will be analysed separately. Exome sequencing will be carried out for the identification of the genes associated with familial endometriosis.

Outcomes

Primary outcome

We will use the modified WERF-Endometriosis Patient Questionnaire (EPQ), and Surgical Standard Form (SSF) data to data collection investigate the clinical, epidemiological, and environmental, and lifestyle risk factors in women with endometriosis. The GWAS analysis will be carried out to investigate the genetic risk factors in women with endometriosis. At the end of the study participant's recruitment period, that is from August 2023 onwards, the collected data and samples will be analysed and compared between women with endometriosis and hospital controls recruited at different clinics and different regions of India. The clinical and phenotypic differences in the presentation of endometriosis at different clinics and different regions of India will be determined. The replication in the Indian population of genetic risk factors for endometriosis discovered in European populations will be conducted.

Secondary outcomes

The correlation of clinical and genomic data together with endometriosis subtype status will allow us to discover and replicate genetic risk loci for endometriosis subtypes and help in understanding the pathogenesis of endometriosis and its subtypes. The clinical and genetic

For peer review only - http://bmjopen.bmj.com/site/about/guidelines.xhtml

findings will be compared against the public databases and functional studies will be carried out in an appropriate model.

Secondary outcomes will be a possible discovery of population-specific common risk factors for endometriosis and if there are significant differences in the clinical presentation of endometriosis in different regions of India. We will conduct secondary analyses, making use of the genetic information to define ethnic subgroups across India, to evaluate whether these differences might result from ethnic or environmental differences between regions.

This study will also provide an opportunity to add the endometriosis phenotype-genotype data to the International Consortium. Regional data on endometriosis sub-phenotypes, demographic, environmental, reproductive, menstrual, lifestyle factors of endometriosis will be useful in developing the strategies and policies to address the endometriosis-associated morbidity in India and LMICs.

Data analysis plan

We will use SPSS, Graph Pad Prism, R, and plink software for analysis. The principal analyses will be for main outcome measures of the incidence of endometriosis, disease subtypes, and disease severity determined from the clinical data. We will conduct conditional logistic regression to analyse effects of geographical region and ethnicity (defined from genetic data) adjusting for age, recruitment clinic, and other confounding variables that may be identified from the initial inspection of the data. Disease presentation and endometriosis severity between regions and ethnic groups in India will be analysed. Co-morbidity and environmental exposures between regions will be compared and correlated with disease severity and distribution of lesions. Genotype data will be used to define ethnicity for the phenotypic analysis. Association between previously identified endometriosis risk loci and endometriosis status in our Indian cohort will be analysed on imputed genotypes using SNPTEST v2. An additive logistic regression model will be used to test association whilst

For peer review only - http://bmjopen.bmj.com/site/about/guidelines.xhtml

controlling for population stratification by including age and the top 5 principle components as covariates in the model. A study-wide significance threshold of $p<0.05/n$ will be applied where n is the number of loci tested. Subsequently, we will perform a genome-wide association analysis using SNPTEST to estimate adjusted odds ratios and corresponding 95% CI between each available SNP and endometriosis case/control status. The sub-phenotype association analysis will be performed for rAFS stage, site, geographical regions (North, East, North East, South, West, and Central India), infertility, and associated comorbidities (reproductive, metabolic, inflammatory, autoimmune disorders, COVID-19, etc.).

Functional studies aimed at delineating the causal genetic variants and biological mechanisms underlying the observed statistical associations with endometriosis risk will be carried based on the results of the discovery GWAS. Meta-analysis in collaboration with IEGC and other Genomic groups will be explored.

Plans for data management, curation, storage, and confidentiality

Clinical and genomic datasets from Indian women with and without endometriosis will be captured in a central database (http://app.ecgri.in/login), maintained, and regularly updated at ICMR-NIRRH. The database is developed on a robust platform including the ability to allow online, real-time data entry. Demographic data will be kept in separate tables and records identifying the study participants will be kept confidential. Data entry and data access will be independently controlled and identifiable data held behind secure firewalls according to the ICMR- National Guidelines for Biomedical and Health Research Involving Human Participants [27]. Data collected from different sites will be held separately and only specific individuals within a research team will be able to access and edit data. Where possible, data will be captured using online tools, including the clinical data in the WERF-EPHect instruments. Data collected from paper questionnaires will be entered and verified

For peer review only - http://bmjopen.bmj.com/site/about/guidelines.xhtml

independently. The quality of the clinical and genomic data will be regularly monitored. The genotype data will be stored in a separate computer with sufficient memory space and backup data will be stored in hard disks. The clinical and genomic data will be carefully linked by individual identification numbers to maintain the confidentiality of the participants and the key will be held by RG at ICMR-NIRRH.

Ethics and dissemination

The study has ethics approval and will be monitored by the Institutional Ethics Committee (IEC) of the study sites and ICMR-NIRRH. The study protocol will also be monitored by the Health Ministry Screening Committee (HMSC) of the Government of India. Periodic progress reports are required to be submitted to HMSC, IEC of ICMR-NIRRH, and IECs of study sites. A periodic progress report will be submitted to the funding agency.

Publications

Results will be presented and discussed with endometriosis patient groups in India and elsewhere through Endometriosis Society India (ESI) and World Endometriosis Society (WES). RG and GWM are currently actively interacting and communicating with the World Endometriosis Society (WES) and World Endometriosis Research Foundation (WERF). The research findings from the study will be published in peer-reviewed, high-impact factor journals. The findings will be disseminated at national and international conferences.

Author contributions

RG conceived the study, designed the final study protocol, provided the domain knowledge expertise, coordinated with all site investigators, obtained the ethics approval, drafted the manuscript, revised and is the principal investigator of the grant; GWM helped in the design of the final study protocol, provided domain knowledge expertise, contributed in revision of the manuscript draft. MP, PP, SS, KP, PDM, BJ, CS, NB, KK, NS, AH, SM, MS, US, AS, NW, SC provided clinical inputs, coordinated ethics approval, recruited study participants,

For peer review only

For peer review only - http://bmjopen.bmj.com/site/about/guidelines.xhtml

obtained consents from the study participants, collected data, samples and involved in quality control and data interpretation. AB contributed to the development and piloting of electronic database, conducted quality controls, data analysis, and interpretation. GDM provided the bio statistical and epidemiological support for the study protocol and data analysis. SDM contributed to the development of an electronic database and provided technical and administrative support for the study. All authors read and approved the final manuscript.

Funding

This work was supported by the DBT Wellcome Trust India Alliance Fellowship (Grant no. IA/CPHI/18/1/503933) awarded to Rahul K Gajbhiye. The sponsor was not involved in the study design, in the writing of the report, and in the decision to submit the paper for publication

Competing interests

None declared.

Word Count: 4157

Ethics approval:

Ethics approvals have been granted by ICMR-NIRRH (D/ICEC/Sci-87/91/2019; D/ICEC/Sci-165/170/2020) and Institutional Ethics Committees of study sites (ECR/146/Inst/KA/2013/RR-19; AMC/EC/1304; AIIMS/IEC/2019-20/884; CH/IEC/19/004; 734/IEC/-AIIMSRPR/2019; IEC-NWMH/AP/2019/033-V1; 1320/IEC-AIIMSRPR/2020; /839EC/Pharmac/GMC/NGP/; ECR/262/Inst/UP/2013/RR-19). The study has been approved by the Health Ministry Screening Committee (HMSC) of the Government of India. (HMSC No.: 2019-7620).

Data sharing statement

Data sharing not applicable as no datasets generated and/or analysed for this study

Acknowledgments

The authors thank the members of the ECGRI study group. Dr. Shailaja Patil, Dr. Manisha Shembekar, Dr. Parul Sharma Saoji, Jishna RP, Nimisha Bhuyan, Akhila S., Kiran Kharsodiya,

For peer review only - http://bmjopen.bmj.com/site/about/guidelines.xhtml

Ashwini Patel, Merlin Pious, Aarti Kushwaha, Sudeshna Bakshi, Akshatha Shetty, Nadha Abdul Razack, Neha Yadav are sincerely acknowledged for assistance in data collection. Mr. Ram Barai is acknowledged for assistance in ECGRI electronic database development. Dr. Yogesh Kalkonde and Dr. Rajesh Dikshit are acknowledged for critical inputs on the study design. Data collection was facilitated by and conducted in compliance with the World Endometriosis Research Foundation Endometriosis Phenome and Biobanking Harmonisation Project (WERF EPHect). This work was supported by the DBT Wellcome Trust India Alliance Fellowship [Grant no. IA/CPHI/18/1/503933] awarded to Rahul K Gajbhiye.

References

1. Zondervan KT, Becker CM, Missmer SA. Endometriosis. *N Engl J Med* 2020; **382:**1244–56. DOI: 10.1056/NEJMra1810764
2. Population ages 15-64 (% of total population) | Data. https://data.worldbank.org/indicator/SP.POP.1564.TO.ZS (accessed 3 Feb 2021).
3. Population, total - India | Data. https://data.worldbank.org/indicator/SP.POP.TOTL?locations=IN (accessed 25 Jan 2021).
4. Rogers PAW, Adamson GD, Al-Jefout M, *et al.* Research Priorities for Endometriosis: Recommendations From a Global Consortium of Investigators in Endometriosis. *Reprod Sci* 2017;**24:**202–26. doi: 10.1177/1933719116654991
5. Grimstad FW, Decherney A. A Review of the Epigenetic Contributions to Endometriosis: *Clin Obstet Gynecol* 2017;**60:**467–76. doi: 10.1097/GRF.0000000000000298.
6. Koninckx PR, Ussia A, Adamyan L, *et al.* Pathogenesis of endometriosis: the genetic/epigenetic theory. *Fertil Steril* 2019;**111**:327–40. doi: 10.1016/j.fertnstert.2018.10.013.

For peer review only - http://bmjopen.bmj.com/site/about/guidelines.xhtml

7. Rahmioglu N, Macgregor S, Drong AW, *et al.* Genome-wide enrichment analysis between endometriosis and obesity-related traits reveals novel susceptibility loci. *Hum Mol Genet* 2015;**24**:1185–99. doi: 10.1093/hmg/ddu516.

8. Fung JN, Mortlock S, Girling JE, *et al.* Genetic regulation of disease risk and endometrial gene expression highlights potential target genes for endometriosis and polycystic ovarian syndrome. *Sci Rep* 2018; **8:**11424. doi: 10.1038/s41598-018-29462-y.

9. Gajbhiye R, Fung JN, Montgomery GW. Complex genetics of female fertility. *NPJ Genom Med* 2018;**3**:29. doi: 10.1038/s41525-018-0068-1.

10. Montgomery GW, Giudice LC. New Lessons about Endometriosis — Somatic Mutations and Disease Heterogeneity. *N Engl J Med* 2017;**376**:1881–2. doi: 10.1056/NEJMe1701700.

11. Gajbhiye R, Bendigeri T, Ghuge A, *et al.* Panel of Autoimmune Markers for Noninvasive Diagnosis of Minimal–Mild Endometriosis: A Multicenter Study. *Reprod Sci* 2017;**24**:413–20. doi: 10.1177/1933719116657190.

12. Gajbhiye R, Sonawani A, Khan S, *et al.* Identification and validation of novel serum markers for early diagnosis of endometriosis. *Hum Reprod* 2012;**27**:408–17. doi: 10.1093/humrep/der410.

13. Gajbhiye R, McKinnon B, Mortlock S, *et al.* Genetic Variation at Chromosome 2q13 and Its Potential Influence on Endometriosis Susceptibility Through Effects on the IL-1 Family. *Reprod Sci* 2018; 25(9):1307-1317. doi: 10.1177/1933719118768688.

14. Farland LV, Shah DK, Kvaskoff M, *et al.* Epidemiological and Clinical Risk Factors for Endometriosis. In: D'Hooghe T, ed. *Biomarkers for Endometriosis: State of the Art.* Cham: : Springer International Publishing 2017. 95–121.

For peer review only

For peer review only - http://bmjopen.bmj.com/site/about/guidelines.xhtml

15. Shafrir AL, Farland LV, Shah DK, *et al.* Risk for and consequences of endometriosis: A critical epidemiologic review. *Best Pract Res Clin Obstet Gynaecol* 2018;**51**:1–15. doi: 10.1016/j.bpobgyn.2018.06.001.

16. Zondervan KT, Becker CM, Koga K, *et al.* Endometriosis. *Nat Rev Dis Primers* 2018;**4**:9. doi: 10.1038/s41572-018-0008-5.

17. Treloar SA, O'Connor DT, O'Connor VM, *et al.* Genetic influences on endometriosis in an Australian twin sample. *Fertil Steril* 1999;**71**:701–10. doi: 10.1016/s0015-0282(98)00540-8.

18. Saha R, Pettersson HJ, Svedberg P, *et al.* Heritability of endometriosis. *Fertil Steril* 2015;**104**:947–52. doi: 10.1016/j.fertnstert.2015.06.035

19. Fung JN, Montgomery GW. Genetics of endometriosis: State of the art on genetic risk factors for endometriosis. *Best Pract Res Clin Obstet Gynaecol* 2018;**50**:61–71. doi: 10.1016/j.bpobgyn.2018.01.012.

20. Sapkota Y, Steinthorsdottir V, Morris AP, *et al.* Meta-analysis identifies five novel loci associated with endometriosis highlighting key genes involved in hormone metabolism. *Nat Commun* 2017;**8**:15539. doi: 10.1038/ncomms15539.

21. Montgomery GW, Mortlock S, Giudice LC. Should Genetics Now Be Considered the Pre-eminent Etiologic Factor in Endometriosis? *J Minim Invasive Gynecol. 2020;**27**:280-6. doi: 10.1016/j.jmig.2019.10.020.*

22. Nilufer R, Karina B, Paraskevi C, *et al.* Large-scale genome-wide association meta-analysis of endometriosis reveals 13 novel loci and genetically-associated comorbidity with other pain conditions. *bioRxiv* 2018:406967. doi: https://doi.org/10.1101/406967.

23. Bendigeri T, Warty N, Sawant R, *et al.* Endometriosis: Clinical Experience of 500 Patients from India. *The Indian Practioner* 2015;**68**:34-40.

For peer review only

24. Vitonis AF, Vincent K, Rahmioglu N, *et al.* World Endometriosis Research Foundation Endometriosis Phenome and Biobanking Harmonization Project: II. Clinical and covariate phenotype data collection in endometriosis research. *Fertil Steril* 2014;**102**:1223–32. doi: 10.1016/j.fertnstert.2014.07.1244

25. Becker CM, Laufer MR, Stratton P, *et al.* World Endometriosis Research Foundation Endometriosis Phenome and Biobanking Harmonisation Project: I. Surgical phenotype data collection in endometriosis research. *Fertility and Sterility* 2014;**102**:1213–22. doi: 10.1016/j.fertnstert.2014.07.709.

26. Cardoso JV, Perini JA, Machado DE, et al. Systematic review of genome-wide association studies on susceptibility to endometriosis. *Eur J Obstet& Gynecol and Reprod Biol* 2020;**255**:74–82. doi:10.1016/j.ejogrb.2020.10.017

27. Mathur R, Swaminathan S. National ethical guidelines for biomedical & health research involving human participants, 2017: A commentary. *Indian J Med Res. 2018 ;148:279-83. doi: 10.4103/0971-5916.245303.*

28. Rahmioglu N, Fassbender A, Vitonis AF, *et al.* World Endometriosis Research Foundation Endometriosis Phenome and Biobanking Harmonization Project: III. Fluid biospecimen collection, processing, and storage in endometriosis research. *Fertil Steril* 2014;**102**:1233–43. doi: 10.1016/j.fertnstert.2014.07.1208.

Figure Legends

Figure 1: Study sites of ECGRI across different geographic regions of India

Figure 2: Flow chart of ECGRI study participant recruitment and study processes.

Abbreviations: WERF-EPHect, World Endometriosis Research Foundation – Endometriosis Phenome and Biobanking Harmonisation Project; EPQ, Endometriosis Patient Questionnaire; SSF, Standard Surgical Form; SOP, Standard Operating Processes; QC, Quality Control;

For peer review only - http://bmjopen.bmj.com/site/about/guidelines.xhtml

619 ICMR-NIRRH, Indian Council of Medical Research-National Institute for Research in
620 Reproductive Health.
621
622
623
624
625

For peer review only

For peer review only - http://bmjopen.bmj.com/site/about/guidelines.xhtml

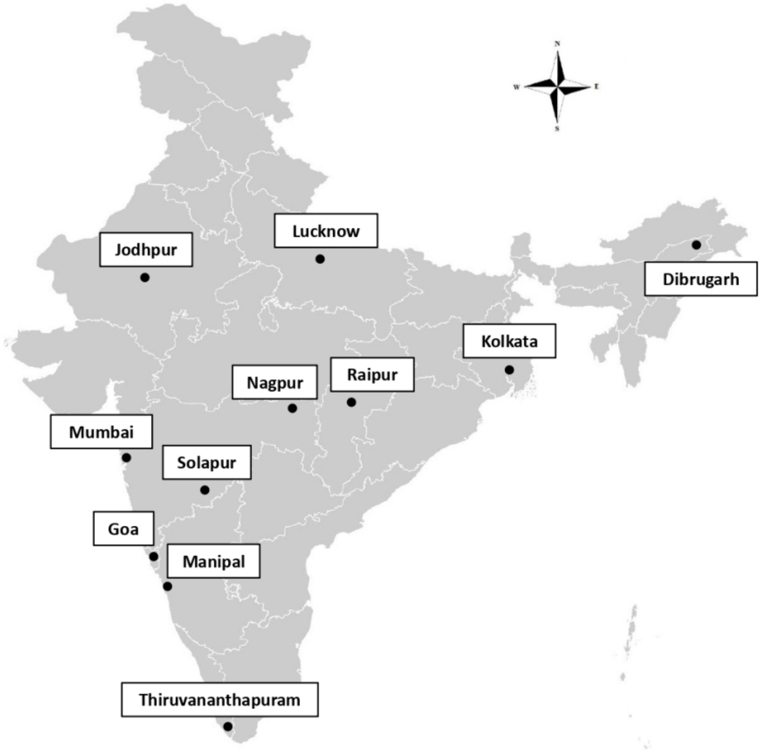

State	Study sites	State	Study sites
Assam	Assam Medical College, Dibrugarh	Maharashtra	Worli Hospital for Women, Mumbai; Nowrosjee Wadia Maternity Hospital, Mumbai; Naval Maternity Endoscopy & Infertility Center, Mumbai
Uttar Pradesh	King George's Medical University, Lucknow; Morpheus Lucknow Fertility Center, Lucknow		
Rajasthan	All India Institute of Medical Sciences (AIIMS), Jodhpur		
West Bengal	Spectrum Clinic and Endoscopy Research Institute, Kolkata	Maharashtra	Naval Maternity Endoscopy & Infertility Center, Solapur
Chhattisgarh	AIIMS, Raipur; Sai Baba Nursing Home, Raipur	Karnataka	Kasturba Medical College, Manipal
Maharashtra	Government Medical College, Nagpur; Omega Hospital, Nagpur	Kerala	Credence Hospital, Thiruvananthapuram

Study sites of ECGRI across different geographic regions of India

95x137mm (300 x 300 DPI)

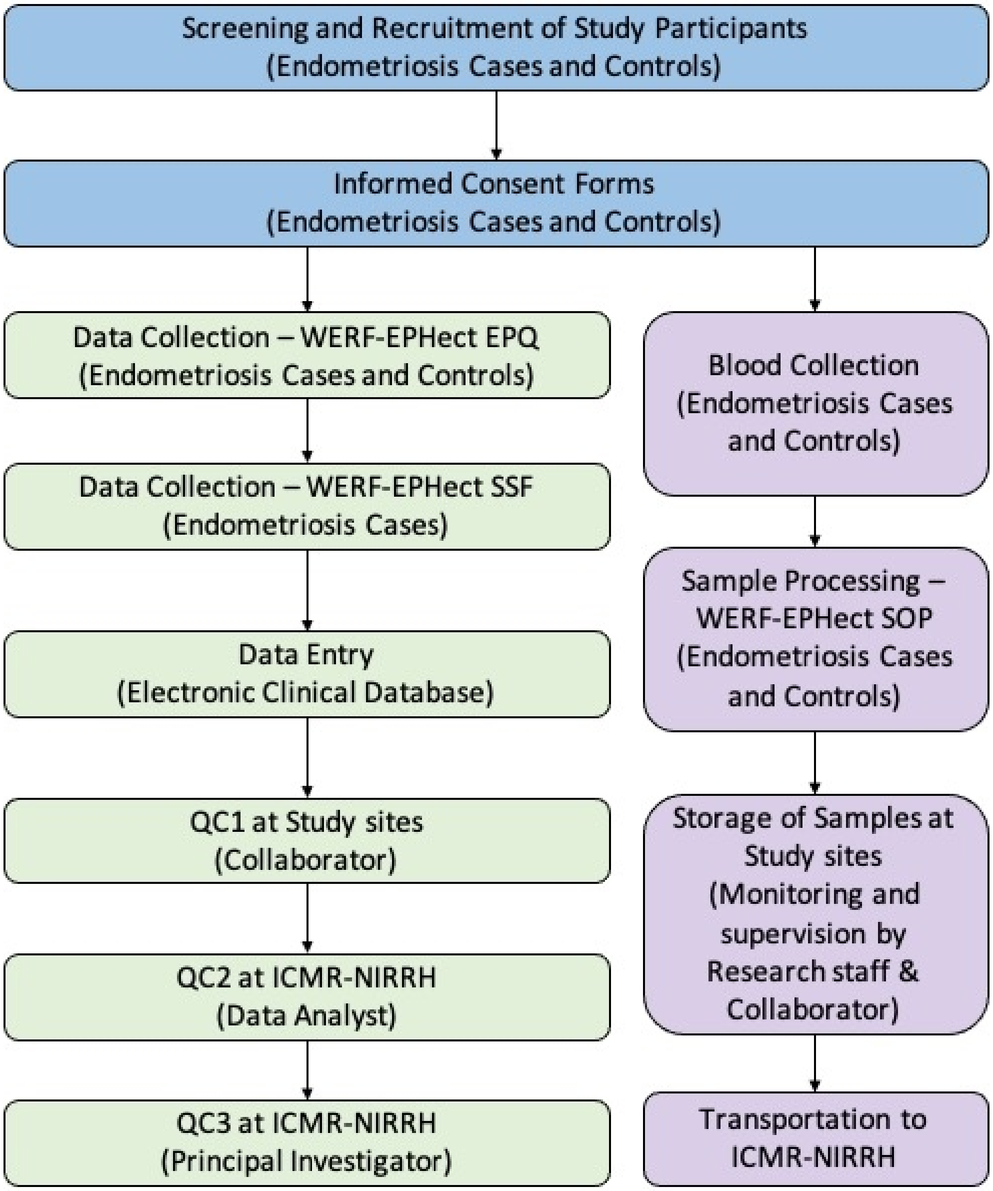

Flow chart of ECGRI study participant recruitment and study processes.
Abbreviations: WERF-EPHect, World Endometriosis Research Foundation – Endometriosis Phenome and Biobanking Harmonisation Project; EPQ, Endometriosis Patient Questionnaire; SSF, Standard Surgical Form; SOP, Standard Operating Processes; QC, Quality Control; ICMR-NIRRH, Indian Council of Medical Research – National Institute for Research in Reproductive Health.

41x50mm (300 x 300 DPI)

Supplementary Table 1: Clinical, epidemiological, reproductive, lifestyle and environmental characteristics captured using modified Endometriosis Patient Questionnaire (EPQ)

**Characteristics**	**Variables**
**Menstrual and Reproductive factors**	Age at menarche, menstrual cycle length, menstrual volume, dysmenorrhea, current oral contraceptive use, hormones, history of infertility
**Pregnancy and Fertility**	Age at each pregnancy, parity, fertility treatment, outcome of pregnancy, history of miscarriage, ectopic pregnancy, vaginal delivery, caesarean section, breast feeding, natural pregnancy/ ART, complications related to pregnancy- GDM, PIH, Preterm birth
**Health-related Quality of life**	Thoughts and feelings associated with pain, severity grading scale (0-10), Life time details - pelvic pain, pain during or after vaginal intercourse, treatment for pain, gastrointestinal and urinary symptoms etc.
**Socio-demographic, personal and lifestyle factors**	Geographic location, age, educational level, marital status, age at marriage, employment, family income, height, weight, BMI, waist -to- hip ratio, ethnicity, cigarette smoking, alcohol use, caffeine intake, regular exercise, night shift work, natural hair color, eye color, skin color, body shape
**Medical and surgical**	History of associated diseases- cancer, autoimmune diseases<br>endometriosis or chronic pelvic pain history- personal and family (if known), details of medical and surgical treatment
**Environmental factors**	Residence in each decade of life, pollution exposure, smoking environment,<br>sun exposure, occupational history, pesticide exposure, any other relevant information

Abbreviations: ART, Assisted Reproductive Technology; GDM, Gestational Diabetes Mellitus; PIH, Pregnancy-induced hypertension; BMI, Body mass index

For peer review only
