# Supplementary material for: Protocol for a case–control study investigating the clinical phenotypes and genetic regulation of endometriosis in Indian women: the ECGRI study
Source: BMJ Open. 2021 Aug 9;11(8):e050844. doi: 10.1136/bmjopen-2021-050844 (PMC8354274; doi:10.1136/bmjopen-2021-050844)
Supplement: Reviewer comments [file bmjopen-2021-050844.reviewer_comments.pdf]

PEER REVIEW HISTORY

BMJ Open publishes all reviews undertaken for accepted manuscripts. Reviewers are asked to complete a checklist review form (**http://bmjopen.bmj.com/site/about/resources/checklist.pdf**) and are provided with free text boxes to elaborate on their assessment. These free text comments are reproduced below.

ARTICLE DETAILS

**TITLE (PROVISIONAL)**	Protocol for a case-control study investigating the clinical phenotypes and genetic regulation of endometriosis in Indian women: the ECGRI study
**AUTHORS**	Gajbhiye, Rahul; Montgomery, Grant; Pai, Murlidhar; Phukan, Pranay; Shekhar, Shahank; Padte, Kedar; Dasmahapatra, Pramathes; John, Bimal; Shembekar, Chaitanya; Bhurke, Aishwarya; Bagde, Nilajkumar; Kulkarni, Ketki; Sardeshpande, Nagendra; Humne, Anil; Mahobia, Swati; Shah, Millind; Singh, Uma; Shrivastav, Arti; Mishra, Gita; Warty, Neeta; Chandra, Sunita; mahale, smita

VERSION 1 – REVIEW

**REVIEWER**	Parazzini, Fabio<br>University of Milan
**REVIEW RETURNED**	05-May-2021

**GENERAL COMMENTS**	This is an interesting study conducted in India, a Country rarely considered in the studies on endometriosis.<br><br>The authors should indicate clearly the main end point of the study Specifically i suggest that the authors should indicate in the outcomes section the main end point. Further, the sample size should be specified according to the main end point.

**REVIEWER**	Rocha, Rodrigo<br>The University of Sydney, Gynaecology and Obstetrics
**REVIEW RETURNED**	19-May-2021

**GENERAL COMMENTS**	Protocol for a case-control study investigating the clinical phenotypes and genetic regulation of endometriosis in Indian women: the ECGRI study<br><br>This study protocol follows high standards of clinical information collection and sample processing and storage, following the recommendations of the World Endometriosis Research Foundation Endometriosis Phenome and Biobanking Harmonization Project (WERF-EPHect), with a clear objective of identifying genetic risk factors associated with endometriosis through genome-wide association (GWA) analysis. The authors followed the framework of other international groups in the field, confirming its methods, precise and reproducible research. To the best of my knowledge, the procedures and techniques involved in the analysis of the biological material collected are in accordance with the most recent literature on the subject.

	This protocol is robust in selecting the cases, including surgery results for confirming the disease, and standardization of the clinical questionnaires, using the WERF-EPHect as the primary data and sample collection instruments. In addition, it aims to represent a significant segment of the world`s population, aiming for an adequate sample size and significant representation of the regional population. It will add a remarkable contribution to the body of evidence among the GWA studies.<br>Recently, in a meta-analysis on this subject, Cardoso et al.1 identified 15 GWA publications on endometriosis; however, the results are somewhat conflicting. The authors attributed the conflicting results associated with sample size, power of analysis, selection criteria for cases and controls, and differences in allelic frequencies among studied populations. The inclusion of that reference and analysis in the discussions of the strengths and limitations of this particular study would be suggested. Although extensive, the WERF EPHect Standard Surgical Form (SSF) does not include confirming the lesions via histopathology analysis. Whilst this feature might not be relevant in the context of an advanced stage of endometriosis, it might cause the inclusion of patients of suspected superficial endometriosis (SUP) by visual impression at laparoscopy with no histopathology correspondence. Schliep et al.2 observed a 20% reduction in expert agreement from a positive to a negative diagnosis after histopathology results disclosure. Additionally, it would be valuable a possible clarification if the authors considered the possibility of excluding patients from the control group, i.e. patients with solid clinical evidence of possible endometriosis after analysis of the responses of the WERF-EPHect EPQ. Taylor et al.3 highlighted the importance of clinical features in the presumptive diagnosis of endometriosis.<br>It would be of extreme interest to the scientific community to access the results of such relevant research. Great appreciation is given to have the opportunity of providing constructive feedback.<br>References:<br>1. Cardoso JV, Perini JA, Machado DE, Pinto R, Medeiros R. Systematic review of genome-wide association studies on susceptibility to endometriosis. Eur J Obstet Gynecol Reprod Biol 2020; 255: 74–82.<br>2. Schliep KC, Chen Z, Stanford JB, Xie Y, Mumford SL, Hammoud AO, Boiman Johnstone E, Dorais JK, Varner MW, Buck Louis GM, Peterson CM. Endometriosis diagnosis and staging by operating surgeon and expert review using multiple diagnostic tools: an inter-rater agreement study. BJOG An Int J Obstet Gynaecol 2017; 124: 220–9.<br>3. Taylor HS, Adamson GD, Diamond MP, Goldstein SR, Horne AW, Missmer SA, Snabes MC, Surrey E, Taylor RN. An evidence-based approach to assessing surgical versus clinical diagnosis of symptomatic endometriosis. Int J Gynecol Obstet 2018; 142: 131–42.

VERSION 1 – AUTHOR RESPONSE

Reviewer: 1

Dr. Fabio Parazzini, University of Milan

Comments to the Author:
This is an interesting study conducted in India, a Country rarely considered in the studies on endometriosis.
The authors should indicate clearly the main end point of the study
Specifically i suggest that the authors should indicate in the outcomes section the main end point. Further, the sample size should be specified according to the main end point.

Response : Endopoints for the study are clinical and phenotypic differences in presentation of endometriosis at different clinics and different regions of India, replication in the Indian population of genetic risk factors for endometriosis discovered in European populations.
Secondary endpoints will be possible discovery of population specific common risk factors for endometriosis and if there are significant differences in the clinical presentation of endometriosis in different regions of India, we will conduct secondary analyses, making use of the genetic information to define ethnic subgroups across India, to evaluate whether these differences might result from ethnic or environmental differences between regions. The power and sample size have been determined to meet the primary endpoints.
We have revised the oucomes section as per the reviewer suggestion.

Reviewer: 2
Dr. Rodrigo Rocha, The University of Sydney

Comments to the Author:
Protocol for a case-control study investigating the clinical phenotypes and genetic regulation of endometriosis in Indian women: the ECGRI study

This study protocol follows high standards of clinical information collection and sample processing and storage, following the recommendations of the World Endometriosis Research Foundation Endometriosis Phenome and Biobanking Harmonization Project (WERF-EPHect), with a clear objective of identifying genetic risk factors associated with endometriosis through genome-wide association (GWA) analysis. The authors followed the framework of other international groups in the field, confirming its methods, precise and reproducible research. To the best of my knowledge, the procedures and techniques involved in the analysis of the biological material collected are in accordance with the most recent literature on the subject.
This protocol is robust in selecting the cases, including surgery results for confirming the disease, and standardization of the clinical questionnaires, using the WERF-EPHect as the primary data and sample collection instruments. In addition, it aims to represent a significant segment of the world`s population, aiming for an adequate sample size and significant representation of the regional population. It will add a remarkable contribution to the body of evidence among the GWA studies. Recently, in a meta-analysis on this subject, Cardoso et al.1 identified 15 GWA publications on endometriosis; however, the results are somewhat conflicting. The authors attributed the conflicting results associated with sample size, power of analysis, selection criteria for cases and controls, and differences in allelic frequencies among studied populations. The inclusion of that reference and analysis in the discussions of the strengths and limitations of this particular study would be suggested.
Response: We thank the reviewer for appreciating our study and providing valuable suggestions.
As suggested we have added reference of Cardoso et al., 2020.

Page no. 21, line no. 498-502.

Although extensive, the WERF EPHect Standard Surgical Form (SSF) does not include confirming the lesions via histopathology analysis. Whilst this feature might not be relevant in the context of an advanced stage of endometriosis, it might cause the inclusion of patients of suspected superficial endometriosis (SUP) by visual impression at laparoscopy with no histopathology correspondence. Schliep et al.2 observed a 20% reduction in expert agreement from a positive to a negative diagnosis after histopathology results disclosure.
Response: We agree and aware that the WERF EPHect Standard Surgical Form (SSF) does not include confirming the lesions via histopathology analysis. Thank you for providing the reference of Schliep et al. We will be confirming the endometriotic lesions on histopathology wherever possible. This is already mentioned in the manuscript page no. 11, line no. 254-255.

Additionally, it would be valuable a possible clarification if the authors considered the possibility of excluding patients from the control group, i.e. patients with solid clinical evidence of possible endometriosis after analysis of the responses of the WERF-EPHect EPQ. Taylor et al.3 highlighted the importance of clinical features in the presumptive diagnosis of endometriosis.
Response: We thank you for the valuable comment and suggestion on the selection of control group. We appreciate providing the reference of Taylor et al.
While recruiting the hospital controls, we will be taking a detailed clinical history on pain symptoms, history of endometriosis (personal and family). After initial screening on clinical history, if we do not find any clinical evidence suggestive of endoemtriosis then we enroll the participant as a control. So the participant with possible endometriosis suspected on clinical history is excluded at initial screening before the data collection as per WERF-EPQ.

It would be of extreme interest to the scientific community to access the results of such relevant research. Great appreciation is given to have the opportunity of providing constructive feedback.
References:
1. Cardoso JV, Perini JA, Machado DE, Pinto R, Medeiros R. Systematic review of genome-wide association studies on susceptibility to endometriosis. Eur J Obstet Gynecol Reprod Biol 2020; 255: 74–82.
2. Schliep KC, Chen Z, Stanford JB, Xie Y, Mumford SL, Hammoud AO, Boiman Johnstone E, Dorais JK, Varner MW, Buck Louis GM, Peterson CM. Endometriosis diagnosis and staging by operating surgeon and expert review using multiple diagnostic tools: an inter-rater agreement study. BJOG An Int J Obstet Gynaecol 2017; 124: 220–9.
3. Taylor HS, Adamson GD, Diamond MP, Goldstein SR, Horne AW, Missmer SA, Snabes MC, Surrey E, Taylor RN. An evidence-based approach to assessing surgical versus clinical diagnosis of symptomatic endometriosis. Int J Gynecol Obstet 2018; 142: 131–42.

Response: Thank you for the appreciation and for provding suggestions and relevant references.
